# Neuromodulating Alkaloids from Millipede Defensive Secretions

**DOI:** 10.1021/acs.jnatprod.4c01162

**Published:** 2024-12-30

**Authors:** Carla Menegatti, Jared S. Wood, Paige Banks, Kenneth Knott, Jonathan S. Briganti, Anthony J. Briganti, Samuel V. G. McNally, Paul E. Marek, Anne M. Brown, Tappey H. Jones, R. Thomas Williamson, Emily Mevers

**Affiliations:** † Department of Chemistry, Virginia Tech, Blacksburg, Virginia 24061, United States; ‡ Department of Chemistry & Biochemistry, University of North Carolina Wilmington, Wilmington, North Carolina 28403, United States; § Department of Biochemistry, Virginia Tech, Blacksburg, Virginia 24061, United States; ⊥ University Libraries, Virginia Tech, Blacksburg, Virginia 24061, United States; ¶ Condor Country Consulting, Inc, Martinez, California 94553, United States; ∥ Department of Entomology, Virginia Tech, Blacksburg, Virginia 24061, United States; # Department of Chemistry, Virginia Military Institute, Lexington, Virginia 24450, United States

## Abstract

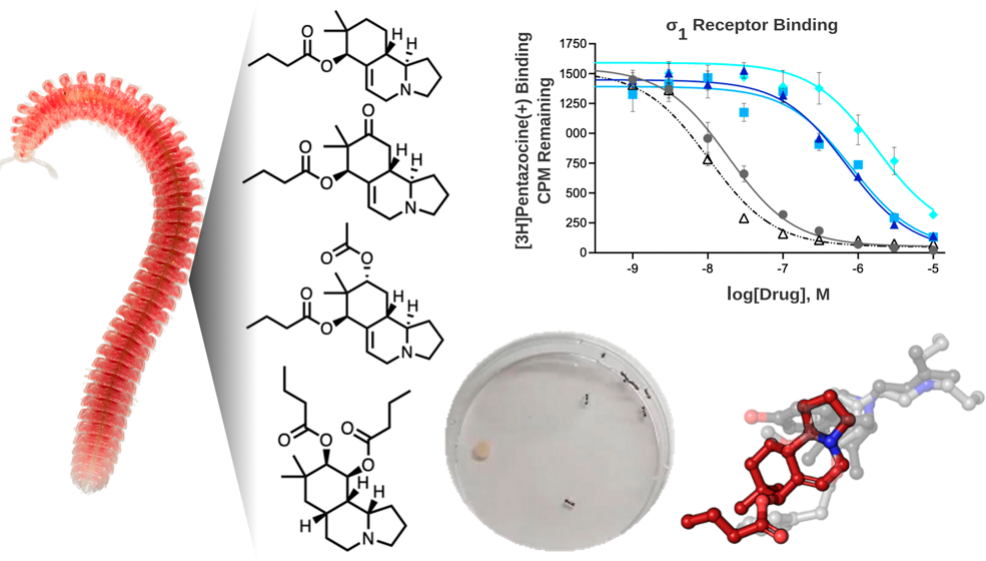

Millipedes have long been known to produce structurally diverse chemical defenses, including hydrogen cyanide, terpenoid alkaloids, and oxidized aromatics. Although the hydrogen cyanide and oxidized aromatic producing millipedes have been well studied, less than 10% of the terpenoid alkaloid producers have been chemically investigated. Several previous studies have shown that alkaloids disorient predators, but their biochemical target is currently unknown. Herein, we investigated the defensive secretions of a colobognath millipede, *Ischnocybe plicata*, and elucidated the constitution, absolute configuration, and conformation of four new highly oxidized terpenoid alkaloids, termed ischnocybines, using a range of analytical techniques. The ischnocybines are actively secreted from the defensive glands and were shown to disorient ants, a likely common predator. Evaluation of the ischnocybines in a panel of neuroreceptors revealed that ischnocybine A possesses potent (K_i_ 13.6 nM) and selective (100-fold) binding affinity for sigma-1, an orphan neuroreceptor, over sigma-2. These molecules represent the most complex alkaloids to be discovered from millipedes and provide the first potential insights into a biochemical target responsible for their defensive properties.

Millipedes are a diverse class (Diplopoda) of arthropods that are known to produce distinct chemical defensive secretions, including alkaloids, oxidized aromatics, and hydrogen cyanide.^1,2^ These defensive compounds, or their biosynthetic precursors, are stored in high concentrations within repugnatorial glands and are released upon disturbance.^1^ Both hydrogen cyanide and oxidized aromatic containing secretions have reactive functional groups and are believed to deter predation as either a harmful toxin or general irritants, respectively.^3−5^ Alkaloid defensive secretions include both quinazolinone and terpenoid alkaloids, with the former hypothesized to represent a hybrid between the oxidized aromatics and cyanide-containing agents.^6^ Due to their structural intricacy, the terpenoid alkaloids are distinct from all other classes of millipede defensive compounds and represent the most complex secretions yet to be reported (i.e., larger molecular weight, contain stereocenters, and diverse functionality).^1,6−9^ Their defensive mechanism is unknown; however, it is unlikely that the terpenoid alkaloids function as general irritants as they lack the reactive functionalities. Two previous reports indicate that a subset of the terpenoid alkaloids cause disorientation in common predators, thus suggesting a potential neurological mechanism.^8,9^

All known terpenoid alkaloids are produced by millipedes within a single subterclass, Colobognatha (fungus-feeding millipedes), which consists of four orders (Platydesmida, Polyzoniida, Siphonocryptida and Siphonophorida).^1^ All four orders have been reported to produce simple monoterpenes, such as α-pinene, however three orders (Platydesmida, Polyzoniida and Siphonocryptida) are known to produce complex terpenoid alkaloids (Figure 1). Currently, 17 alkaloids have been described with 14 being reported recently (since 2020).^6−12^ This explosion in defensive secretion chemistry has provided insights into a potential biosynthetic route. All millipede terpenoid alkaloids are hypothesized to incorporate a nitrogen that is presumably derived from either an amino acid (lysine or ornithine) or cyanide. Structural diversity appears to arise from variations in the terpene cyclization patterns and postmodifications (e.g., oxidation, nitration, and ligation). Nearly all reported alkaloids from Polyzoniida and Siphonocryptida millipedes contain a spirocyclic core (polyzonimine-like), though there is some variation in the number of carbons incorporated into each core structure (10 to 13).^9−11^ Conversely, defensive secretions characterized from Platydesmida contain one of two core structures: a bicyclic indolizidine/quinolizidine core (gosodesmine-like) or a 5,6,6-fused tricyclic core (buzonamine-like).^6−8,12^ Both the gosodesmine- and buzonamine-like defensive secretions derive from a monoterpene, likely geranyl pyrophosphate, and either ornithine (5-membered rings) or lysine (6-membered rings). Finer structural diversity arises from oxidation events (e.g., formation of olefins and epoxides) that are likely installed after the cyclization.

**Figure 1:**
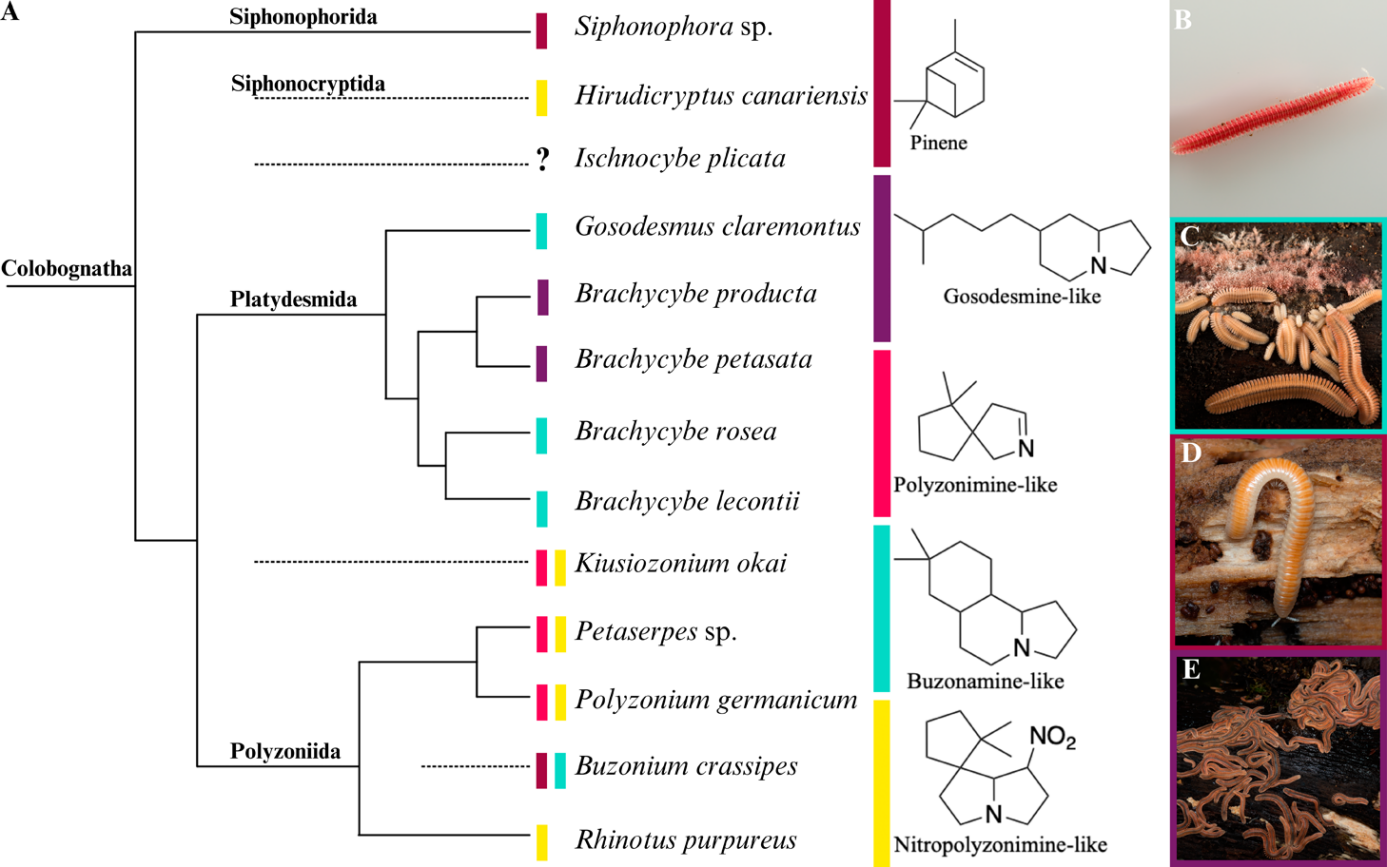
(A) Millipede phylogenetic tree for subterclass colobognath. The types of compounds produced by each millipede are shown.^17−19^ (B–E) Pictures of diverse Colobognatha millipedes. (B) *Ischnocybe plicata*; (C) *Brachycybe lecontii*; (D) *Buzonium crassipes*; (E) *Brachycybe producta*. Supertree from refs. (17)–^19^.

Although there has been a recent resurgence in chemistry being described from Colobognatha, only about 10% of the described species have been chemically studied. Nearly all of these studies have led to the discovery of new chemical compounds.^1,6,7,11^ Herein, we report chemical investigations into the defensive secretions of *Ischnocybe plicata* (Platydesmida, Andrognathidae), a millipede that resides in the U.S. Pacific Northwest (Figure 1), leading to full structural characterization of four highly functionalized terpenoid alkaloids. The structure elucidation of the ischnocybines (**1**–**4**) was accomplished using comprehensive analysis of 2D NMR, computational experimentation, electronic circular dichroism, and chemical derivatization. Notably, three of the ischnocybines potently and selectively bind sigma-1 receptor (σ_1_R), an orphan receptor.^13^ This receptor is a potential drug target for various disorders, and this is the first report of a molecular target for any of the millipede alkaloid defensive secretions.^14−16^

## Results and Discussion

Field observations suggested that when disturbed (shaken), *I. plicata* exudes a scent reminiscent of pine oil or citrus, indicating terpene production. GCMS analysis of a methanol extract of *I. plicata* collected in downed woody debris in Oregon forests revealed a mixture of monoterpenes and four larger metabolites that possessed fragmentation patterns that were dissimilar to known millipede alkaloids with molecular ions at *m*/*z* 291 (**1**), 305 (**2**), 349 (**3**) and 379 (**4**) (Figures S1–S5). Preliminary chemical derivatization of the methanol extract using hydrogenation, basic hydrolysis, and aminolysis revealed that **1**–**3** each possessed an olefin, **1** and **2** contained a single butyrate, and **3** and **4** contained a butyrate and either an acetate or another butyrate, respectively (Figures S6–S13). Additionally, **2** formed a methoxime, suggesting a ketone (Figure S14). Analysis of high resolution MS (HRMS) data indicated molecular formulas of C_18_H_29_NO_2_ (**1**, observed [M + H]^+^ 292.2277, calcd. C_18_H_30_NO_2_, Δ0.0 ppm), C_18_H_27_NO_3_ (**2**, observed [M + H]^+^ 306.2072, calcd. C_18_H_28_NO_3_, Δ0.9 ppm), C_20_H_31_NO_4_ (**3**, observed [M + H]^+^ 350.2335, calcd. C_20_H_32_NO_4_, Δ1.1 ppm), and C_22_H_37_NO_4_ (**4**, observed [M + H]^+^ 380.2794, calcd. C_22_H_38_NO_4_, Δ1.8 ppm) (Figures S15–S18). These key structural characteristics did not resemble known alkaloids previously isolated from millipedes. Therefore, the four metabolites present in the *I. plicata* extracts were purified via reverse phase high performance liquid chromatography (RP HPLC) and full 2D NMR data sets [^1^H, ^13^C, COSY, EASY-ROESY, HSQC and HMBC] were acquired on each metabolite to elucidate their planar structures (Figures S19–S42 and Tables S1–S4).

**Figure 2:**
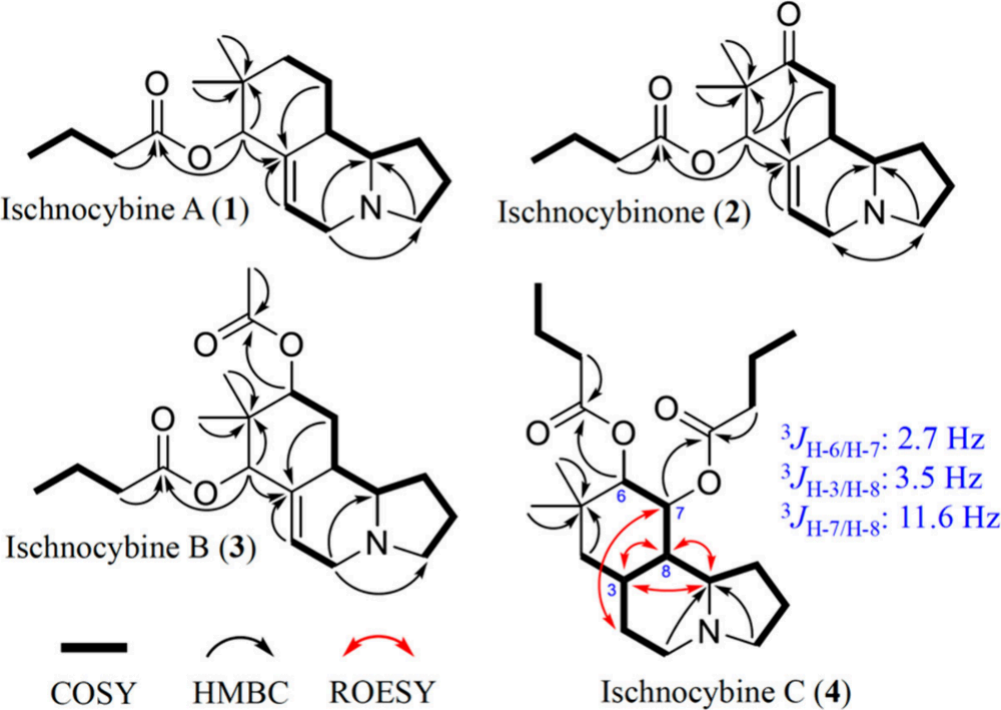
Key 2D NMR correlations and coupling constants.

We began the structure elucidation effort with **1**, as it had the simplest NMR data and smallest mass of the isolated metabolites. The molecular formula (C_18_H_29_NO_2_) indicated five degrees of unsaturation and analysis of the ^13^C NMR spectrum revealed the presence of four deshielded carbons—an ester (δ_C_ 172.2), two olefinic carbons (δ_C_ 135.4 and 126.3), and an oxygenated carbon (δ_C_ 80.1), accounting for two degrees of unsaturation. The ^1^H NMR spectrum confirmed a trisubstituted olefin with a single proton signal at δ_H_ 5.65, but also revealed two aliphatic singlet methyl groups (δ_H_ 23.8 and 27.2). The lack of additional functionality in the 1D NMR spectrum suggested that rings account for the remaining three degrees of unsaturation. Analysis of the 2D NMR spectra, particularly the HMBC and COSY (Figure 2), revealed that **1** is related to the known defensive metabolite buzonamine (**5**), isolated from the polyzoniid millipede *Buzonium crassipes*.^8^ Compound **1** contained a similar 5,6,6-fused tricyclic backbone with modifications in the B and C rings. In the B ring, the signals for an epoxide were clearly absent and replaced by two downfield signals that corresponded to an olefin. The olefinic proton (H-2, δ_H_ 5.65) exhibited HMBC correlations with C-1 (δ_C_ 52.1), C-4 (δ_C_ 80.1) and C-8 (δ_C_ 39.4). In addition, ring C had an additional oxidized carbon adjacent to the *gem*-dimethyl moiety. Analyzing HMBC correlations led to assignment of a butyrate moiety attached to C-4 (δ_C_ 80.1) and the planar structure of **1**.
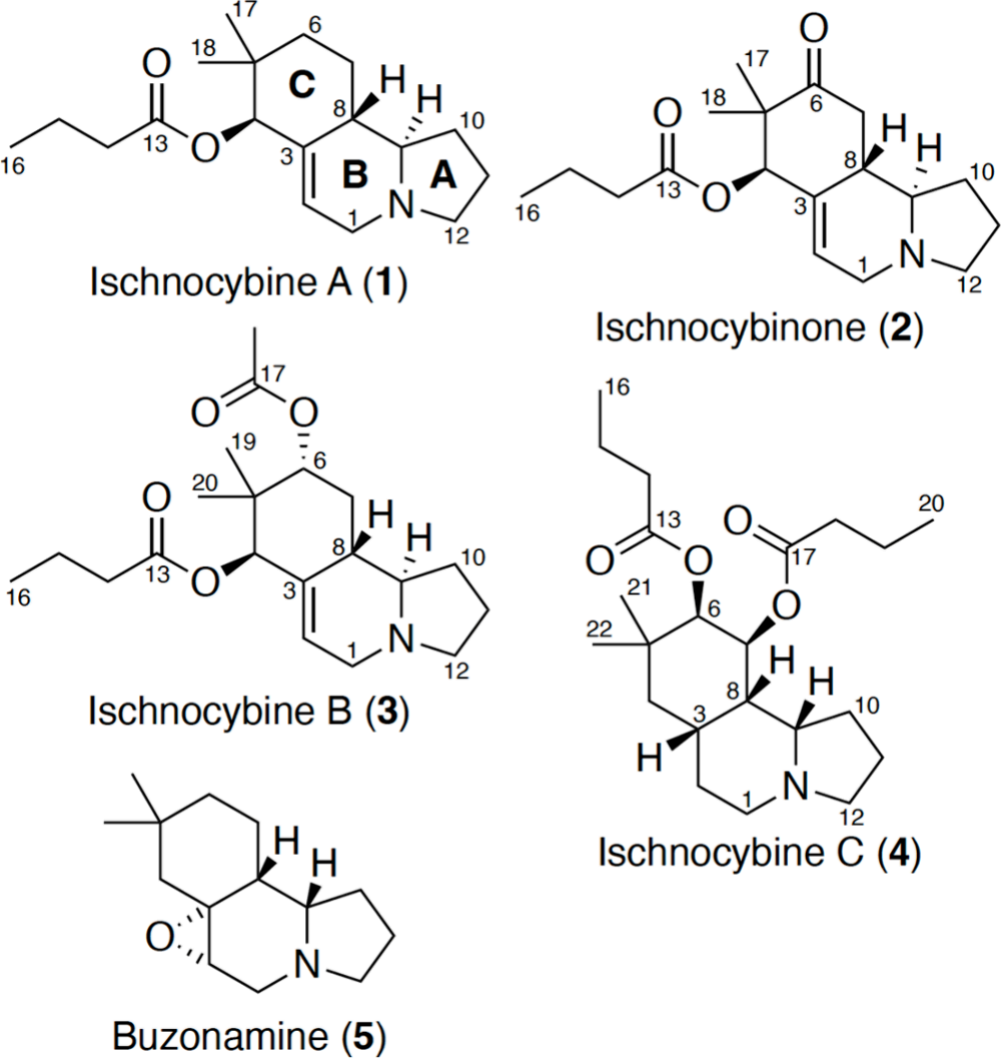


Compounds **2**–**4** shared a nearly identical carbon backbone with **1** but differed in oxidation state at two positions. The molecular formula of **2** indicated an additional oxygen atom and an additional degree of unsaturation. The 1D NMR spectra of **2** revealed no protons on C-6 and an additional strongly deshielded carbon signal (δ_C_ 211.7). Analysis of 2D NMR data confirmed C-6 as a ketone as both the *gem*-dimethyls (δ_H_ 0.92 and 1.08) exhibited HMBC correlations to C-6. Compound **3** incorporated an additional C_2_H_2_O_2_ compared to **1**. The ^1^H NMR spectra of **3** revealed an additional singlet methyl group (δ_H_ 2.02) and this chemical shift matched the expected shift for an acetate methyl. The presence of an acetate was also supported by an additional deshielded carbon (δ_C_ 170.6) in the HMBC, and by a prominent loss of 59 (corresponding to the formula CH_3_CO_2_) in the initial GCMS analysis. A key HMBC correlation between H-6 (δ_H_ 5.01) and C-17 (δ_C_ 170.6) further supported assignment of an acetate at C-6.

Though, the most dissimilar to **1**, **4** still contained the same tricyclic moiety. The molecular formula for **4** (C_22_H_37_NO_4_) included an additional C_2_H_6_ while also containing one less degree of unsaturation than **3**. Based on the GCMS analysis, **4** lacked the olefin between C-2/C-3 and contained two butyrates. Comparison of the ^13^C spectrum of **4** to those of **1**–**3** confirmed this; the spectrum of **4** lacked olefinic signals and had two carbonyl signals consistent with esters (δ_C_ 172.9 and 172.4). Both sets of *gem*-dimethyl protons shared HMBC correlations to C-6 (δ_C_ 76.5), from which one butyrate was attached. From C-6, HSQC and COSY data revealed the origin of the second butyrate at C-7 (δ_C_ 69.3). HMBC correlations from H-6 and H-7 to the ester carbonyls facilitated the placement of the butyrate groups. Thus, we deduced planar structures for ischnocybines A–C and ischnocybinone.

Relative configurations of **1**–**3** were established through a combination of ROESY NMR experiments and DELTA50^20^ based Density Functional Theory (DFT) calculations. For compound **1**, chemical shift predictions were carried out for all four possible stereoisomers. The 4*S*,8*S*,9*R* yielded the best results, with ^13^C and ^1^H RMSD values of 2.3 and 0.07 ppm, respectively. Based on a lack of apparent ROESY correlations between H-4, H-8, and H-9, comparisons to geometry-optimized structures of **1**, and chemical shift predictions, it was determined that **1** had a relative configuration of 4S*,8S*,9R* (Tables S5 and S6). Compounds **2** and **3** were similar to **1** based on observed ROEs and chemical shift predictions, and were assigned the relative configurations 4R*,8S*,9R* and 4R*,6R*,8S*,9R*, respectively (Tables S7–S10).

Analysis of **4** was not straightforward, given the significant dissimilarity to **1**. Conformational searches yielded far more results for **4** relative to **1**–**3** due to added flexibility at positions 2 and 3. For several possible stereoisomers, chemical shift predictions yielded similar results (Tables S11–S14); fortunately, ^1^H signals were relatively clean and fairly well-resolved, allowing for application of *J*-based configuration analysis. Protons H-7 and H-8 shared a large coupling constant of 11.6 Hz, pointing toward a *trans*-diaxial relationship (Figure 2). The small coupling constant between H-6 and H-7 (2.7 Hz) suggested that H-6 is equatorial; the same holds true for H-3 and H-8 (3.5 Hz), where H-3 is likely equatorial. Additionally, the intensity of the ROESY correlations between H-2, H-7, and H-21, and the correlations between H-3, H-8, and H-9 are highly indicative of the overall conformation of **4** (Figure 2). These correlations were extremely helpful for comparison with 3D models from computationally aided conformational searches. Overall, these data combined with chemical shift predictions facilitated assignment of the relative configuration of **4** as 4R*,6S*,7R*,8R*,9R*.

**Figure 3:**
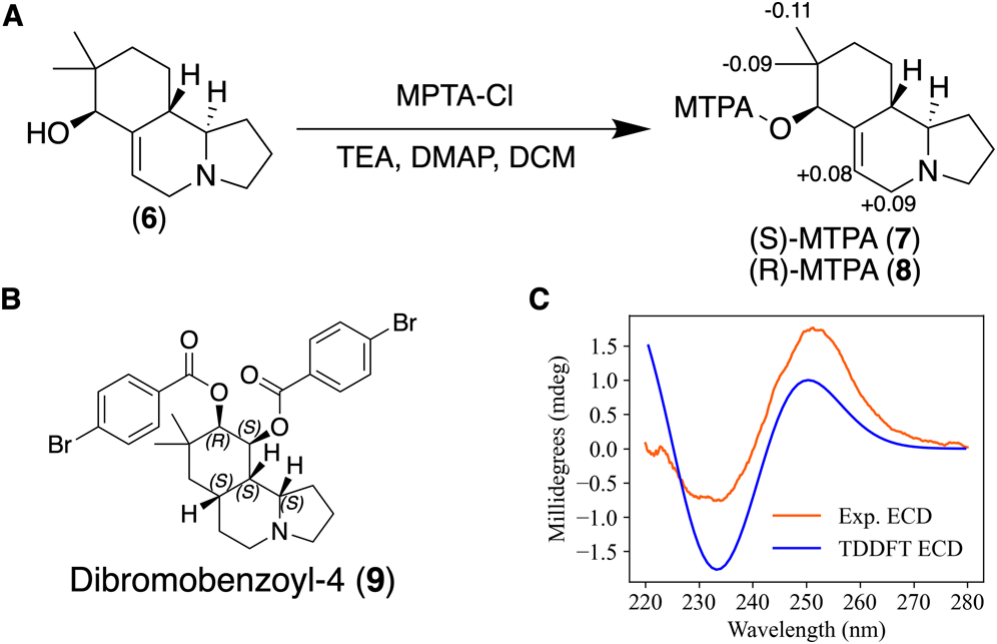
(A) Δδ_S-R_ values of the Mosher esters of the free alcohol of **1** (**6**), (B) 4*S*,6*R*,7*S*,8*S*,9*S* configuration of the dibromobennzoyl derivative of hydrolyzed **4** (**9**), (C) experimental and TD-DFT-predicted ECD data for **9**.

Absolute configuration assignments of **1**–**4** were conducted using a combination of Mosher esterification, electronic circular dichroism (ECD), and time-dependent (TD) DFT calculations. Fortuitously, several milligrams of the free alcohol of **1** (**6**; Figures S43–S48; Table S15) were obtained during purification of the October 2023 collection material and a portion of this material was derivatized with both (*R*)- and (*S*)-α-methoxy-α-trifluoromethylphenylacetic acid (MTPA).^21^ Analysis of the shielded and deshielded patterns in the derivatized products (compound **7** and **8**) for protons H-1a, H-1b, H-2, H-17, and H-18 (Figure 3A) confirmed an absolute configuration of 4*S*, thus indicating an absolute configuration of 4*S*,8*S*,9*R*-**1** (Figure S49). Because **1**–**3** all possess similar specific rotation values (**1**: −2.67; **2**: −3.33; **3**: −4.67), and contain the same relative configuration, each of the compounds are predicted to have the same absolute configuration. This yields absolute configurations 4*R*,8*S*,9*R* and 4*R*,6*R*,7*S*,8*R* for compounds **2** and **3**, respectively. Compound **4** exhibited a positive specific rotation value, so we used a different approach to assign its absolute configuration. A small aliquot of compound **4** was hydrolyzed with sodium hydroxide and the resulting product was subsequently treated with 4-bromobenzoyl chloride and 4-dimethylaminopyridine to yield the dibromobenzoyl product (**9**) (Figure 3B). The purified derivative exhibited a positive split Cotton effect, indicating an absolute configuration of 4*S*,6*R*,7*S*,8*S*,9*S*-**4**. This was further confirmed through TD-DFT predictions of ECD data for **4**, which also displayed the positive Cotton effect (Figure 3C).

**Figure 4:**
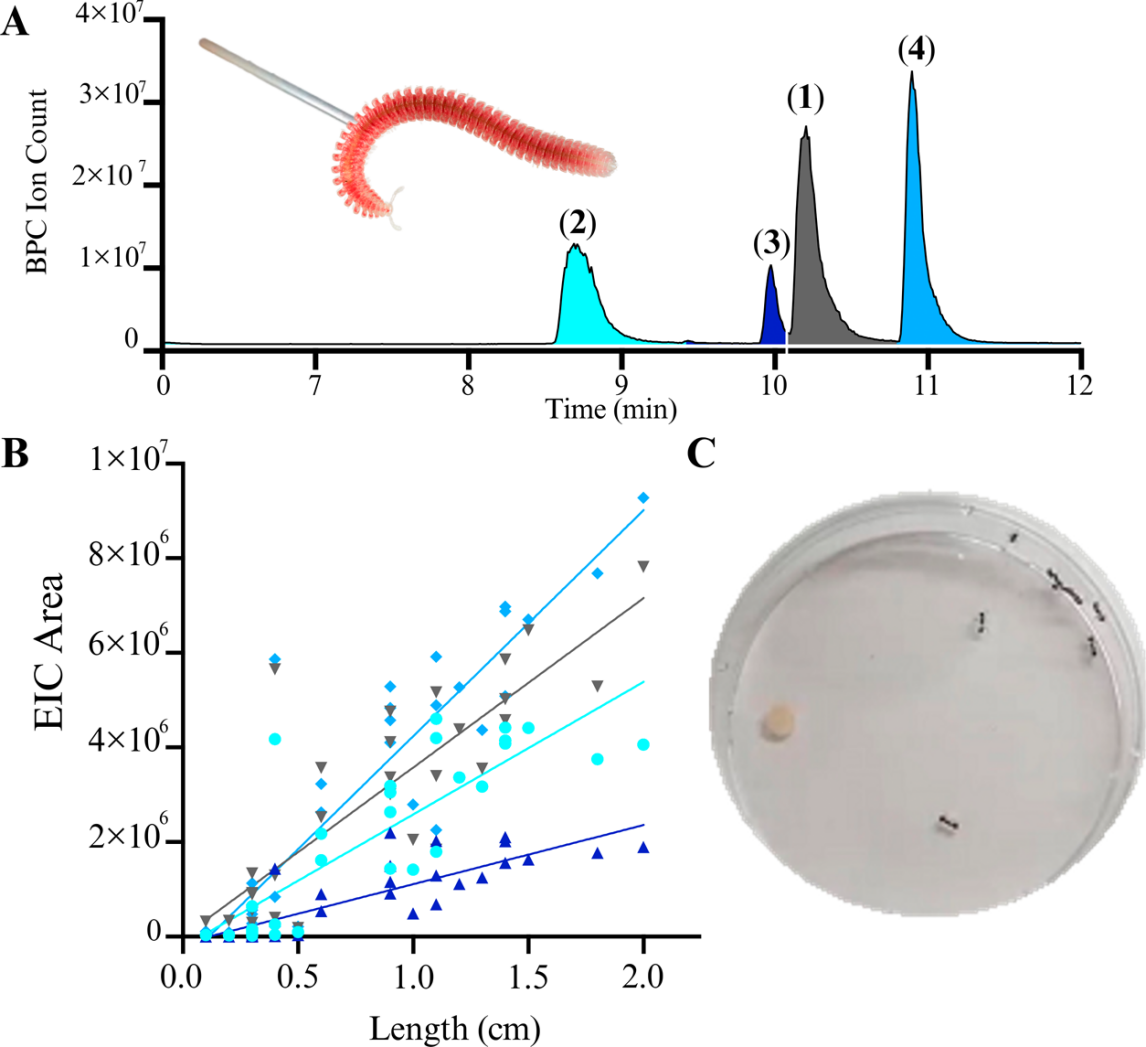
(A) The base peak chromatogram (BPC) of *I. plicata* secretions that were captured by the capillary. All four major peaks corresponded to the alkaloids (**1**–**4**), (B) The relative abundance of each alkaloid correlates to the length of the millipede (**1** – R^2^ = 0.6991; **2** – R^2^ = 0.6790; **3** – R^2^ = 0.6737; **4** – R^2^ = 0.7943), and (C) Predator assay. Paper disc impregnated with alkaloids (**1**–**4**) deterred the ants which placed themselves in the opposite direction; Two ants demonstrated motor disorder, leading to paralysis in close contact with the paper disc.

Based on isolated quantities from whole-animal extracts of *I. plicata,* the ischnocybines are produced in significant quantities, with each individual millipede estimated to contain approximately 50 μg (Table S16). This suggests these metabolites play an integral ecological role for the millipedes. Previous work has shown that other millipede derived alkaloids, including polyzonimine and **5**, along with the monoterpenes serve to protect the host against predation by ants and spiders, likely natural predators of millipedes.^4,8,9^ To ascertain whether defensive secretions of *I. plicata* contained the ischnocybines, the secretions were captured using capillaries and analyzed via LCMS. High abundances of **1**–**4** were detected with almost no other metabolites observed (Figure 4A). In addition, during the Oct. 2023 collection trip, individual millipedes were collected, their body lengths measured, and the secretions extracted and quantified by LCMS. All extracts contained **1**–**4**, even the juveniles which were less than 4 mm in length (Table S17). Peak areas of **1**–**4** plotted against millipede length, revealed a linear relationship between size and abundance of the alkaloids (Figure 4B). Finally, **1**–**4** were evaluated against *Aphaenogaster rudis-picea* complex (winnow ants), an ant local to southwestern Virginia, to determine if these alkaloids affected the ant’s behavior (Figure 4C). Briefly, approximately ten ants were placed in a Petri dish and allowed to settle (∼5 min). A disc with methanol was subsequently placed in the middle of the dish. The ants had no visible reaction to the methanol. Next, a disc impregnated with the ischnocybines was placed in the Petri dish and the ant’s behavior was monitored for 20 min. During this time, the ants clearly avoided the impregnated disc, staying on the opposite side of the Petri dish. Interestingly, two of the ants that approached the impregnated disc stopped moving for several minutes and then appeared to preen their antennae, suggesting a potential neurological target of the alkaloids.

**Figure 5:**
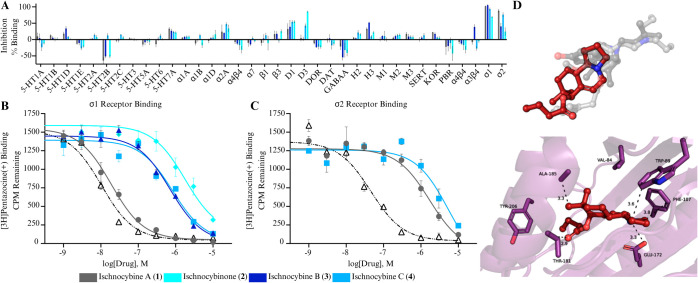
(A) Representative data from the primary binding assay inhibition. A total of 53 neuroreceptors were screened and secondary screens were conducted for any primary inhibition >50%. (B) Competitive binding curves for **1**–**4**, against the radioactive σ_1_R ligand, [3H]-(+)-pentazocine. (C) Competitive binding curves for **1**–**4**, against the radioactive σ_2_R ligand, [3H]-(+)pentazocine. Positive control was haloperidol for both σ_1_R and σ_2_R (open triangles). (D) Ischnocybine A (**1**) docked into the agonist σ_1_R. Compound **1**, shown as red sticks (colored by element). (i) Overlay of **1** (red) with the agonist (light gray) and antagonist binding positions (dark gray) binding positions from cocrystal structures. (ii) Interactions between residues in the σ_1_R (purple, colored by element) binding cavity and **1** (red, colored by element).

To probe this hypothesis, **1**–**4**, and **6** were screened in a range of neuroreceptor binding assays through the Psychoactive Drug Screening Program (PDSP) at UNC Chapel Hill. The ischnocybines (**1**–**4**) were first screened broadly at 10 μM against 53 neuroreceptors in a primary assay (Figures 5A and S50). Secondary radioligand binding assays to measure the inhibitor constant (K_i_) were conducted when the primary assay exhibited >50% binding affinity (Tables S18 and S19). Compounds **1**, **3**, and **4** were only found to have potent (K_i_ < 1 μM) and selective binding affinity for the σ_1_R (Table 1; Figure 4B). Ischnocybine A exhibited the highest affinity and selectivity for the σ_1_R with a K_i_ of 13.6 nM. Interestingly, **1** is 10-fold more active than both **3** (K_i_ 383.1 nM) and **4** (K_i_ 376.1 nM), and 100-fold more potent than **2** (K_i_ 1519.5 nM). Compounds **1**, **3**, and **4** all possessed nearly a 10-fold selectivity for σ_1_R over σ_2_R (Figure 4D and E). Surprisingly, simple addition of the ketone to C-6 in **2** leads to a nearly complete loss of activity, but the acetate (**3**) and butyrate (**4**) functionality are more tolerated. This suggests that steric hindrance only explains part of the reduced binding affinity. Compound **6** was screened after **1**–**4** and was only evaluated against σ_1_R over σ_2_R. Interestingly, **6** was over 150-fold less active than **1** (Figure S51)

**Table 1:** Inhibition Percent and K_i_ of σ_1_R and σ_2_R for **1**–**4**

Compound	Receptor	Inhibition %^a^	K_i_ (nM)
**1**	σ_1_	102 ± 2	14
	σ_2_	89 ± 9	1300
**2**	σ_1_	69 ± 4	1500
	σ_2_	25 ± 23	ND^b^
**3**	σ_1_	105 ± 4	380
	σ_2_	40 ± 19	ND^b^
**4**	σ_1_	93 ± 7	380
	σ_2_	76 ± 13	2900
**6**	σ_1_	62 ± 2	2200
	σ_2_	54 ± 13	3800

a At 10 μM.

b Not determined (primary screen yielded <50% binding).

It is not possible to determine if a ligand is an agonist or antagonist from *in vitro* binding affinity assays, and typically determining this is only possible through phenotypic changes in *in vivo* assays. However, known agonists and antagonists have different binding positions within the σ_1_R active site. The antagonist binding position falls almost entirely in the agonist position, with the major difference being a unique region by helix 4 which houses an interaction with Tyr-206 (Figure S52). Agonists are known to perturb helix 4 at Ala-185, causing it to shift further away (Figure S53) from the binding pocket. Using molecular docking, a redocking experiment was performed on the agonist (PDB ID: 6DK1),^13^ antagonist (PDB ID:6DK0),^13^ and apo structures (PDB ID: 7W2E)^22^ and indicated that molecular docking was able to predict accurate poses of both antagonist and agonist positions in the binding pocket (Figures S52–S54). The agonist structure was also able to recreate antagonistic binding poses of NE-100 accurately (RMSD value of 1.25 Å to cocrystal structure positioning) and with more poses, so the agonist structure was selected for further experiments with the alkaloid compounds (Figure S54)

Ischnocybines that docked into the σ_1_R agonist structure consistently occupy the unique region in the left quadrant of the binding pocket for the most potent compound and maintain interactions with Tyr-206 like known antagonists. This suggests that this compound set may be acting as antagonists. Docking studies and predicted free energy of binding MM/GBSA analysis with **1** regardless of stereoisomer type had the most favorable predicted free binding energy and ligand efficiency in the agonist σ_1_R, as well as participated in the most consistent and close interactions with Tyr-206 (Figure S55; Table S20). Compounds **3** and **4** participated in more distal interactions with Tyr-206 and had very similar predicted free energy of binding (Figures S56 and S57), but to a lesser degree than **1**. Compound **2** had the least favorable positioning in the binding pocket and was unable to interact with Tyr-206 (Figure S58) to the same degree as **1**, **3**, and **4**, suggesting a lower affinity for the σ_1_R that agrees with experimental results. Although σ_1_R is an orphan receptor, antagonists of σ_1_R have been implicated for the treatment of neuropathic pain, such as MR-309, which is in clinical trials for the treatment of neuropathic pain.^16^

In summary, ischnocybines are new highly modified heterocyclic terpenoid alkaloids produced by *I. plicata* and these compounds are the most complex millipede natural products yet to be discovered. The ischnocybines contain a 5,6,6-fused tricyclic system with five contiguous stereogenic centers, and are the first alkaloids in this class to have absolute configuration determined. Ischnocybines A, B, and C each exhibited potent and specific binding affinity for the σ_1_R over all other neuroreceptors and this may provide insights into the biomedical target underpinning the observed defensive properties. This study lays the foundation for other colobognath millipedes to gain a better understanding of the evolution of this intriguing pathway and the true biomedical application of this family of metabolites.

## Experimental Section

### General Experimental Procedures

Optical rotations were measured on a JASCO P-2000 polarimeter, circular dichroism spectrum on a JASCO J-815, and UV spectra on a Hitachi U4100 UV/Visible/Near-infrared spectrophotometer. All NMR data was acquired on a Bruker Neo NMR spectrometer operating at a ^1^H observation frequency of 499.861999 MHz and equipped with a H/F C/N TCI 5 mm Prodigy CryoProbe using TopSpin (version 4.1.4). Samples were prepared in 3 mm NMR tubes with 150 μL solvent. All data were acquired at 25 °C in DMSO-*d*_6_ (99.96%, Cambridge Isotope Laboratories). All NMR data were processed in MestReNova (version 15.0.0). ^1^H NMR data was referenced to 2.50 ppm for DMSO-*d*_6_, and absolute referencing was used for all other NMR spectra. HMBC experiments were optimized for ^n^*J*_CH_ = 8 Hz, and ROESY experiments were performed with a mixing time of 300 ms. GCMS was carried out in the EI mode using a Shimadzu QP-2020 GC-MS equipped with an RTX-5, 30 m × 0.25 mm i.d. column. The instrument was programmed from 60 to 250 °C at 10 °C/min. LR-LCMS data were obtained using an Agilent 1200 series HPLC system equipped with a photodiode array detector and a Thermo LTQ mass spectrometer. HRESIMS was carried out using a Shimadzu LC-q-TOF Mass Spectrometer equipped with an uHPLC system. HPLC purifications were carried out using Agilent 1200 series or 1260 Infinity II HPLC systems (Agilent Technologies) equipped with a photodiode array detector. All solvents were of HPLC quality.

### Millipede Collections

Millipedes were collected in southwest Oregon by opportunistic searches of down woody debris in coniferous and mixed hardwood forests. Under the assumption that larger individuals produce larger quantities of defensive chemicals, this study focused on adult individuals to maximize chemical collection: in *Brachycybe lecontii*, ozopore secretions are not exuded until stadium III.^23^ Thus, when aggregations were discovered, typically only the largest individuals were collected, while unpigmented larvae and relatively small individuals (under ∼8–10 mm in length) were left in situ. Collected millipedes were identified as *I. plicata* by analysis of morphological features and preserved in 100% methanol (MeOH) at −20 °C. Collections of *I. plicata* took place in (1) Jackson Co., Oregon, Rogue River-Siskiyou NF, 42.131960°N, −122.814510°W, Elev. 1153 m, 1 December 2020; (2) Jackson Co., Oregon, Applegate Lake, Collings Mtn. Trail, 42.052980°N, −123.133900°W, Elev. 654 m, 6 December 2020; (3) Jackson, Co., Oregon, East Applegate Ridge Trail, 42.245830°N, −122.982520°W, Elev. 944 m, 9 December 2020; (4) Jackson, Co., Oregon, East Applegate Ridge Trail, 42.245740°N, −122.982450°W, Elev. 944 m, 5 January 2021; (5) Jackson Co., Oregon, East Applegate Ridge Trail, 42.245890°N, −122.982740°W, Elev. 938 m, 1 October, 2023; (6) Jackson Co., Oregon, Sterling Mine Ditch Trail, 42.166010 ^o^N, −122.915980°W, Elev. 863 m, 3 October, 2023.

### Gas Chromatography Mass Spectrometry (GCMS) Analysis of Crude Mixture

Initial GCMS examination of the methanol extracts of *I. plicata* showed the presence of α- and β-pinene and a trace of terpinolene, along with four longer retention time alkaloids: (**1**), M = 291, (**2**), M = 305, (**3**), M = 349, and (**4**) M = 379. A slow stream of hydrogen was bubbled through 0.1 mL of the original extract in the presence of a few mg of PtO_2_ for 2 min. GCMS analysis of the resulting mixture showed that compounds **1**–**3** each added one H_2_ (M = 293, 307, and 351, respectively). The mass spectra of all four alkaloids showed a distinctive M-87 fragment suggesting a butyl ester. The presence of butyl esters rather than isobutyl esters was confirmed by the formation of *n*-butyl butanamide upon warming 20 μL of the extract with a few drops of *n*-butylamine. Next, a small portion of the original extract was warmed with a few mg of methoxylamine HCl and 2 drops of pyridine for 18 h. GCMS analysis of the resulting mixture showed that **2** formed a methoxime (M = 334) indicating the presence of a ketone in **2**. Finally, methanolysis was carried out by treating ca. 200 μL of the methanolic extract with ca. 1–2 mg of anhydrous NaOMe at rt overnight. GCMS analysis revealed the presence of a butyrate on **1** and **2**, a butyrate and acetate on **3**, and two butyrates on **4**.

### Purification of Ischnocybine A–C and Ischnocybinone (**1**–**4**)

HPLC purifications were carried out using an Agilent 1200 series HPLC system equipped with a 1260 Infinity II collector (Agilent Technologies) and photodiode array detector. Purification of compounds **1**–**4** was performed reversed-phase C_18_ semipreparative column (Phenomenex Synergi, 10.0 × 250 mm, 4 μm) using the following solvent gradient system: holding 30% acetonitrile (MeCN) + 0.1% formic acid (FA)/70% water +0.1% FA then gradient to 100% MeCN + 0.1% FA over 30 min with a 3 mL/min flow rate. An aliquot (40 μL) of the crude extract was resuspended at 10 mg/mL in 50% MeCN/water while monitoring at 195 and 210 nm. The compounds eluted at 13.3 (**1**), 6.2 (**2**), 11.9 (**3**), and 17.4 (**4**) min, yielding 3.5, 4.6, 2.5, and 4.1 mg, respectively, from 300 individual millipedes. The October 2023 collection followed a similar purification, except the solvent gradient was holding 20% MeCN + 0.1% FA/80% water +0.1% FA for 5 min then gradient to 55% MeCN + 0.1% FA/45% water +0.1% FA over 25 min, followed by a hold at 100% MeCN + 0.1% FA for 5 min finally a hold of 20% MeCN + 0.1% FA/80% water +0.1% FA for 5 min. The compounds eluted at 22.7 (**1**) 14.8 (**2**), 20.6 (**3**), 27.6 (**4**) and 8.2 (**6**).

### Ischnocybine A (**1**)

Amorphous solid; [α]^23^_D_ −2.67 (*c* 0.015, MeOH); UV (MeOH) λ_max_ (log ε) 2.90 (190) nm; NMR (500 MHz, *d*_6_*-*DMSO) δ 5.65 (dd, *J* = 4.4 and 2.3 Hz, 1H, H-2), 4.88 (d, *J* = 1.4, 1H, H-4), 3.30 (ddd, *J* = 16.3, 5.0, 2.3, CH, H-1a), 3.00 (m, CH, H-12a), 2.59 (ddd, *J* = 16.3, 4.4, and 1.7, CH, H-1b), 2.24 (td, *J* = 7.3 and 1.3, CH_2_, H-14), 1.97 (m, 3CH, 2CH_2_, H-10a, H-12b) 1.97 (m, CH, H-8), 1.73 (td, *J* = 9.0 and 6.8 Hz, CH, H-9), 1.65 (m, 4CH, H-6a, H7a, H11), 1.53 (qd, *J* = 7.3 and 2.8 Hz, CH_2_, H-15), 1.32 (m, CH, H-10b), 1.19 (m, CH, H-6b), 1.14 (m, CH, H-7b), 0.87 (t, *J* = 7.3, CH_3_, H-16), 0.84 (s, CH_3_, H-18), 0.81 (s, CH_3_, H-17); ^13^C NMR (100 MHz, *d*_6_*-*DMSO) δ 172.2 (C, C-13), 135.4 (C, C-3), 126.3 (CH, C-2), 80.1 (CH, C-4), 67.0 (CH, C-9), 54.2 (CH_2_, C-12), 52.1 (CH_2_, C-1), 39.4 (CH, C-8), 36.3 (CH_2_, C-14), 34.5 (C, C-5), 32.4 (CH_2_, C-6), 29.4 (CH_2_, C-10), 27.2 (CH_3_, C-18), 25.8 (CH_2_, C-7), 23.8 (CH_3_, C-17), 21.6 (CH_2_, C-11), 18.6 (CH_2_, C-15), 13.9 (CH_3_, C-16); EIMS *m*/*z* 291 [M^+^] (31), 204 (41), 203 (28), 188 (63), 148 (6), 135 (5), 134 (100), 119 (81), 71 (16), 70 (63); HRESIMS [M + H]^+^*m*/*z* 292.2277 (calcd for C_18_H_30_NO_2_ 292.2277, Δ 0.0 ppm).

### Ischnocybinone (**2**)

Amorphous solid; [α]^23^_D_ −3.33 (*c* 0.018, MeOH); UV (MeOH) λ_max_ (log ε) 6.4 (190) nm; NMR [500 MHz, *d*_6_*-*DMSO] δ 5.92 (dd, *J* = 4.9 and 2.1 Hz, CH, H-2), 5.30 (s, CH, H-4), 3.41 (ddd, *J* = 16.8, 4.9, 2.1 Hz, CH, H-1a), 3.03 (ddd, *J* = 8.8, 6.7, 3.6, CH, H-12a), 2.74 (ddd, *J* = 16.8, 4.0, 1.7, CH, H-1b), 2.47 (m, CH, H-8), 2.40 (d, *J* = 11.4, CH, H-7a), 2.31 (m, CH, H-7b), 2.21 (t, *J* = 7.4, CH_2_, H-14), 2.06 (q, *J* = 8.8, CH, H-12b), 1.98 (m, CH, H-9), 1.93 (m, CH, H-10a), 1.69 (m, CH_2_, H-11), 1.48 (dtd, *J* = 14.8, 7.4, and 5.9, CH_2_, H-15), 1.33 (m, CH, H-10b), 1.08 (s, CH_3_, H-17), 0.92 (s, CH_3_, H-18), 0.84 (t, *J* = 7.4, CH_3_, H-16) ; ^13^C NMR [100 MHz, *d*_6_*-*DMSO] δ 211.7 (C, C-6), 171.9 (C, C-13), 132.3 (C, C-3), 128.1 (CH, C-2), 83.6 (CH, C-4), 66.9 (CH, C-9), 53.9 (CH_2_, C-12), 51.9 (CH_2_, C-1), 48.7 (C, C-5), 40.3 (CH, C-8), 40.1 (CH_2_, C-7), 36.0 (CH_2_, C-14), 29.0 (CH_2_, C-10), 24.2 (CH_3_, C-17), 21.5 (CH_2_, C-11), 20.0 (CH_3_, C-18), 18.4 (CH_2_, C-15), 13.8 (CH_3_, C-16); EIMS *m*/*z* 305 [M^+^] (56), 218 (38), 217 (13), 202 (28), 174 (9), 148 (28), 133 (15), 71 (47), 70 (100), 43 (15); HRESIMS [M + H]^+^*m*/*z* 306.2072 (calcd for C_18_H_28_NO_3_ 306.2069, Δ 0.9 ppm).

### Ischnocybine B (**3**)

Amorphous solid; [α]^23^_D_ −4.67 (*c* 0.015, MeOH); UV (MeOH) λ_max_ (log ε) 1.9 (190) nm; NMR [500 MHz, *d*_6_*-*DMSO] δ 5.74 (m, CH, H-2), 5.04 (s, CH, H-4), 5.01 (dd, *J* = 12.1 and 4.9, CH, H-6), 3.31 (m, CH, H-1a), 3.00 (m, CH, H-12a), 2.63 (m, CH, H-1b), 2.28 (td, *J* = 7.4, and 2.1, CH_2_, H-14), 2.19 (m, CH, H-8), 2.12 (m, CH, H-12b), 2.02 (s, CH_3_, H-18), 1.94 (m, CH, H-10a), 1.81 (m, CH, H-7a), 1.81 (m, CH, H-9) 1.67 (m, CH_2_, H-11), 1.53 (m, CH_2_, H-15), 1.33 (m, CH, H-10b), 1.20 (m, CH, H-7b), 0.87 (t, *J* = 7.4, CH_3_, H-16), 0.84 (s, 2CH_3_, H-19, H-20); ^13^C NMR [100 MHz, *d*_6_*-*DMSO] δ 172.0 (C, C-13), 170.6 (C, C-17), 133.3 (C, C-3), 127.6 (CH, C-2), 81.4 (CH, C-4), 73.6 (CH, C-6), 66.5 (CH, C-9), 54.1 (CH_2_, C-12), 52.0 (CH_2_, C-1), 38.9 (CH, C-8), 38.5 (C, C-5), 36.2 (CH_2_, C-14), 30.7 (CH_2_, C-7), 29.2 (CH_2_, C-10), 23.0 (CH_3_, C-20), 21.6 (CH_2_, C-11), 21.3 (CH_3_, C-18), 18.6 (CH_3_, C-19), 18.6 (CH_2_, C-15), 13.9 (CH_3_, C-16); EIMS *m*/*z* 349 [M^+^] (23), 262 (47), 261 (30), 248 (40), 202 (7), 186 (23), 150 (43), 135 (13), 133 (16), 132 (100), 71 (20), 70 (63), 43 (16); HRESIMS [M + H]^+^*m*/*z* 350.2335 (calcd for C_20_H_32_NO_4_ 350.2331, Δ 1.1 ppm).

### Ischnocybine C (**4**)

Amorphous solid; [α]^23^_D_ +6.09 (*c* 0.023, MeOH); UV (MeOH) λ_max_ (log ε) 1.6 (190) nm; NMR [500 MHz, *d*_6_*-*DMSO] δ 5.41 (dd, *J* = 11.6 and 2.7, CH, H-7), 4.87 (d, *J* = 2.7, CH, H-6), 3.00 (m, CH, H-1a), 2.91 (td, *J* = 8.5 and 2.5, CH, H-12a), 2.30 (td, *J* = 7.3 and 1.5, CH_2_, H-14), 2.25 (m, CH, H-8), 2.09 (td, *J* = 7.4 and 2.9, CH_2_, H-18), 2.04 (m, CH, H-9), 1.90 (d, *J* = 8.5, CH, H-12b), 1.86 (m, CH, H-3), 1.86 (m, CH, H-2a), 1.81 (m, CH, H-1b), 1.77 (m, CH, H-10a), 1.70 (dd, *J* = 14.2 and 4.9, CH, H-4a), 1.63 (ddd, *J* = 13.7, 9.2, and 5.3, CH, H-10b), 1.57 (qd, *J* = 7.3 and 3.6, CH_2_, H-15), 1.52 (m, CH, H-11a), 1.48 (q, *J* = 7.4, CH_2_, H-19), 1.44 (m, CH, H-11b), 1.35 (m, CH, H-2b), 1.26 (d, *J* = 14.2, CH, H-4b), 1.10 (s, CH_3_, H-22), 0.91 (t, *J* = 7.3, CH_3_, H-16), 0.84 (t, *J* = 7.4, CH_3_, H-20), 0.77 (s, CH_3_, H-21); ^13^C NMR [100 MHz, *d*_6_*-*DMSO] δ 172.9 (C, C-13), 172.4 (C, C-17), 76.5 (CH, C-6), 69.3 (CH, C-7), 66.9 (CH, C-9), 54.3 (CH_2_, C-12), 54.1 (CH_2_, C-1), 39.0 (CH_2_, C-4), 36.7 (CH, C-3), 36.2 (CH_2_, C-18), 36.1 (CH_2_, C-14), 35.8 (CH, C-8), 35.0 (C, C-5), 29.3 (CH_2_, C-2), 29.3 (CH_3_, C-2), 27.9 (CH_3_, C-22), 27.7 (CH_2_, C-10), 21.7 (CH_2_, C-11), 18.6 (CH_2_, C-15), 17.9 (CH_2_, C-19), 13.9 (CH_3_, C-20), 13.8 (CH_3_, C-16); EIMS *m*/*z* 379 [M^+^] (20), 378 (13), 293 (17), 292 (100), 222 (7), 204 (53), 97 (7), 96 (23), 84 (90), 83 (22), 43 (12); HRESIMS [M + H]^+^*m*/*z* 380.2794 (calcd for C_22_H_37_NO_4_ 380.2801, Δ 1.8 ppm).

### Ischnocybine A Alcohol (**6**)

Amorphous solid; [α]^23^_D_ +2.98 (*c* 0.019, MeOH); NMR [600 MHz, *d*_6_*-*DMSO] δ 5.46 (m, CH, H-2), 3.41 (m, CH, H-4), 3.39 (m, CH, H-1a), 3.13 (m, CH, H-12b), 2.82 (m, CH, H-1b), 2.22 (m, CH, H-8), 2.21 (m, CH, H-12a), 2.01 (m, CH, H-9), 2.01 (m, CH, H-10b), 1.73 (m, CH_2_, H-11), 1.71 (m, CH, H-6b), 1.56 (m, CH, H-7b), 1.37 (m, CH, H-10a), 1.07 (m, CH, H-7a), 1.03 (m, CH, H-6a), 0.89 (s, CH_3_, H-13), 0.70 (s, CH_3_, H-14); ^13^C NMR [125 MHz, *d*_6_*-*DMSO] δ 140.5 (C, C-3), 119.8 (CH, C-2), 77.8 (CH, C-4), 66.4 (CH, C-9), 53.1 (CH_2_, C-12), 50.8 (CH_2_, C-1), 37.2 (CH, C-8), 34.7 (C, C-5), 30.9 (CH_2_, C-6), 28.4 (CH_2_, C-10), 27.2 (CH_3_, C-13), 25.7 (CH_2_, C-7), 23.8 (CH_3_, C-14), 20.8 (CH_2_, C-11); HRESIMS [M + H]^+^*m*/*z* 222.1856 (calcd for C_14_H_23_NO 222.1852, Δ 1.25 ppm).

### DFT and TD-DFT Results for All Configurations of Ischnocybine Compounds (**1**–**4**)

For NMR δ prediction analysis of **1**–**4**, to reduce computational overhead, alkyl chains from butyrate groups were truncated to yield acetate groups for quantum mechanical calculations. Structures were MMFF94 energy-minimized in Chem3D and extended mixed torsional/low-mode (MTLMOD) conformational searches were performed using the OPLS4^24^ force field in the Schrödinger MacroModel software package. NMR predictions were based on the DELTA50^20^ methodology; ^13^C δ predictions were performed at the PCM-ωB97X-D/def2-SVP//PCM-B3LYP-D3/6-311G(d,p) level of theory, and ^1^H δ predictions were performed at the PCM-WP04/6-311++G(2d,p)//PCM-B3LYP-D3/6-311G(d,p) level of theory, both of which utilized an integral equation formalism (IEF) polarizable continuum model (PCM) for DMSO. Linear scaling factors include a slope of −1.0099 and intercept of 196.0386 for ^13^C, and a slope of −1.0080 and intercept of 32.0998 for ^1^H. Predicted chemical shifts were Boltzmann-weighted based on the total Gibbs free energy of each conformer. No imaginary frequencies were present within the frequency calculation results. Relative mean standard deviation (RMSD) and mean absolute error (MAE) of chemical shifts were compared between all possible configurations for **1**–**4**.

For ECD analysis of **4**, time-dependent (TD) DFT calculations were carried out for the prediction of frequency data at the PCM-ωB97X-D/6-311++G(2d,p)//B3LYP-D3/6-311G level of theory for n = 100 excited states, with an IEFPCM for MeOH. Transition energies, oscillator strengths, and rotatory strengths were extracted from the resulting Gaussian output files. Then UV–vis and ECD spectra were generated with the following equation:
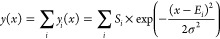


Above, “y_i_(x)” is the contribution to the spectrum from the i-th transition, “S_i_” is the strength of the i-th transition (oscillator strength for UV–vis, rotatory strength for ECD), “E_i_” is the energy of the i-th transition, “σ” is the broadening factor (optimized based on experimental data band widths), and “x” is the energy variable over which the spectrum is calculated. Respective energies (derived in units of eV) were converted to wavelengths (nm) through the relationship: wavelength (nm) × energy (eV) = 1240. Data for all conformers were Boltzmann-weighted based on the total Gibbs free energies, and relative intensities were scaled to match the experimental data for superimposition. Wavelength corrections were applied based on experimental UV–vis data.

### Prey Deterrent Assays

To test the repellent effect of **1**–**4** on a likely predator, approximately 10 *Aphaenogaster* sp. ants were placed on Petri plates (100 mm × 15 mm) with a paper disc containing 100 μg of **1**–**4** dissolved in methanol. First, each compound was tested separately and then combined on a single paper disc. A paper disc impregnated with only methanol was used as a negative control. The ants’ behaviors were monitored for 5 to 15 min.^25^

### Relative Quantification of Ischnocybines (**1**–**4**) on Individual Millipedes

To determine whether the size of the millipede impacts how much of the alkaloids are produced, we collected 29 individual *I. plicata* in 1.5 mL Eppendorf tubes. Each of the millipedes were measured with a ruler and then preserved in 0.5 mL of MeOH. A small aliquot was taken from each millipede and run on LR-LCMS (LTQ) using a Kinetex 5 μm EVO C18 100 × 3.0 mm column and the following solvent gradient system: holding 10% MeCN + 0.1% FA/90% water +0.1% FA for 3 min followed by a gradient to 100% MeCN + 0.1% FA over 11 min, holding for 3 min. The. raw files were converted to mzML using MSConvert (Version 3.0.20337). The data was processed in MzMine3 (Version 3.2.8). Mass detections were conducted with a noise threshold of 2E4. The ADAP chromatogram builder used a minimum group size of 5, a group intensity threshold of 2E4, minimum height intensity of 2E4 and *m*/*z* tolerance was 0.5. The local minimum resolver utilized the following settings: Chromatographic threshold: 95%, Search minimum in RT range (min) = 0.2, minimum relative height = 1%, minimum absolute height = 1E2 minimum ratio or peak top/edge = 1.4, Minimum number of data points = 3 and peak duration range (min) = 0.00–1. The 13C isotope filler had an *m*/*z* tolerance of 0.5 *m*/*z*, retention time tolerance of 0.3, maximum charge of 3 and representative isotope of most intense. Finally, the join aligner had an *m*/*z* tolerance of 0.5 *m*/*z*, a weight for *m*/*z* of 75, retention time tolerance of 0.3 min, and weight for RT of 25. The. csv file was then exported, the peak areas for extracted ion chromatograms corresponding to the [M + H]^+^ for each ischnocybine [292 (**1**), 206 (**2**), 350 (**3**), and 380 (**4**)], were plotted against the length of each millipede using Prism (Version 10.2.0).

### Mosher Esterification of Ischnocybine A Alcohol (**6**)

Mosher esterification reactions were performed following literature precedent.^21^ Briefly, two solutions of ischnocybine A alcohol (**6**) (1.25 mg, 5.66 μmol), two chunks (dimethylamino)pyridine (DMAP), and two drops triethylamine (TEA) in 150 μL of DCM. One was treated with (*R*)-(−)-α-Methoxy-α-(trifluoromethyl)phenylacetyl chloride (MTPA) chloride (7.5 μL, 40.1 μmol), while the other was treated with (*S*)-MTPA chloride (7.5 μL, 40.1 μmol). Both mixtures were left at rt overnight. Each reaction was extracted with 1-butanol (1 mL) and the organic layer was washed with brine (1 mL). The organic layer was dried and suspended in 50% MeCN/50% water and purified on HPLC using a C_18_ semipreparative column (Phenomenex Synergi, 10.0 mm × 250 mm, 4 μm) using the following solvent gradient system: holding 45% acetonitrile (MeCN) + 0.1% FA/55% water +0.1% FA for 5 min then gradient to 90% MeCN + 0.1% FA/10% water +0.1% FA over 10 min with a 3 mL/min flow rate, the product eluted at 8.2 min (**7** and **8**).

### Chemical Derivatization of Ischnocybine C (**4**) and Electronic Circular Dichroism Analysis

Ischnocybine C (1 mg) was treated with 1.5 mL 0.5N NaOH, heated to 40 °C and let stir for 2 days. The solution was quenched with 1.5 mL 0.5 N HCl and dried down, the product was carried through. The hydrolysis product was treated with bromobenzoyl chloride (6 mg), DMAP (2 chunks) and tetrahydrofuran (2 mL). The reaction was heated to 50 °C for 2 days. The reaction was extracted with ethyl acetate and the organic layer was washed with brine. The product was dried and resuspended in 50% MeCN/50% water then purified with HPLC using a C_18_ semipreparative column (Phenomenex Synergi, 10.0 mm × 250 mm, 4 μm) using the following solvent gradient system: holding 50% MeCN + 0.1% FA/50% water +0.1% FA for 5 min then gradient to 100% MeCN + 0.1% FA over 15 min with a 3 mL/min flow rate, the product eluted at 16.6 min.

### Binding Assay for the Sigma-1 and -2 Receptors

HEKT cell lines were used to make membrane pallets for binding assays. In the primary binding assays, compounds were tested at a single concentration (10 μM) and in quadruplicate in 96-well plates. Compounds showing a minimum of 50% inhibition at 10 μM were tagged for secondary radioligand binding assays to determine equilibrium binding affinity at specific targets. In secondary binding assays, compounds were tested at 11 concentrations (0.1, 0.3, 1, 3, 10, 30, 100, 300, 1,000, 3,000, and 10,000 nM) and in triplicate (3 sets of 96-well plates). Both primary and secondary radioligand binding assays were carried out in a final volume of 125 μL per well in appropriate binding buffers (50 mM Tris HCl, pH 8.0, rt). Pentazocine was used as a radioligand and the hot ligand concentration was determined close to its *K*_d_. Total binding and nonspecific binding were determined in the absence and presence of 10 μM of haloperidol, the reference compound. Plates were incubated at room temperature and in the dark for 90 min. Reactions were stopped by vacuum filtration onto 0.3% polyethylenimine (PEI) soaked 96-well filter mats using a 96-well Filtermate harvester, followed by three washes with cold wash buffers (50 mM Tris HCl, pH 7.4). Scintillation cocktail was then melted onto the microwave-dried filters on a hot plate and radioactivity was counted in a Microbeta counter. K_d_ values for [3H]-(+)-pentazocine (s1R: 6.70 nM), (s2R: 14.50) were determined via separate homologous competitive binding experiments. *K*_i_ values were determined from at least three independent experiments and are reported as mean ± SEM.

### Molecular Docking

To investigate the binding mechanism of action for alkaloid compounds to the σ_1_ receptor binding pocket, molecular docking was performed. Several σ_1_ receptor crystal structures were redocked with their original cocrystallized ligand and docked with our novel alkaloid compounds. The alkaloid compounds, ischnocybine A, B, C and ischnocybinone were prepared as pdb files by the Mevers lab. Each compound was prepared with *R* and *S* configuration, for a total of eight ligand files. Crystal structures representing agonistic (PDB ID: 6DK1),^26^ antagonistic (PDB ID: 6DK0),^13^ and apo (PDB ID: 7W2E)^22^ conformations of the σ_1_R were used to avoid potential bias in docking studies. The receptor files were prepared for docking in PyMOL 2.5.0^26^ where glycolipids, sulfates, glycerols, and waters were removed from the environment. Oligomeric structures were reduced to a single monomeric unit. The agonist receptor contained pentazocine and the antagonist receptor contained NE-100 as cocrystallized ligands.

The pentazocine, NE-100, and alkaloid compounds were redocked and docked using GNINA 1.0.3,^26^ a successor to AutoDock Vina^27^ that uses deep-learning scoring functions. AutoDock Tools 1.5.6^28^ was used to center the grid box onto the cocrystallized ligands in their respective structures, establishing x, y, z coordinates of 10.343, 38.597, and −34.851 (agonist, PDB ID: 6DK1), 16.549, 37.608, and −35.618 (antagonist, PDB ID: 6KD0), and −65.0, 3.0, and 80 (apo, PDB ID: 7W2E). A box size of 40 Åx40 Åx40 Å was utilized. Redocked root-mean-square deviation (RMSD) values for pentazocine in the agonist and NE-100 in the antagonist were as low as 0.74 and 1.25 Å respectively (Figure S39). Additionally, both cocrystallized ligands were docked into their counterpart structure and successfully mimicked their original binding position (Figures S37 and S38), further validating the protocol and the ability to predict if compounds sampled antagonistic or agonistic positioning in the binding pocket. The 8 alkaloid compounds ligands were docked into each of the 3 structures individually, generating 9 poses each. Poses found within the binding cavity were analyzed further. Analysis of the predicted binding poses was performed in PyMOL 2.5.0. Schrödinger Maestro 2021–2 was used to perform molecular mechanics with generalized Born and surface area solvation (MM/GBSA) energy calculations for each binding pose located in the binding pocket.^29^

## Data Availability

The original NMR data (FIDs) of compound **1**–**4** and **6** is available via NP-MRD (accession #: NP0333129, NP0333130, NP0333131, NP0350550, and NP0350551) at https://np-mrd.org/.

## Supporting Information Available

Detailed description of materials and methods, biological and ecological assays, mass spectrometry, NMR spectra, and computational XYZ coordinates (PDF)

## References

[ref1] Shear W. A. The Chemical Defenses of Millipedes (Diplopoda): Biochemistry, Physiology and Ecology. Biochem. Syst. Ecol. 2015, 61, 78–117. 10.1016/j.bse.2015.04.033.

[ref2] Shorter P. L.; Hennen D. A.; Marek P. E. Cryptic Diversity in Andrognathus Corticarius Cope, 1869 and Description of a New Andrognathus Species from New Mexico (Diplopoda, Platydesmida, Andrognathidae). Zookeys 2018, 786, 19–41. 10.3897/zookeys.786.27631. PMC6168611 30283233

[ref3] Eisner H. E.; Alsop D. W.; Eisner T. Defense Mechanisms of Arthropods. XX. Quantitative Assessment of Hydrogen Cyanide Production in Two Species of Millipedes. Psyche (Camb. Mass. 1967, 74 (2), 107–117. 10.1155/1967/861501.

[ref4] Gullan P. J.; Cranston P. S. Insects: An Outline of Entomology, 1994th ed.; Chapman and Hall: London, England, 1994.

[ref5] Stebbins R. C. Lizards Killed by a Millipede. Am. Midl. Nat. 1944, 32 (3), 777 10.2307/2421250.

[ref6] Banks P.; Funkhouser E. M.; Macias A. M.; Lovett B.; Meador S.; Hatch A.; Garraffo H. M.; Cartwright K. C.; Kasson M. T.; Marek P. E.; Jones T. H.; Mevers E. The Chemistry of the Defensive Secretions of Three Species of Millipedes in the Genus Brachycybe. J. Chem. Ecol. 2024, 50, 478 10.1007/s10886-024-01518-6. 38853234 PMC11493816

[ref7] Jones T. H.; Harrison D. P.; Menegatti C.; Mevers E.; Knott K.; Marek P.; Hennen D. A.; Kasson M. T.; Macias A. M.; Lovett B.; Saporito R. A. Deoxybuzonamine Isomers from the Millipede Brachycybe Lecontii (Platydesmida: Andrognathidae). J. Nat. Prod. 2022, 85 (4), 1134–1140. 10.1021/acs.jnatprod.2c00077. 35389651

[ref8] Wood W. F.; Hanke F. J.; Kubo I.; Carroll J. A.; Crews P. Buzonamine, a New Alkaloid from the Defensive Secretion of the Millipede, Buzonium Crassipes. Biochem. Syst. Ecol. 2000, 28 (4), 305–312. 10.1016/S0305-1978(99)00068-X. 10725589

[ref9] Smolanoff J.; Kluge A. F.; Meinwald J.; McPhail A.; Miller R. W.; Hicks K.; Eisner T. Polyzonimine: A Novel Terpenoid Insect Repellent Produced by a Milliped. Science 1975, 188 (4189), 734–736. 10.1126/science.1124395. 1124395

[ref10] Meinwald J.; Smolanoff J.; McPhail A. T.; Miller R. W.; Eisner T.; Hicks K. Nitropolyzonamine: A Spirocyclic Nitro Compound from the Defensive Glands of a Milliped (). Tetrahedron Lett. 1975, 16 (28), 2367–2370. 10.1016/0040-4039(75)80013-X.

[ref11] Kunert O.; Pferschy-Wenzig E. M.; Orthaber A.; Raspotnig G.; Bodner M. Alkaloids from Millipedes: A Re-Evaluation of Defensive Exudates from Polyzonium Germanicum. Front. Ecol. Evol. 2023, 11. 10.3389/fevo.2023.1212452.

[ref12] Hassler M. F.; Harrison D. P.; Jones T. H.; Richart C. H.; Saporito R. A. Gosodesmine, a 7-Substituted Hexahydroindolizine from the Millipede Gosodesmus Claremontus. J. Nat. Prod. 2020, 83 (9), 2764–2768. 10.1021/acs.jnatprod.0c00722. 32915571

[ref13] Schmidt H. R.; Betz R. M.; Dror R. O.; Kruse A. C. Structural Basis for ∑1 Receptor Ligand Recognition. Nat. Struct. Mol. Biol. 2018, 25 (10), 981–987. 10.1038/s41594-018-0137-2. 30291362 PMC6261271

[ref14] Maurice T.; Su T.-P. The Pharmacology of Sigma-1 Receptors. Pharmacol. Ther. 2009, 124 (2), 195–206. 10.1016/j.pharmthera.2009.07.001. 19619582 PMC2785038

[ref15] Ryskamp D. A.; Korban S.; Zhemkov V.; Kraskovskaya N.; Bezprozvanny I. Neuronal Sigma-1 Receptors: Signaling Functions and Protective Roles in Neurodegenerative Diseases. Front. Neurosci. 2019, 13, 1212452 10.3389/fnins.2019.00862. PMC6736580 31551669

[ref16] Bruna J.; Videla S.; Argyriou A. A.; Velasco R.; Villoria J.; Santos C.; Nadal C.; Cavaletti G.; Alberti P.; Briani C.; Kalofonos H. P.; Cortinovis D.; Sust M.; Vaqué A.; Klein T.; Plata-Salamán C. Efficacy of a Novel Sigma-1 Receptor Antagonist for Oxaliplatin-Induced Neuropathy: A Randomized, Double-Blind, Placebo-Controlled Phase IIa Clinical Trial. Neurotherapeutics 2018, 15 (1), 178–189. 10.1007/s13311-017-0572-5. 28924870 PMC5794691

[ref17] Brewer M. S.; Spruill C. L.; Rao N. S.; Bond J. E. Phylogenetics of the Millipede Genus Brachycybe Wood, 1864 (Diplopoda: Platydesmida: Andrognathidae): Patterns of Deep Evolutionary History and Recent Speciation. Mol. Phylogenet. Evol. 2012, 64 (1), 232–242. 10.1016/j.ympev.2012.04.003. 22516430

[ref18] Marek P. E.; Buzatto B. A.; Shear W. A.; Means J. C.; Black D. G.; Harvey M. S.; Rodriguez J. The First True Millipede-1306 Legs Long. Sci. Rep. 2021, 11 (1), 23126 10.1038/s41598-021-02447-0. 34916527 PMC8677783

[ref19] Benavides L. R.; Edgecombe G. D.; Giribet G. Re-Evaluating and Dating Myriapod Diversification with Phylotranscriptomics under a Regime of Dense Taxon Sampling. Mol. Phylogenet. Evol. 2023, 178, 107621 10.1016/j.ympev.2022.107621. 36116731

[ref20] Cohen R. D.; Wood J. S.; Lam Y.-H.; Buevich A. V.; Sherer E. C.; Reibarkh M.; Williamson R. T.; Martin G. E. DELTA50: A Highly Accurate Database of Experimental 1H and 13C NMR Chemical Shifts Applied to DFT Benchmarking. Molecules 2023, 28 (6), 2449 10.3390/molecules28062449. 36985422 PMC10051451

[ref21] Ohtani I.; Kusumi T.; Kashman Y.; Kakisawa H. High-Field FT NMR Application of Mosher’s Method. The Absolute Configurations of Marine Terpenoids. J. Am. Chem. Soc. 1991, 113 (11), 4092–4096. 10.1021/ja00011a006.

[ref22] Meng F.; Xiao Y.; Ji Y.; Sun Z.; Zhou X. An Open-like Conformation of the Sigma-1 Receptor Reveals Its Ligand Entry Pathway. Nat. Commun. 2022, 13 (1), 1267 10.1038/s41467-022-28946-w. 35273182 PMC8913746

[ref23] Wong V.; Hennen D.; Macias A.; Brewer M.; Kasson M.; Marek P. Natural history of the social millipede *Brachycybe lecontii* Wood, 1864. BDJ 2020, 8, e50770 10.3897/BDJ.8.e50770. 32296285 PMC7148388

[ref24] Lu C.; Wu C.; Ghoreishi D.; Chen W.; Wang L.; Damm W.; Ross G. A.; Dahlgren M. K.; Russell E.; Von Bargen C. D.; Abel R.; Friesner R. A.; Harder E. D. OPLS4: Improving Force Field Accuracy on Challenging Regimes of Chemical Space. J. Chem. Theory Comput. 2021, 17 (7), 4291–4300. 10.1021/acs.jctc.1c00302. 34096718

[ref25] Estrada C.; Wcislo W. T.; Van Bael S. A. Symbiotic Fungi Alter Plant Chemistry That Discourages Leaf-cutting Ants. New Phytol. 2013, 198 (1), 241–251. 10.1111/nph.12140. 23406415

[ref26] McNutt A. T.; Francoeur P.; Aggarwal R.; Masuda T.; Meli R.; Ragoza M.; Sunseri J.; Koes D. R. GNINA 1.0: Molecular Docking with Deep Learning. J. Cheminform. 2021, 13 (1), 43 10.1186/s13321-021-00522-2. 34108002 PMC8191141

[ref27] Trott O.; Olson A. J. AutoDock Vina: Improving the Speed and Accuracy of Docking with a New Scoring Function, Efficient Optimization, and Multithreading. J. Comput. Chem. 2010, 31 (2), 455–461. 10.1002/jcc.21334. 19499576 PMC3041641

[ref28] Morris G. M.; Huey R.; Lindstrom W.; Sanner M. F.; Belew R. K.; Goodsell D. S.; Olson A. J. AutoDock4 and AutoDockTools4: Automated Docking with Selective Receptor Flexibility. J. Comput. Chem. 2009, 30 (16), 2785–2791. 10.1002/jcc.21256. 19399780 PMC2760638

[ref29] Maestro, version 9.4. Schrödinger, LLC, New York, NY; 2013.

