# Supplementary material for: Neuromodulating Alkaloids from Millipede Defensive Secretions
Source: J Nat Prod. 2024 Dec 30;88(1):110–8. doi: 10.1021/acs.jnatprod.4c01162 (PMC11773556; doi:10.1021/acs.jnatprod.4c01162)
Supplement: Supplementary file 1 — np4c01162_si_001.pdf [file np4c01162_si_001.pdf]

Supporting Information

Neuromodulating Alkaloids from Millipede Defensive Secretions

*Carla Menegatti,[a]+ Jared S. Wood,[b]+ Paige Banks,[a]+ Kenneth Knott, [a] Jonathan S. Briganti,[c,d] Anthony J. Briganti,[c,d] Samuel V. G. McNally,[e] Paul E. Marek,[f] Anne M. Brown,[c,d] Tappey H. Jones,[g] R. Thomas Williamson,[b] Emily Mevers[a]**

[a]Department of Chemistry, Virginia Tech, Blacksburg, Virginia, USA

[b]Department of Chemistry & Biochemistry, University of North Carolina Wilmington, Wilmington, NC

[c]Department of Biochemistry, Virginia Tech, Blacksburg, VA

[d]University Libraries, Virginia Tech, Blacksburg, VA

[e]Condor Country Consulting, Inc, Martinez, CA

[f]Department of Entomology, Virginia Tech, Blacksburg, VA

[g]Department of Chemistry, Virginia Military Institute, Lexington, VA

+Authors contributed equally

Table of Contents

1. SUPPLEMENTARY FIGURES......4
FIGURE S1. GCMS TOTAL ION CHROMATOGRAM OF THE CRUDE MEOH EXTRACT OF THE *I. PLICATA* COLLECTIONS. 4
FIGURE S2. EIMS SPECTRUM FOR ISCHNOCYBINE A (**1**) M/Z 291......4
FIGURE S3. EIMS SPECTRUM FOR ISCHNOCYBINONE (**2**) M/Z 305......4
FIGURE S4. EIMS SPECTRUM FOR ISCHNOCYBINE B (**3**) M/Z 349......5
FIGURE S5. EIMS SPECTRUM FOR ISCHNOCYBINE C (**4**) M/Z 379......5
FIGURE S6. EIMS SPECTRUM FOR HYDROGENATED ISCHNOCYBINE A (**1**)......5
FIGURE S7. EIMS SPECTRUM FOR HYDROGENATED ISCHNOCYBINONE (**2**)......5
FIGURE S8. EIMS SPECTRUM FOR HYDROGENATED ISCHNOCYBINE B (**3**)......5
FIGURE S9. CHROMATOGRAM OF BASE HYDROLYSIS OF *I. PLICATA* EXTRACT......6
FIGURE S10. EIMS SPECTRUM FOR METHANOLYSIS ADDUCT (PEAK 1) OF ISCHNOCYBINE A (**1**)......6
FIGURE S11. EIMS SPECTRUM FOR METHANOLYSIS ADDUCT (PEAK 5) OF ISCHNOCYBINONE (**2**)...6
FIGURE S12. EIMS SPECTRUM FOR METHANOLYSIS ADDUCT (PEAK 6) OF ISCHNOCYBINE B (**3**)......6
FIGURE S13. EIMS SPECTRUM FOR METHANOLYSIS ADDUCT (PEAK 3) OF ISCHNOCYBINE C (**4**)......6
FIGURE S14. EIMS SPECTRUM FOR METHOXYLAMINE ADDUCT OF ISCHNOCYBINONE (**2**)......7
FIGURE S15. HRESIMS $[M+H]^+$ SPECTRUM OF ISCHNOCYBINE A (**1**)......7
FIGURE S16. HRESIMS $[M+H]^+$ SPECTRUM OF ISCHNOCYBINONE (**2**)......8
FIGURE S17. HRESIMS $[M+H]^+$ SPECTRUM OF ISCHNOCYBINE B (**3**)......8
FIGURE S18. HRESIMS $[M+H]^+$ SPECTRUM OF ISCHNOCYBINE C (**4**)......9
FIGURE S19. ^{1}H NMR SPECTRUM OF ISCHNOCYBINE A (**1**) [500 MHZ, DMSO-D_6]......9
FIGURE S20. ^{13}C NMR SPECTRUM OF ISCHNOCYBINE A (**1**) [500 MHZ, DMSO-D_6]......10
FIGURE S21. MULTIPLICITY-EDITED ^{1}H-^{13}C HSQC NMR SPECTRUM OF ISCHNOCYBINE A (**1**)......10
FIGURE S22. COSY NMR SPECTRUM OF ISCHNOCYBINE A (**1**) [500 MHZ, DMSO-D_6]. 11
FIGURE S23. ^{1}H-^{13}C HMBC NMR SPECTRUM OF ISCHNOCYBINE A (**1**) [500 MHZ, DMSO-D_6]. 11
FIGURE S24. EASY ROESY NMR SPECTRUM OF ISCHNOCYBINE A (**1**) [500 MHZ, DMSO-D_6]......12
FIGURE S25. ^{1}H NMR SPECTRUM OF ISCHNOCYBINONE (**2**) [500 MHZ, DMSO-D_6]......12
FIGURE S26. ^{13}C NMR SPECTRUM OF ISCHNOCYBINONE (**2**) [500 MHZ, DMSO-D_6]......13
FIGURE S27. MULTIPLICITY-EDITED ^{1}H-^{13}C HSQC NMR SPECTRUM OF ISCHNOCYBINONE (**2**)......13
FIGURE S28. COSY NMR SPECTRUM OF ISCHNOCYBINONE (**2**) [500 MHZ, DMSO-D_6]. 14
FIGURE S29. ^{1}H-^{13}C HMBC NMR SPECTRUM OF ISCHNOCYBINONE (**2**) [500 MHZ, DMSO-D_6]......14
FIGURE S30. EASY ROESY NMR SPECTRUM OF ISCHNOCYBINONE (**2**) [500 MHZ, DMSO-D_6]......15
FIGURE S31. ^{1}H NMR SPECTRUM OF ISCHNOCYBINE B (**3**) [500 MHZ, DMSO-D_6]......15
FIGURE S32. ^{13}C NMR SPECTRUM OF ISCHNOCYBINE B (**3**) [500 MHZ, DMSO-D_6]......16
FIGURE S33. MULTIPLICITY-EDITED ^{1}H-^{13}C HSQC NMR SPECTRUM OF ISCHNOCYBINE B (**3**)......16
FIGURE S34. COSY NMR SPECTRUM OF ISCHNOCYBINE B (**3**) [500 MHZ, DMSO-D_6]......17
FIGURE S35. ^{1}H-^{13}C HMBC NMR SPECTRUM OF ISCHNOCYBINE B (**3**) [500 MHZ, DMSO-D_6]......17
FIGURE S36. EASY ROESY NMR SPECTRUM OF ISCHNOCYBINE B (**3**) [500 MHZ, DMSO-D_6]......18
FIGURE S37. ^{1}H NMR SPECTRUM OF ISCHNOCYBINE C (**4**) [600 MHZ, DMSO-D_6]......18
FIGURE S38. ^{13}C NMR SPECTRUM OF ISCHNOCYBINE C (**4**) [500 MHZ, DMSO-D_6]......19
FIGURE S39. MULTIPLICITY-EDITED ^{1}H-^{13}C HSQC NMR SPECTRUM OF ISCHNOCYBINE C (**4**)......19
FIGURE S40. COSY NMR SPECTRUM OF ISCHNOCYBINE C (**4**) [500 MHZ, DMSO-D_6]......20
FIGURE S41. ^{1}H-^{13}C HMBC NMR SPECTRUM OF ISCHNOCYBINE C (**4**) [500 MHZ, DMSO-D_6]......20
FIGURE S42. EASY ROESY NMR SPECTRUM OF ISCHNOCYBINE C (**4**) [500 MHZ, DMSO-D_6]......21
FIGURE S43. ^{1}H NMR SPECTRUM OF ISCHNOCYBINE A ALCOHOL (**6**) [600 MHZ, DMSO-D_6]......21
FIGURE S44. MULTIPLICITY-EDITED ^{1}H-^{13}C HSQC NMR SPECTRUM OF ISCHNOCYBINE A ALC (**6**)..22
FIGURE S45. DQFCOSY NMR SPECTRUM OF ISCHNOCYBINE A ALC. (**6**) [600 MHZ, DMSO-D_6]. 22
FIGURE S46. ^{1}H-^{13}C HMBC NMR SPECTRUM OF ISCHNOCYBINE A ALC. (**6**) [600 MHZ, DMSO-D_6]......23
FIGURE S47. EASY ROESY NMR SPECTRUM OF ISCHNOCYBINE A ALC. (**6**) [600 MHZ, DMSO-D_6]......23
FIGURE S48. H2BC NMR SPECTRUM OF ISCHNOCYBINE A ALC.(**6**) [600 MHZ, DMSO-D_6]......24
FIGURE S49. MOSHER'S ANALYSIS OF ISCHNOCYBINE A (**1**)......24
FIGURE S50. ALL PRELIMINARY PDSP DATA GRAPHED......25
FIGURE S51. SIGMA-1 AND SIGMA-2 BINDING CURVES FOR COMPOUND **6**......26
FIGURE S52. NE-100 DOCKED INTO THE AGONIST $Σ_1$ RECEPTOR......26
FIGURE S53. PENTAZOCINE DOCKED INTO THE ANTAGONIST $Σ_1$ RECEPTOR......27

FIGURE S54. REDOCKING OF PENTAZOCINE AND NE-100 INTO AGONIST AND ANTAGONIST Σ_1 RECEPTOR STRUCTURES. 27
FIGURE S55. ISCHNOCYBINE A-SSR (**1**) DOCKED INTO THE AGONIST Σ_1 RECEPTOR....28
FIGURE S56. ISCHNOCYBINE B-RRSR (**3**) DOCKED INTO THE AGONIST Σ_1 RECEPTOR....28
FIGURE S57. ISCHNOCYBINE C-SRSSS (**4**) DOCKED INTO THE AGONIST Σ_1 RECEPTOR....29
FIGURE S58. ISCHNOCYBINONE-RSR (**2**) DOCKED INTO THE AGONIST Σ_1 RECEPTOR....29
2. SUPPLEMENTARY TABLE....30
TABLE S1. NMR SPECTROSCOPY DATA (500 MHZ, D_6-DMSO) FOR ISCHNOCYBINE A (**1**)....30
TABLE S2. NMR SPECTROSCOBY DATA (500 MHZ, D_6-DMSO) FOR ISCHNOCYBINONE (**2**) 31
TABLE S3. NMR SPECTROSCOPY DATA (500 MHZ, D_6-DMSO) FOR ISCHNOCYBINE B (**3**)....32
TABLE S4. NMR SPECTROSCOPY DATA (500 MHZ, D_6-DMSO) FOR ISCHNOCYBINE C (**4**)....33
TABLE S5. ^{13}C NMR CHEMICAL SHIFTS PREDICTIONS FOR ISCHNOCYBINE A (**1**) 34
TABLE S6. ^{1}H NMR CHEMICAL SHIFT PREDICTIONS FOR ISCHNOCYBINE A (**1**)....35
TABLE S7. ^{13}C NMR CHEMICAL SHIFTS PREDICTIONS FOR ISCHNOCYBINONE (**2**) 36
TABLE S8. ^{1}H NMR CHEMICAL SHIFT PREDICTIONS FOR ISCHNOCYBINONE (**2**)....37
TABLE S9. ^{13}C NMR CHEMICAL SHIFTS PREDICTIONS FOR ISCHNOCYBINE B (**3**) 38
TABLE S10. ^{1}H NMR CHEMICAL SHIFT PREDICTIONS FOR ISCHNOCYBINE B (**3**)....39
TABLE S11. ^{13}C NMR CHEMICAL SHIFTS PREDICTIONS FOR ISCHNOCYBINE C (**4**)....40
TABLE S12. ^{13}C NMR CHEMICAL SHIFTS PREDICTIONS FOR ISCHNOCYBINE C (**4**) (CONT.) 41
TABLE S13. ^{1}H NMR CHEMICAL SHIFT PREDICTIONS FOR ISCHNOCYBINE C (**4**)....42
TABLE S14. ^{1}H NMR CHEMICAL SHIFT PREDICTIONS FOR ISCHNOCYBINE C (**4**) (CONT.) 43
TABLE S15. NMR SPECTROSCOPY DATA (600 MHZ, D_6-DMSO) FOR ISCHNOCYBINE A ALC. (**6**)....44
TABLE S16. ISOLATED QUANTITY OF ISCHNOCYBINES FROM 300 INDIVIDUAL *I. PLICATA*....44
TABLE S17. *I. PLICATA* LENGTH VERSUS ALKALOID STUDY 45
TABLE S18. PRIMARY BINDING ASSAY (INHIBITION %)....46
TABLE S19. INHIBITOR CONSTANT (K_I) FOR COMPOUNDS **1**–**4**....50
TABLE S20. MM/GBSA CALCULATED PREDICTED FREE ENERGY OF BINDING 50

1. Supplementary Figures

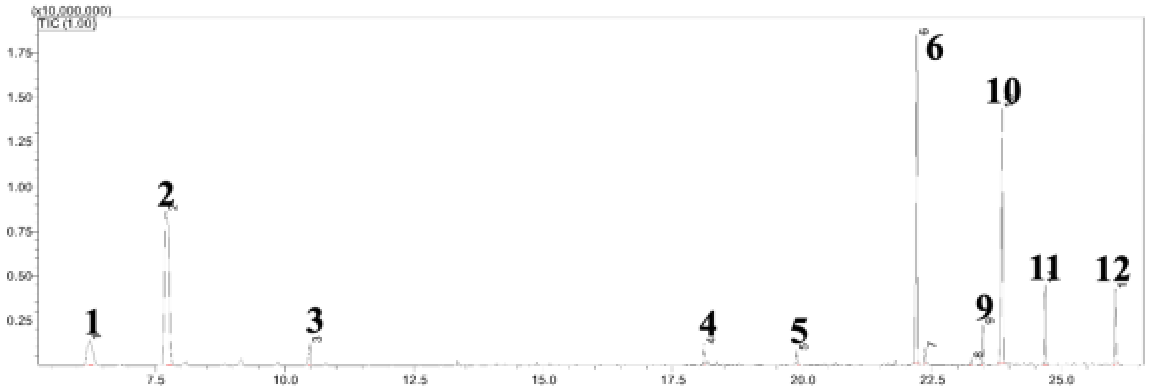

Peak	Compound ID	Rel amounts
**1**	α-pinene	6.1
**2**	β-pinene	32.6
**3**	α-terpenolene	1.9
**4**	germacreneD-4-ol	1.1
**5**	Ischnocybine A free alcohol (**6**)	0.9
**6**	Ischnocybine A (**1**)	24.5
**9**	FAME Me 9-octadecenoate	2.2
**10**	Ischnocybinone (**2**)	19.8
**11**	Ischnocybine B (**3**)	4.5
**12**	Ischnocybine C (**4**)	5.3

Figure S1. GCMS total ion chromatogram of the crude MeOH extract of the *I. plicata* collections.

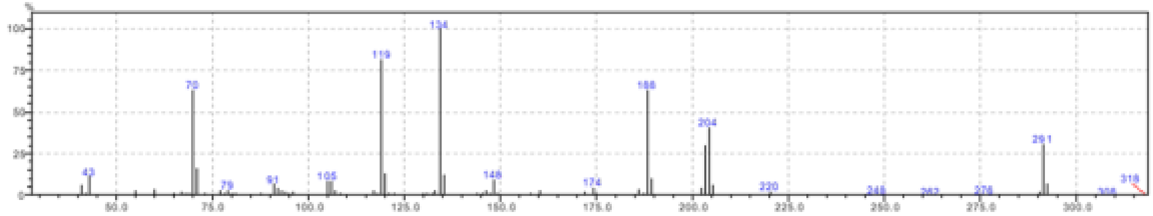

Figure S2. EIMS spectrum for ischnocybine A (**1**) m/z 291

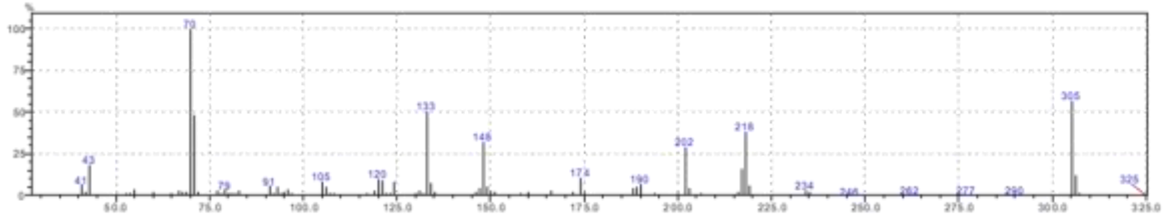

Figure S3. EIMS spectrum for ischnocybinone (**2**) m/z 305

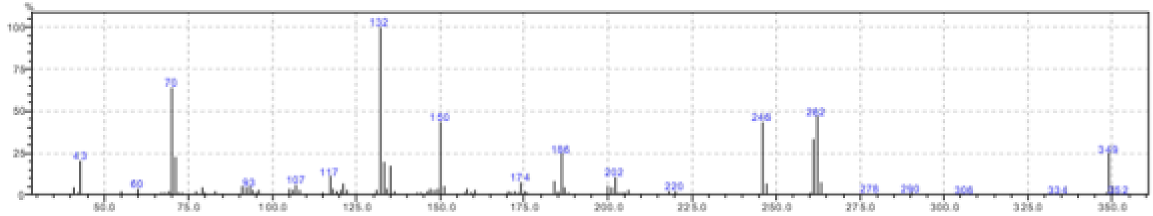

Figure S4. EIMS spectrum for ischnocybine B (**3**) m/z 349

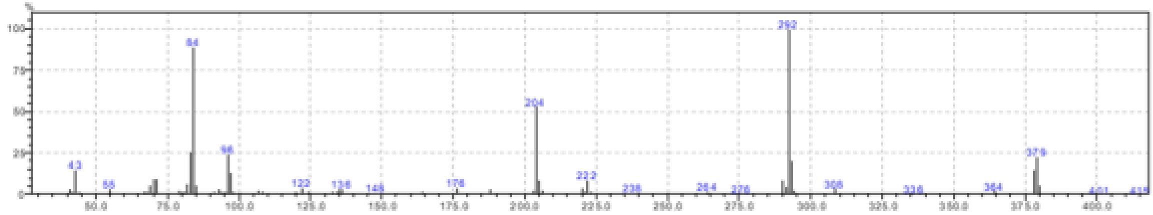

Figure S5. EIMS spectrum for ischnocybine C (**4**) m/z 379

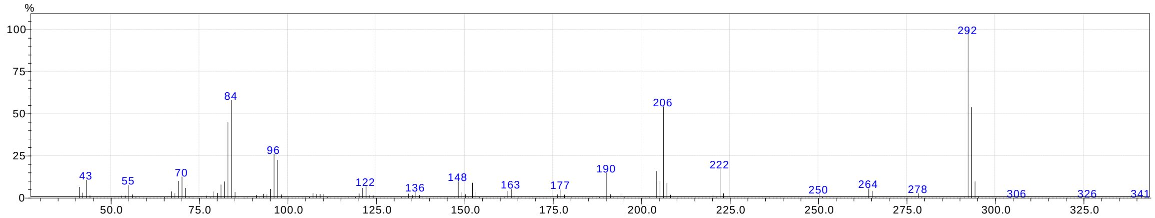

Figure S6. EIMS spectrum for hydrogenated ischnocybine A (**1**) (two isomers with m/z 293)

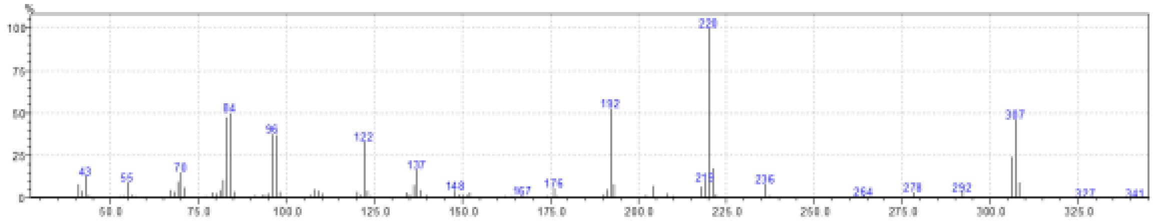

Figure S7. EIMS spectrum for hydrogenated ischnocybinone (**2**) (one isomer with m/z 307)

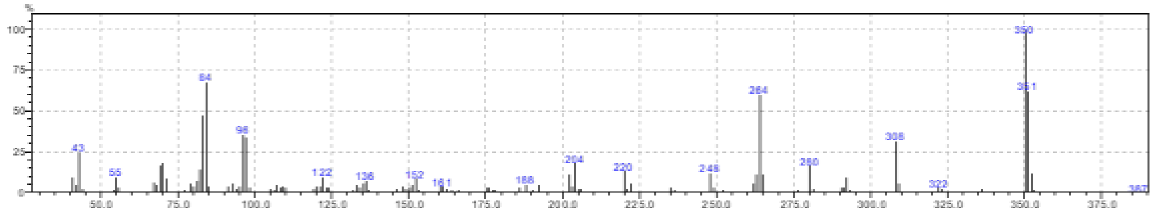

Figure S8. EIMS spectrum for hydrogenated ischnocybine B (**3**) (one isomer with m/z 351)

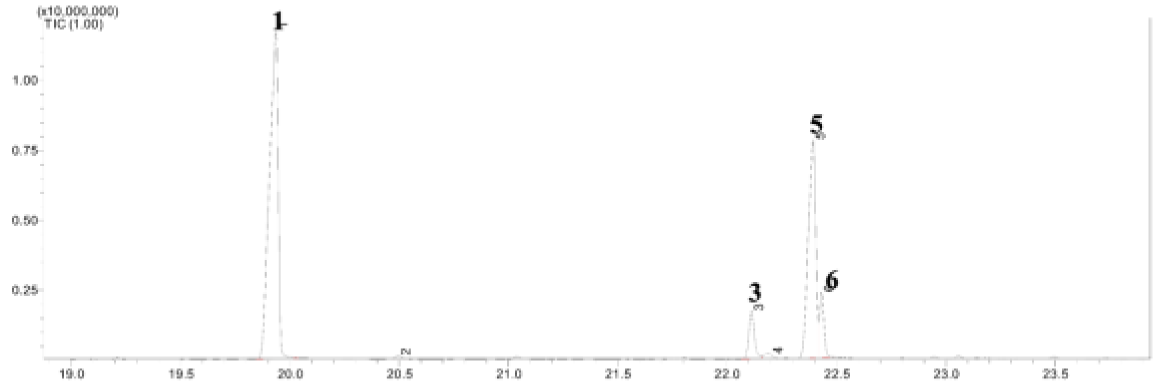

Figure S9. Chromatogram of base hydrolysis of *I. plicata* extract

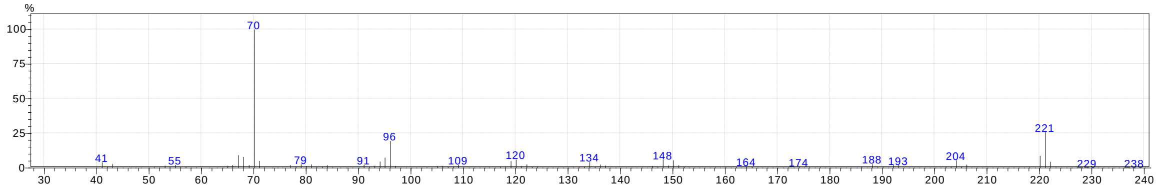

Figure S10. EIMS spectrum for methanolysis adduct (peak 1) of ischnocybine A (**1**) m/z 221.

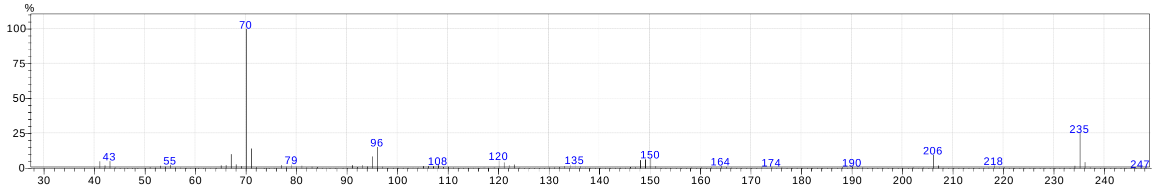

Figure S11. EIMS spectrum for methanolysis adduct (peak 5) of ischnocybinone (**2**) m/z 235.

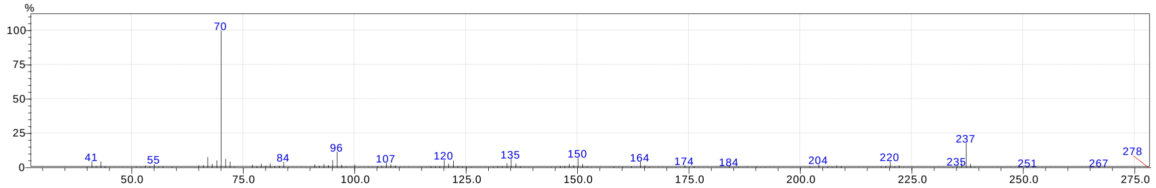

Figure S12. EIMS spectrum for methanolysis adduct (peak 6) of ischnocybine B (**3**) m/z 237.

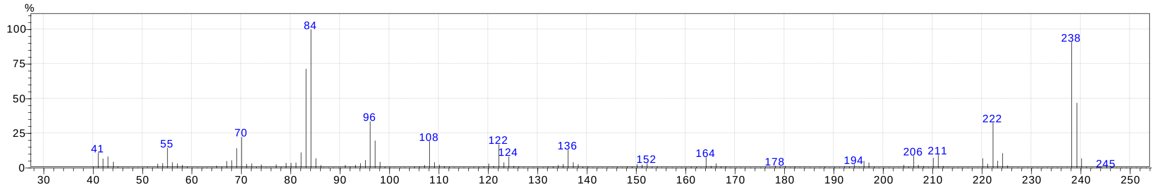

Figure S13. EIMS spectrum for methanolysis adduct (peak 3) of ischnocybine C (**4**) m/z 239.

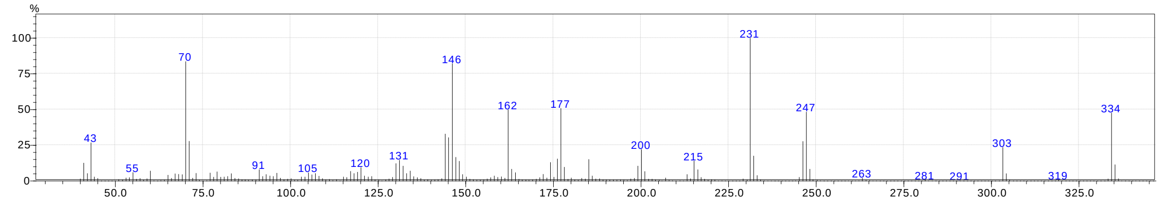

Figure S14. EIMS spectrum for methoxylamine adduct of ischnocybinone (**2**)

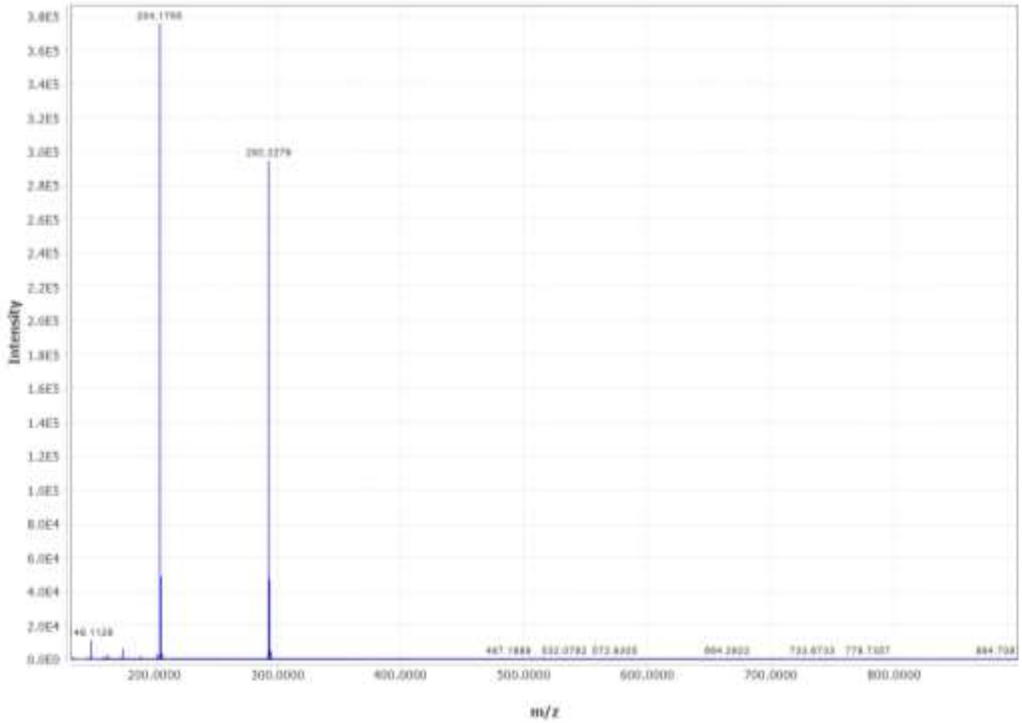

Figure S15. HRESIMS $[M+H]^+$ spectrum of ischnocybine A (**1**)

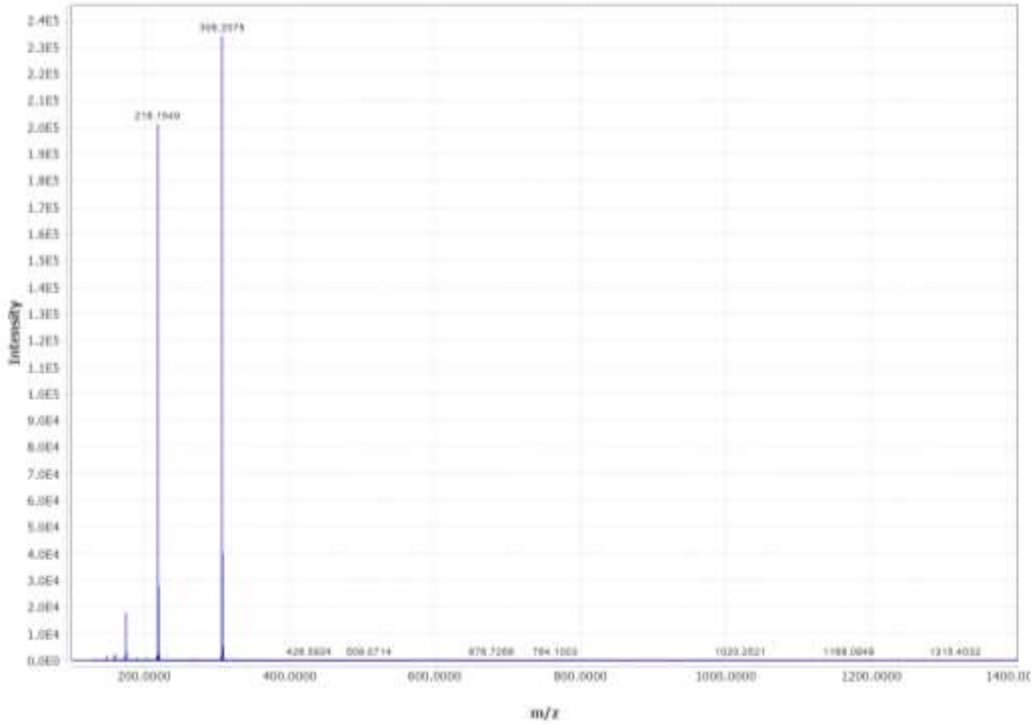

Figure S16. HRESIMS [M+H]$^+$ spectrum of ischnocybinone (**2**)

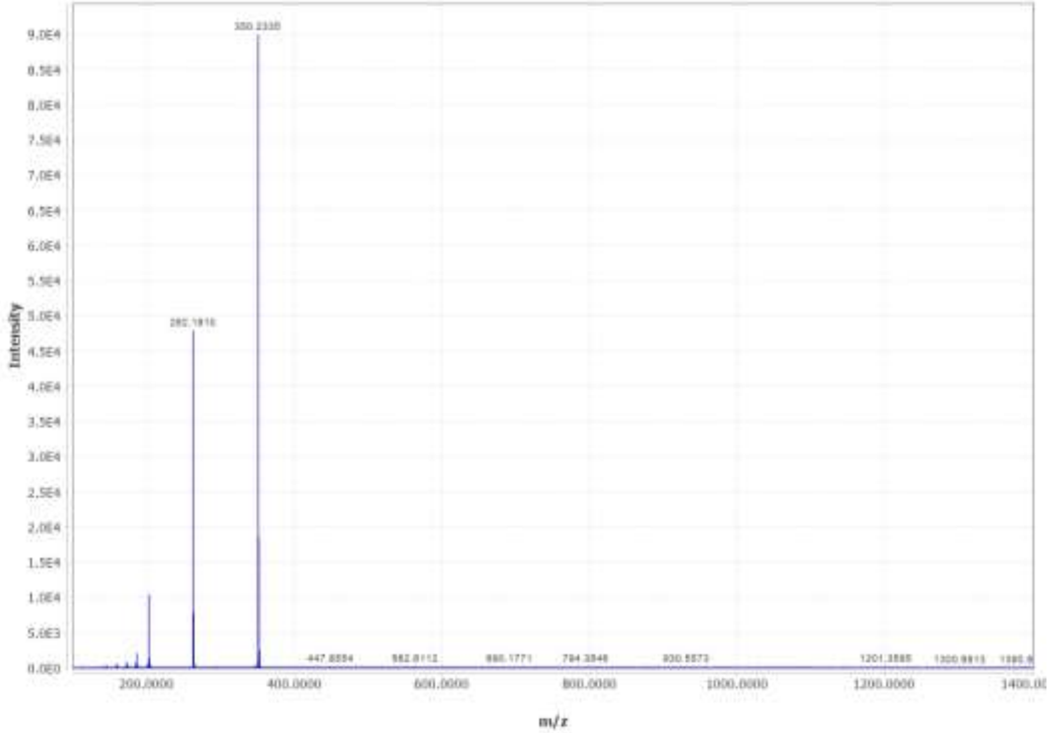

Figure S17. HRESIMS [M+H]$^+$ spectrum of ischnocybine B (**3**)

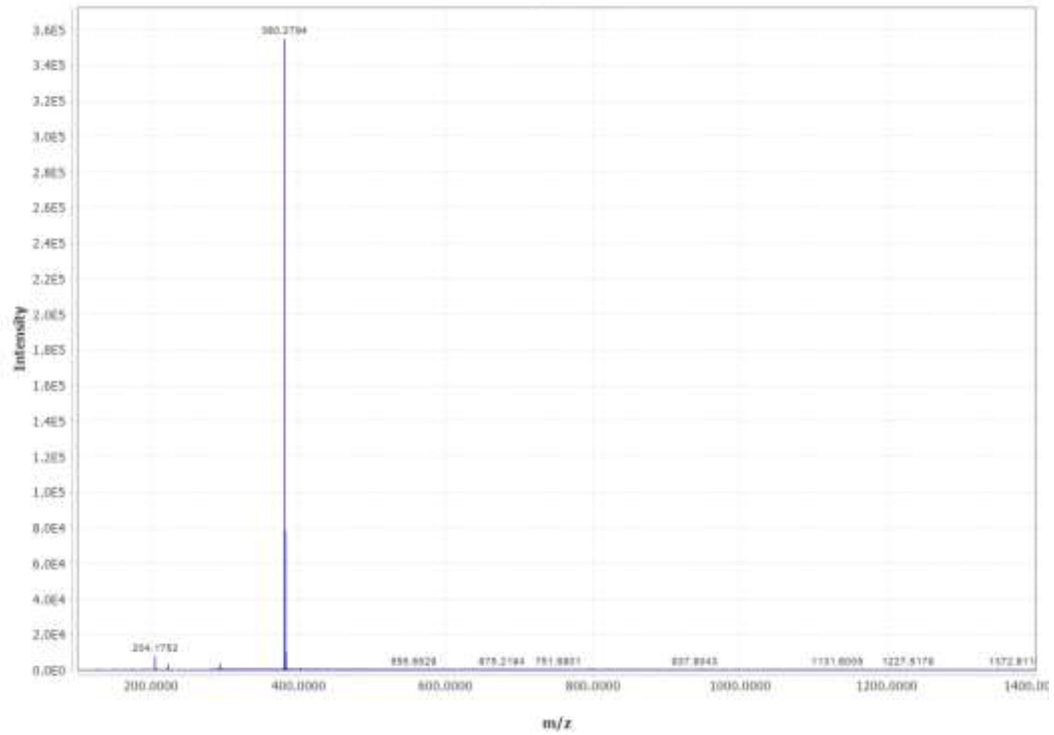

Figure S18. HRESIMS $[M+H]^+$ spectrum of ischnocybine C (**4**)

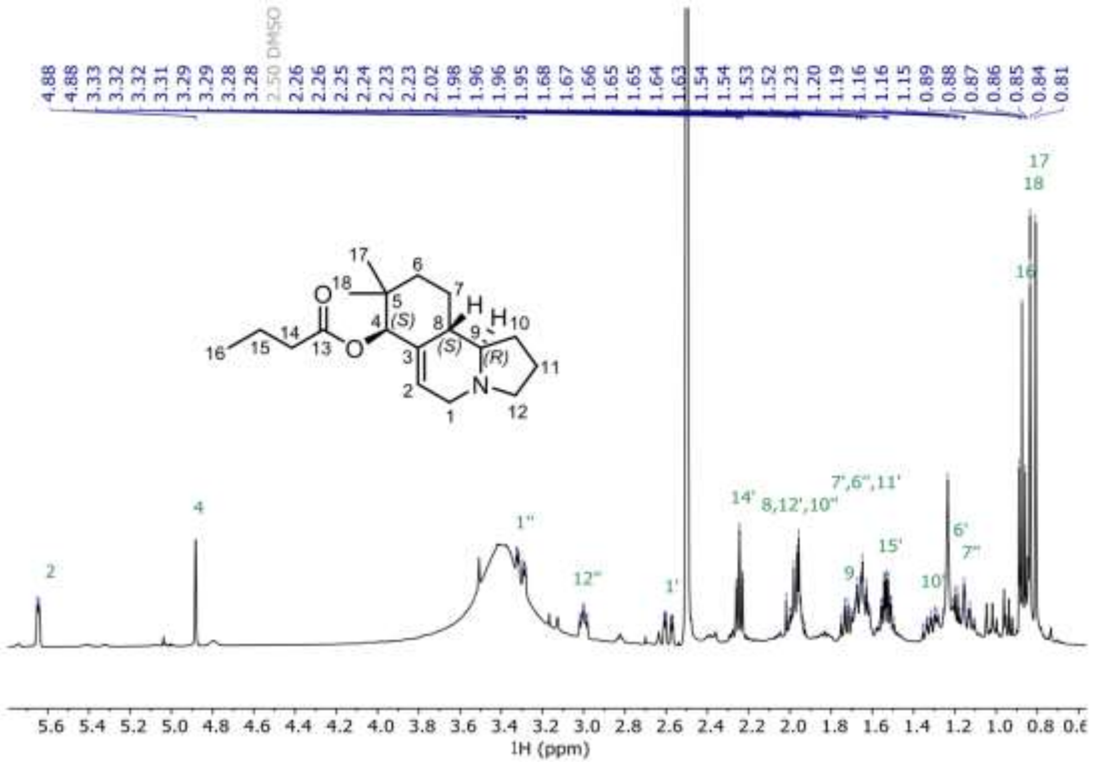

Figure S19. ^{1}H NMR spectrum of ischnocybine A (**1**) [500 MHz, DMSO-d_6].

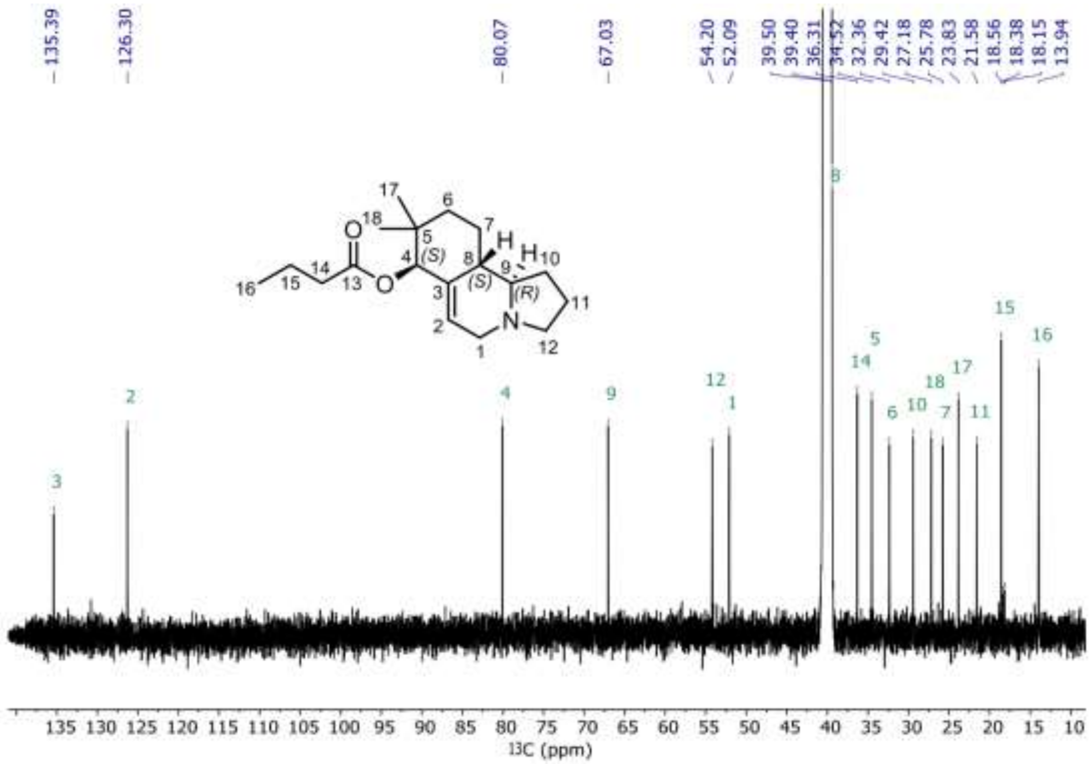

Figure S20. ^{13}C NMR spectrum of ischnocybine A (**1**) [500 MHz, DMSO-d_6].

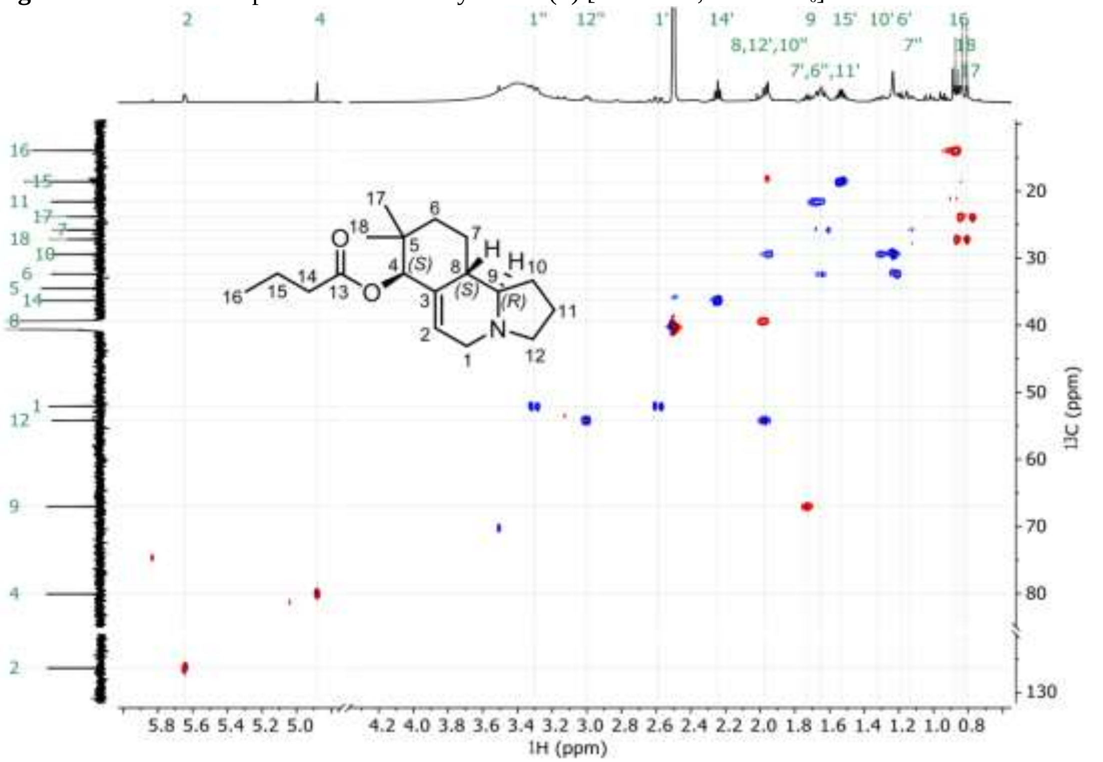

Figure S21. Multiplicity-edited ^1H-^{13}C HSQC NMR spectrum of ischnocybine A (**1**) [500 MHz, DMSO-d_6].

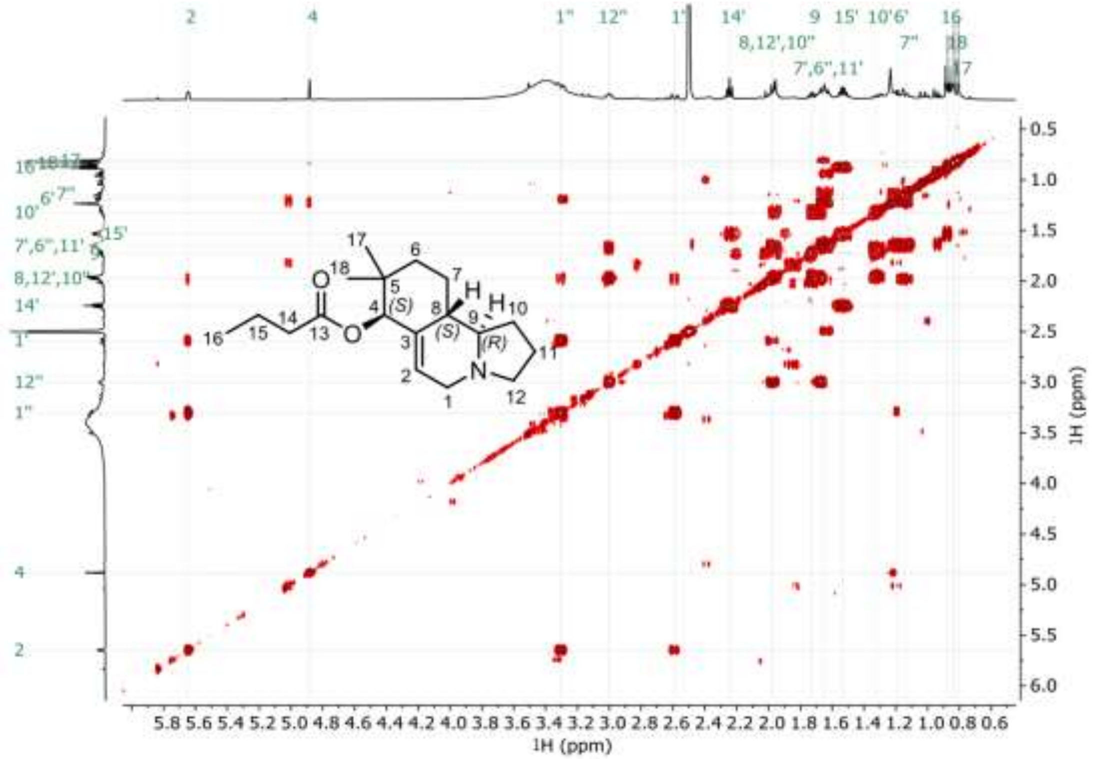

Figure S22. COSY NMR spectrum of ischnocybine A (**1**) [500 MHz, DMSO-d_6].

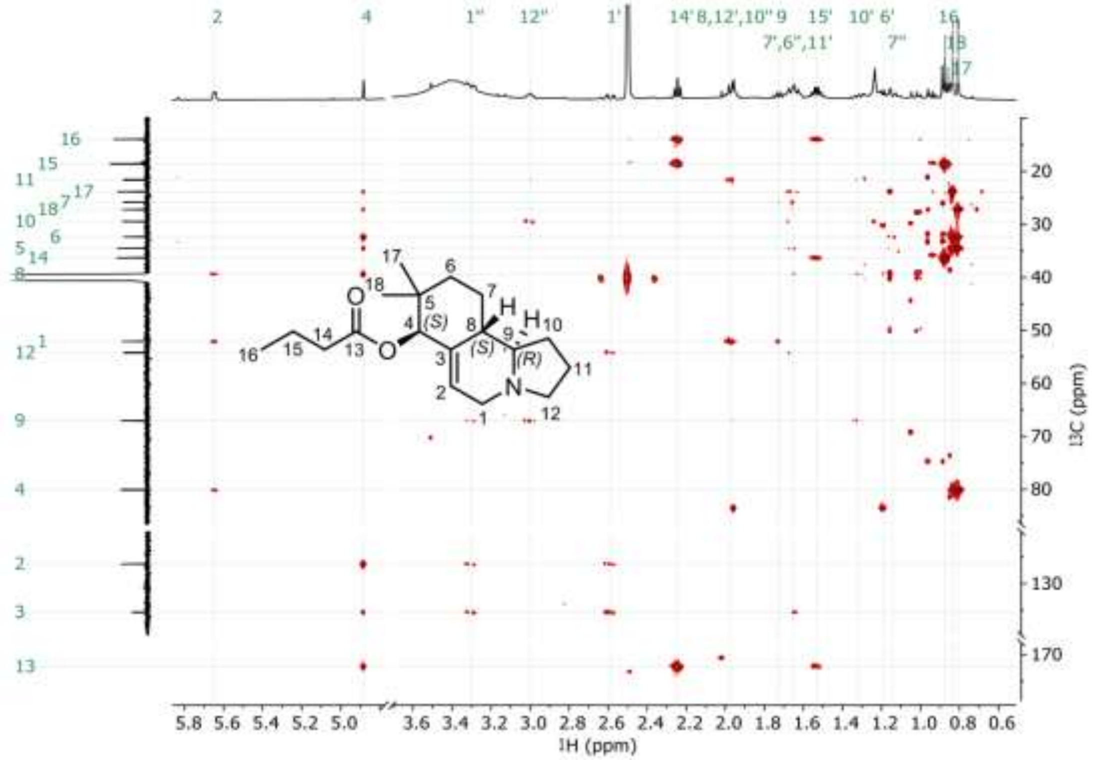

Figure S23. ^{1}H-^{13}C HMBC NMR spectrum of ischnocybine A (**1**) [500 MHz, DMSO-d_6].

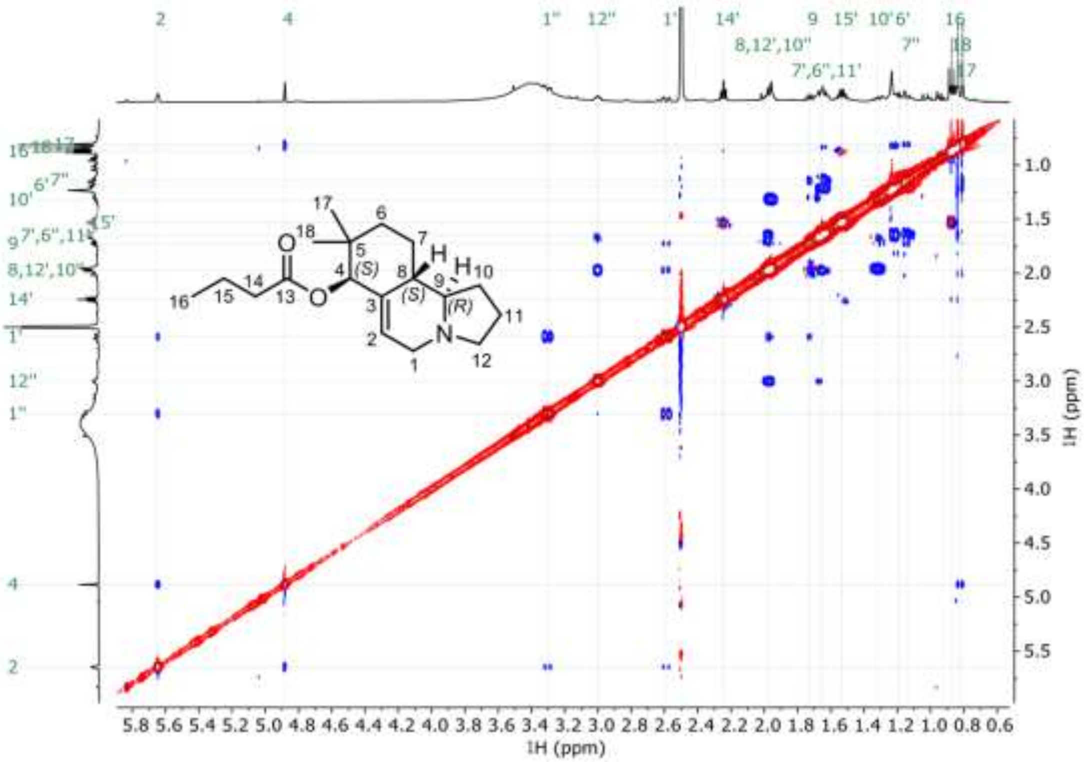

Figure S24. EASY ROESY NMR spectrum of ischnocybine A (**1**) [500 MHz, DMSO-d_6].

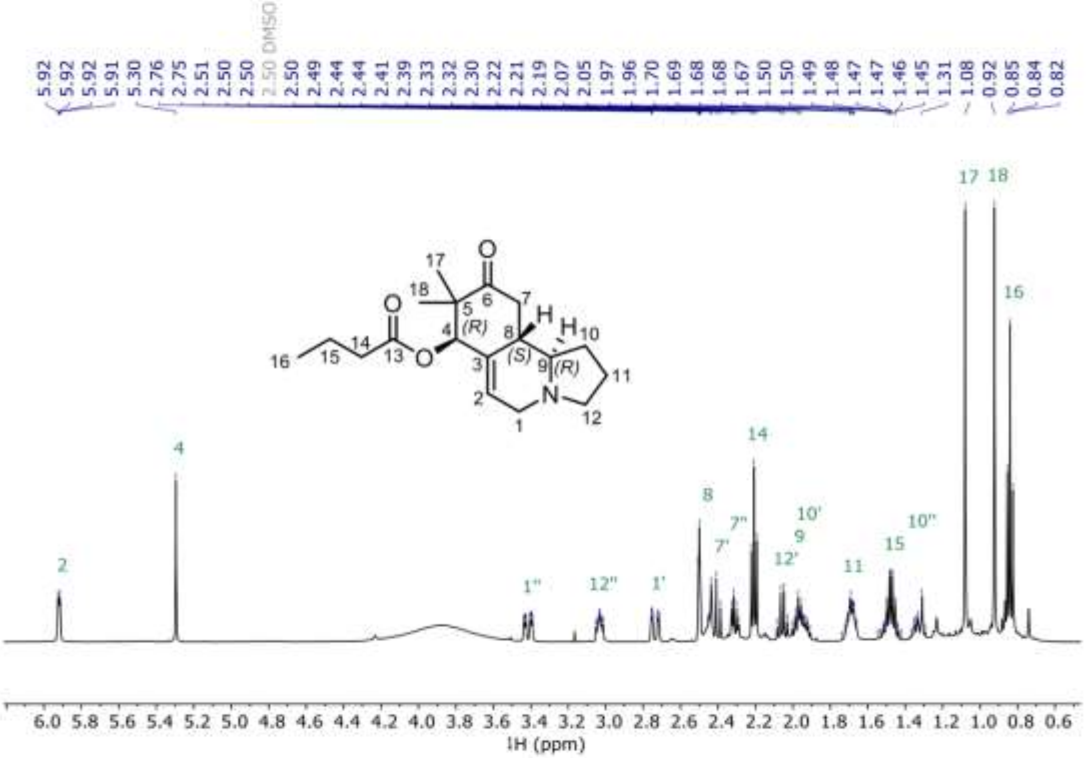

Figure S25. ^{1}H NMR spectrum of ischnocybinone (**2**) [500 MHz, DMSO-d_6].

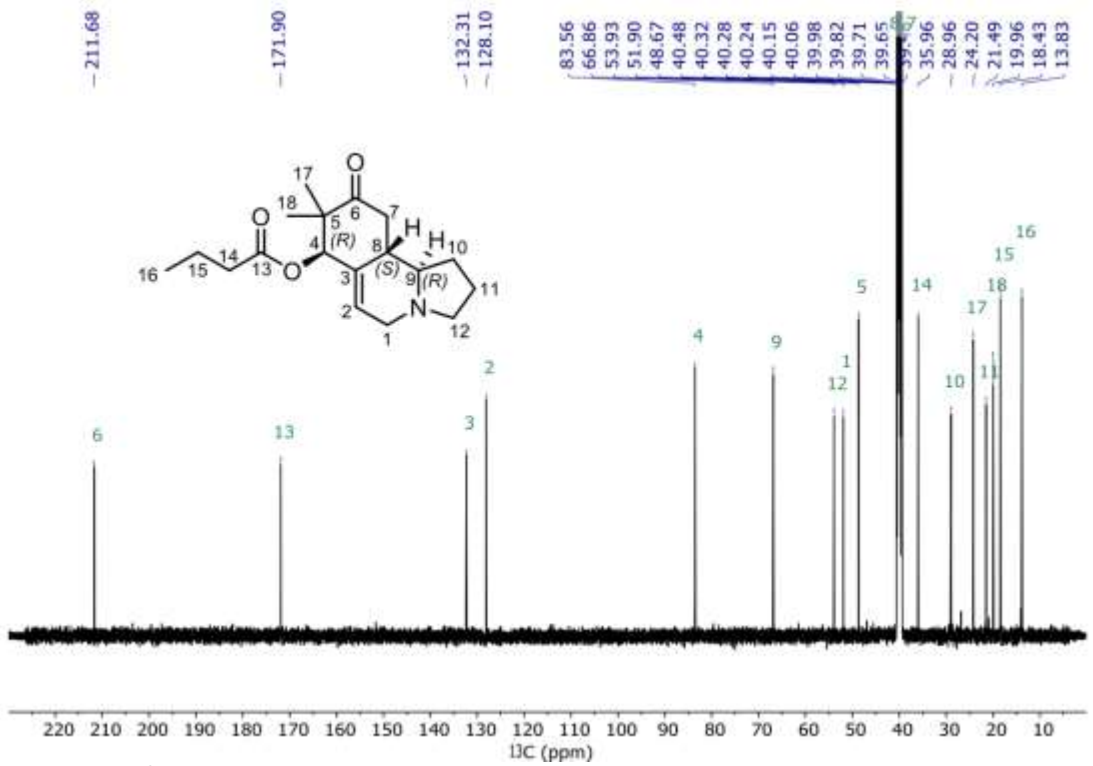

Figure S26. ^{13}C NMR spectrum of ischnocybinone (**2**) [500 MHz, DMSO-d_6].

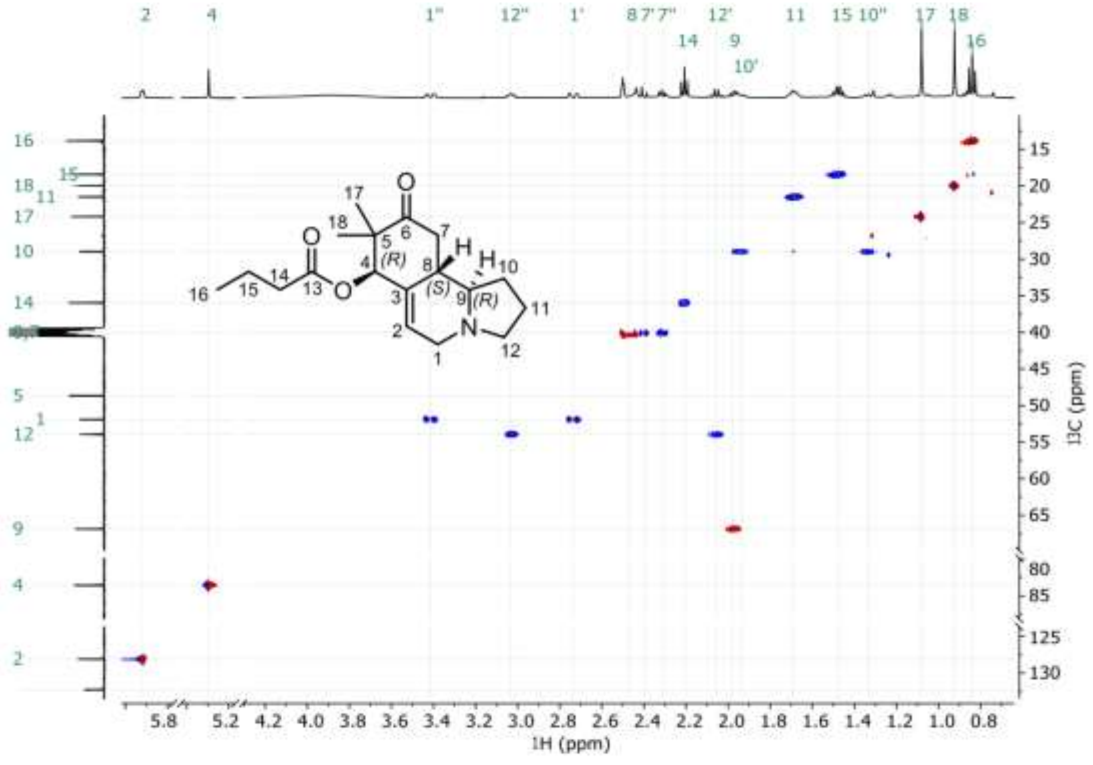

Figure S27. Multiplicity-edited ^{1}H-^{13}C HSQC NMR spectrum of ischnocybinone (**2**) [500 MHz, DMSO-d_6].

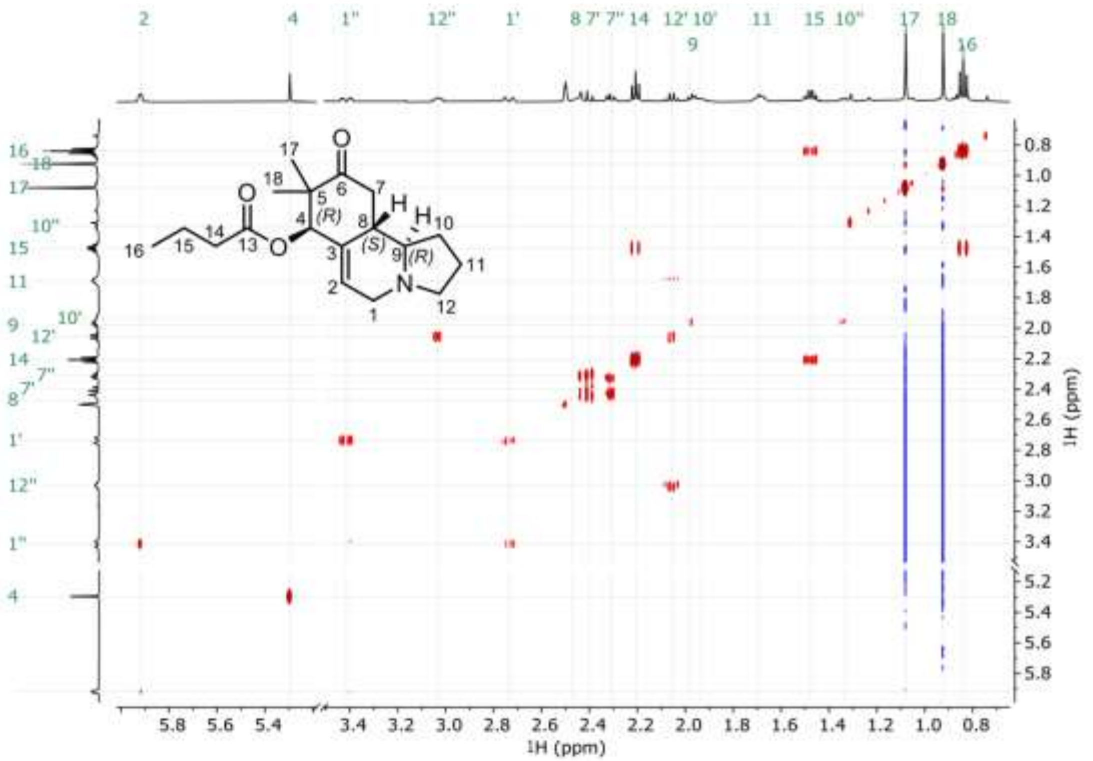

Figure S28. COSY NMR spectrum of ischnocybinone (**2**) [500 MHz, DMSO-d_6].

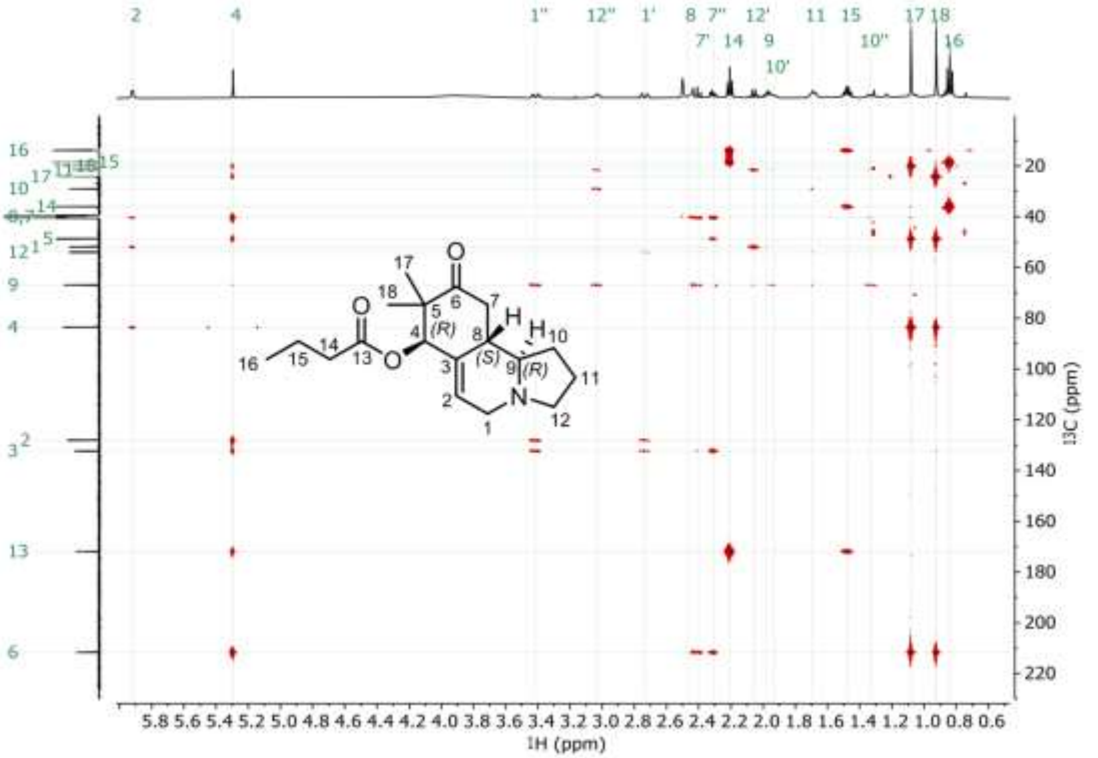

Figure S29. ^{1}H-^{13}C HMBC NMR spectrum of ischnocybinone (**2**) [500 MHz, DMSO-d_6].

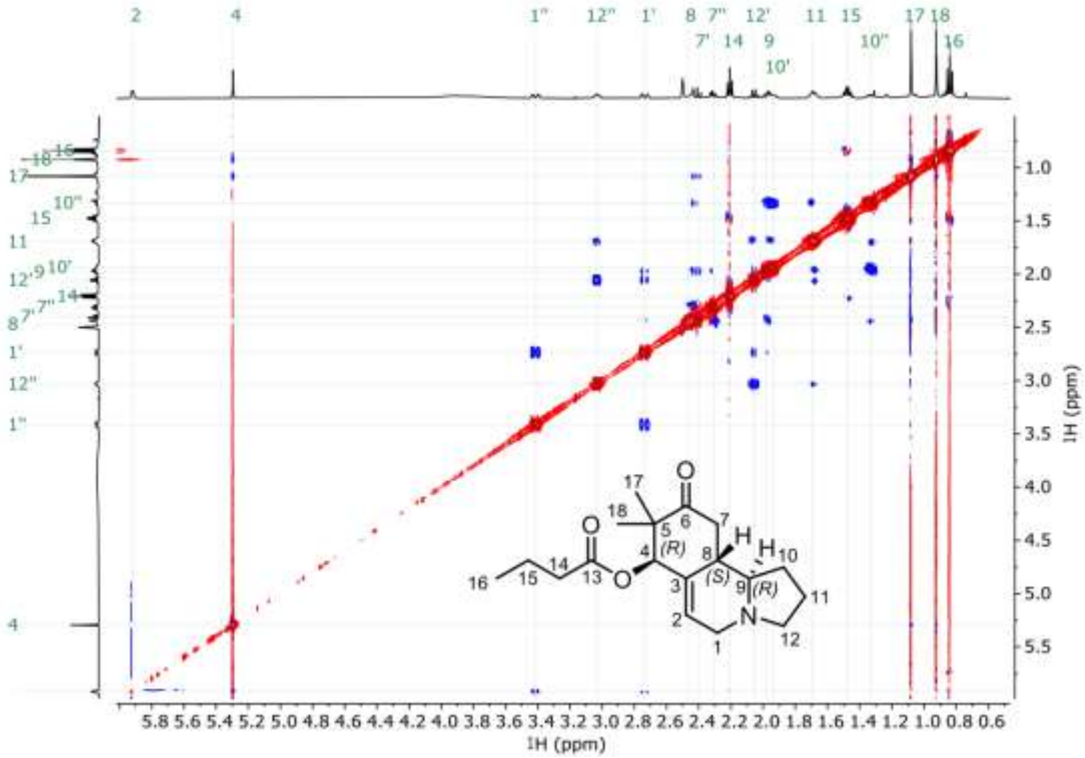

Figure S30. EASY ROESY NMR spectrum of ischnocybinone (**2**) [500 MHz, DMSO-d_6].

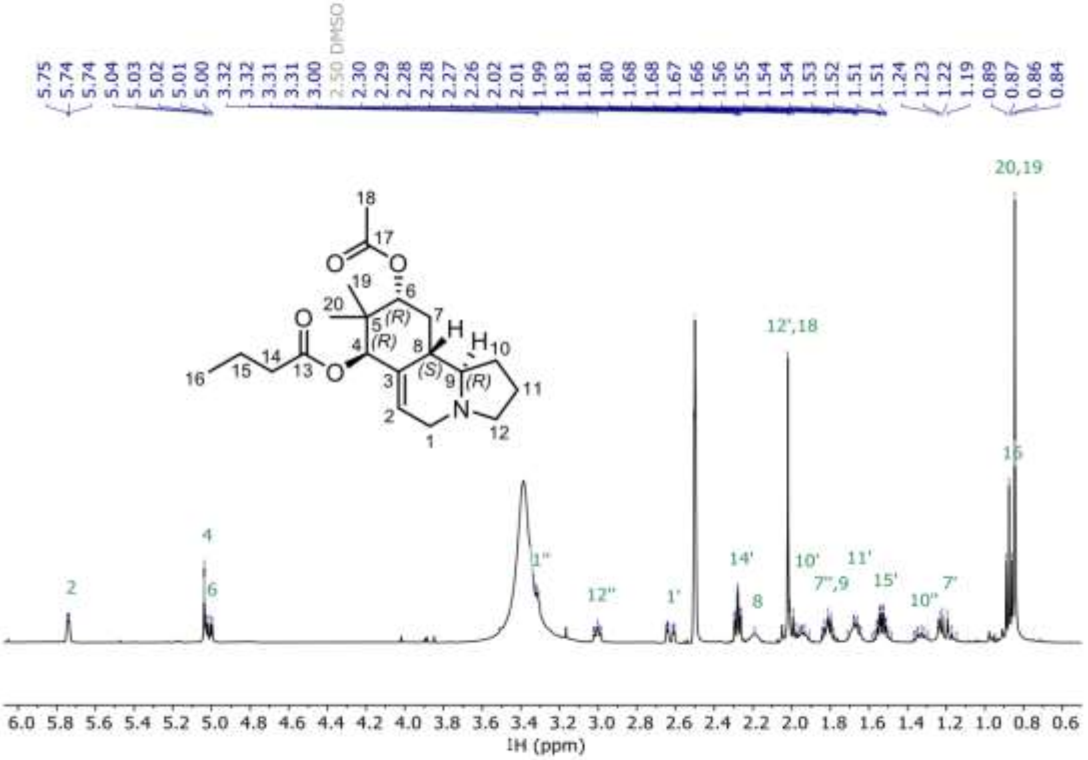

Figure S31. ^{1}H NMR spectrum of ischnocybine B (**3**) [500 MHz, DMSO-d_6].

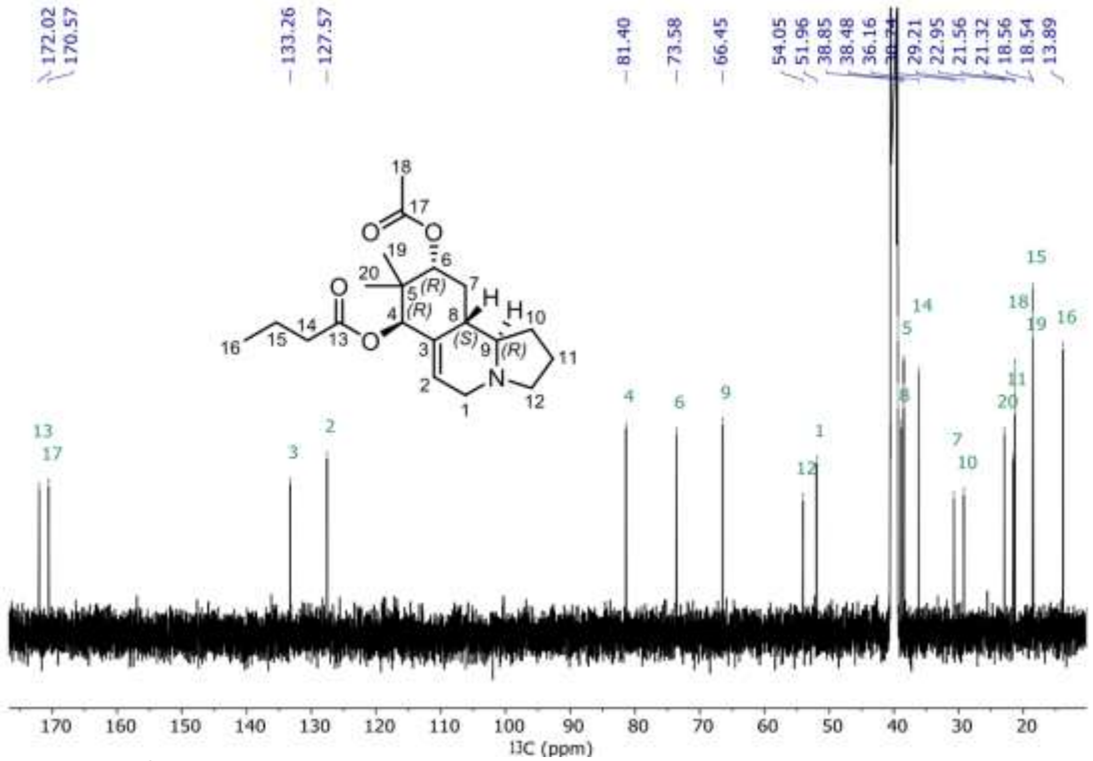

Figure S32. ^{13}C NMR spectrum of ischnocybine B (**3**) [500 MHz, DMSO-d_6].

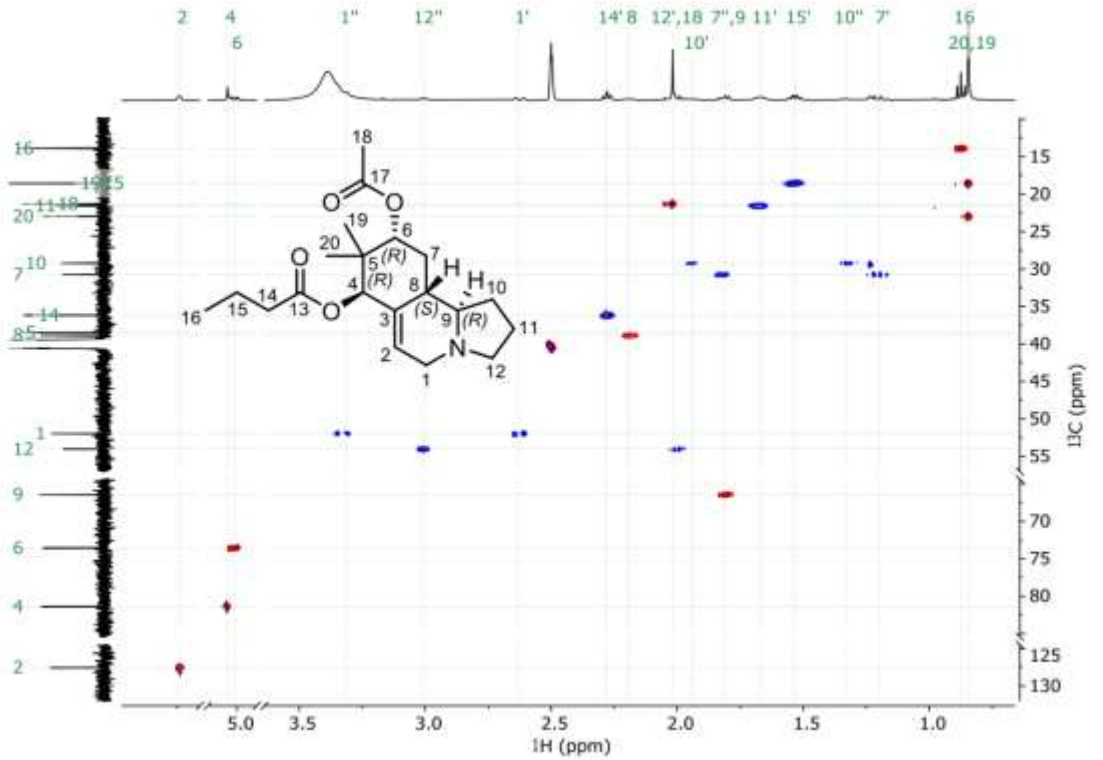

Figure S33. Multiplicity-edited ^{1}H-^{13}C HSQC NMR spectrum of ischnocybine B (**3**) [500 MHz, DMSO-d_6].

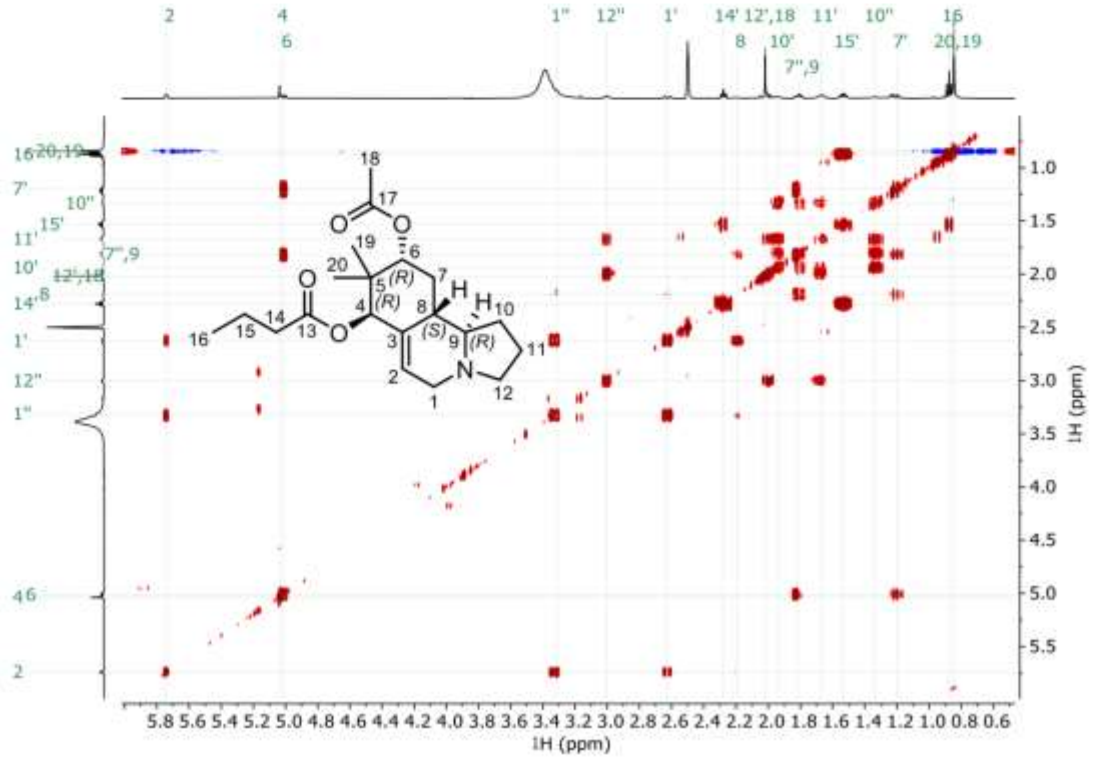

Figure S34. COSY NMR spectrum of ischnocybine B (**3**) [500 MHz, DMSO-d_6].

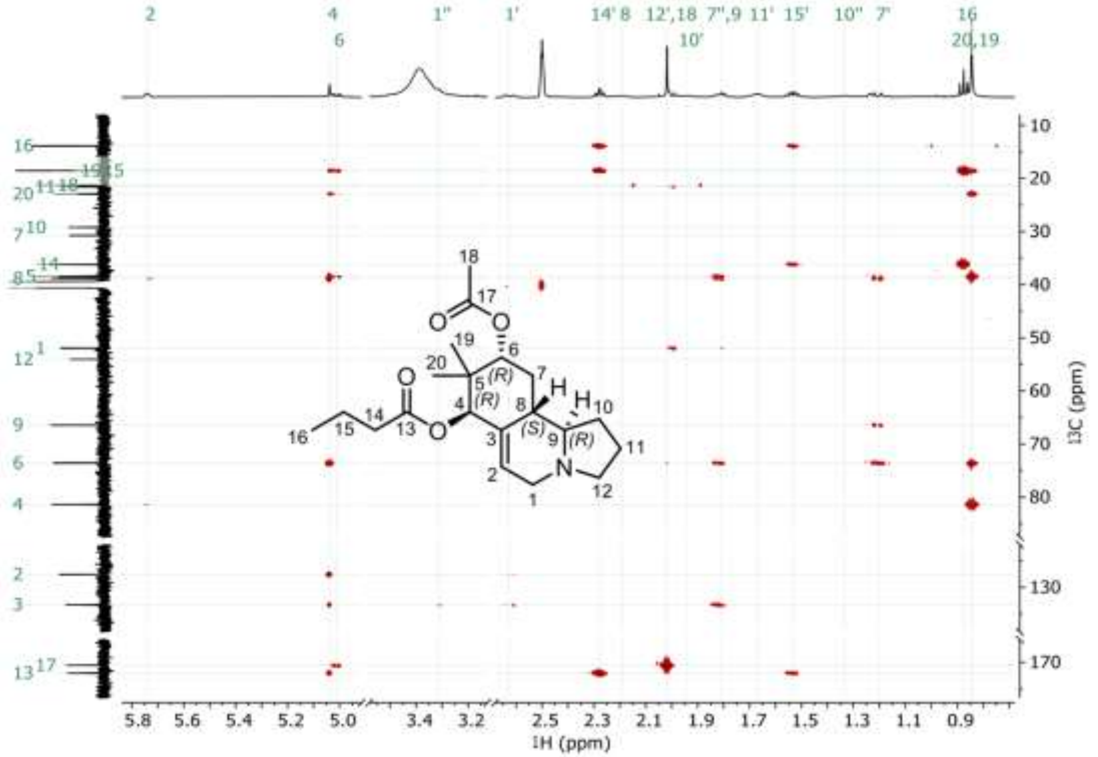

Figure S35. ^{1}H-^{13}C HMBC NMR spectrum of ischnocybine B (**3**) [500 MHz, DMSO-d_6].

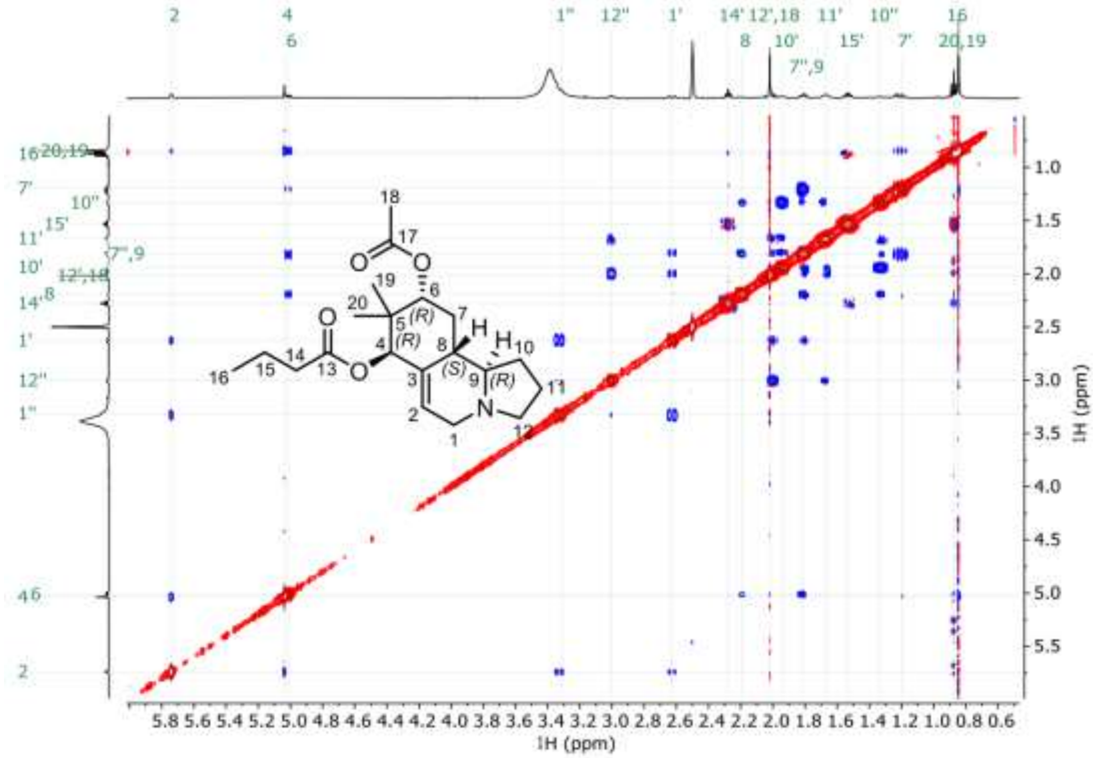

Figure S36. EASY ROESY NMR spectrum of ischnocybine B (**3**) [500 MHz, DMSO-d_6].

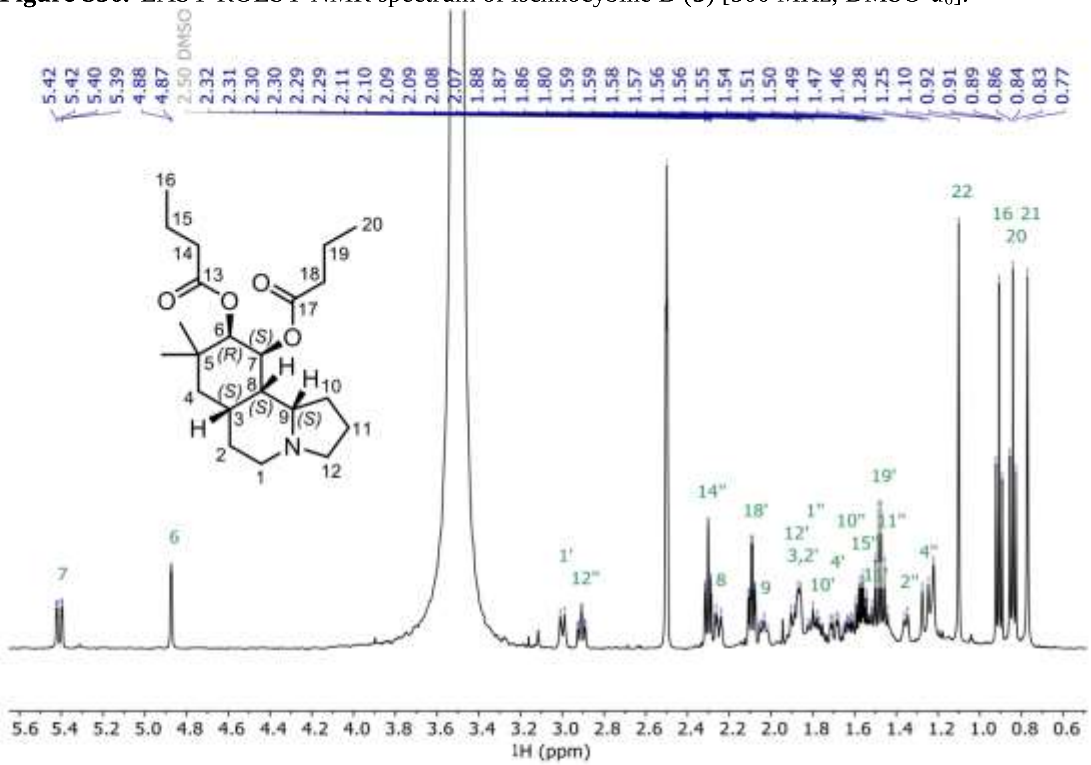

Figure S37. ^{1}H NMR spectrum of ischnocybine C (**4**) [600 MHz, DMSO-d_6].

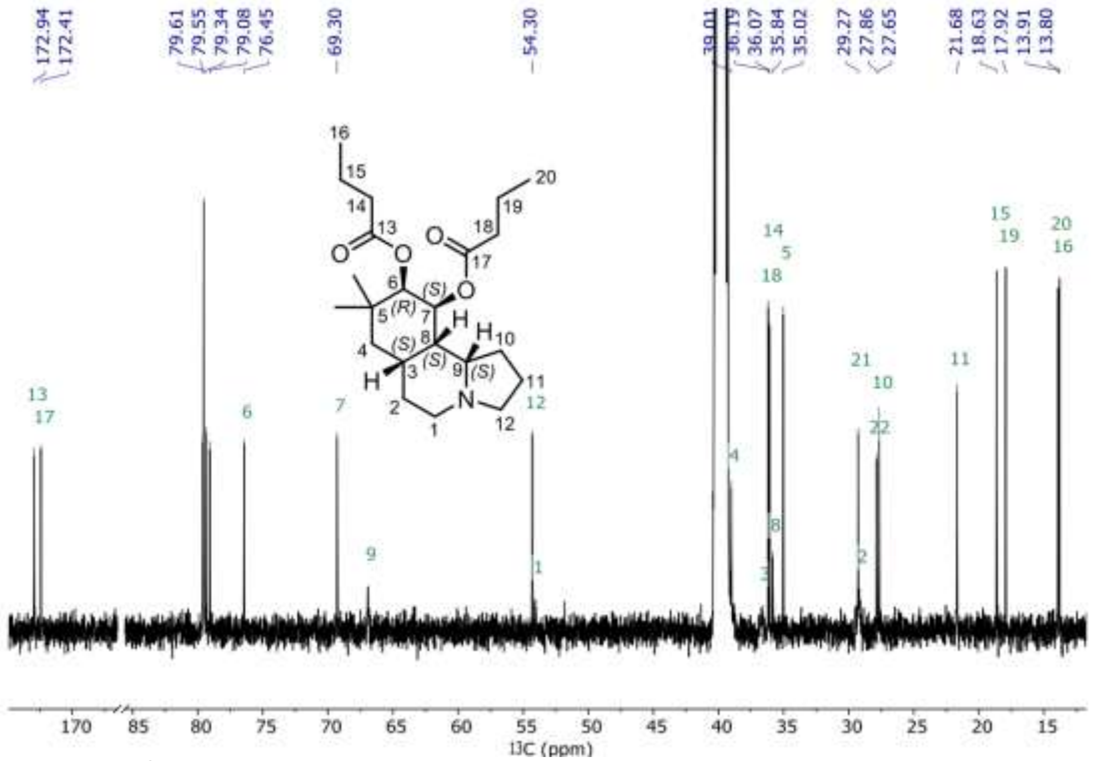

Figure S38. ^{13}C NMR spectrum of ischnocybine C (**4**) [500 MHz, DMSO-d_6].

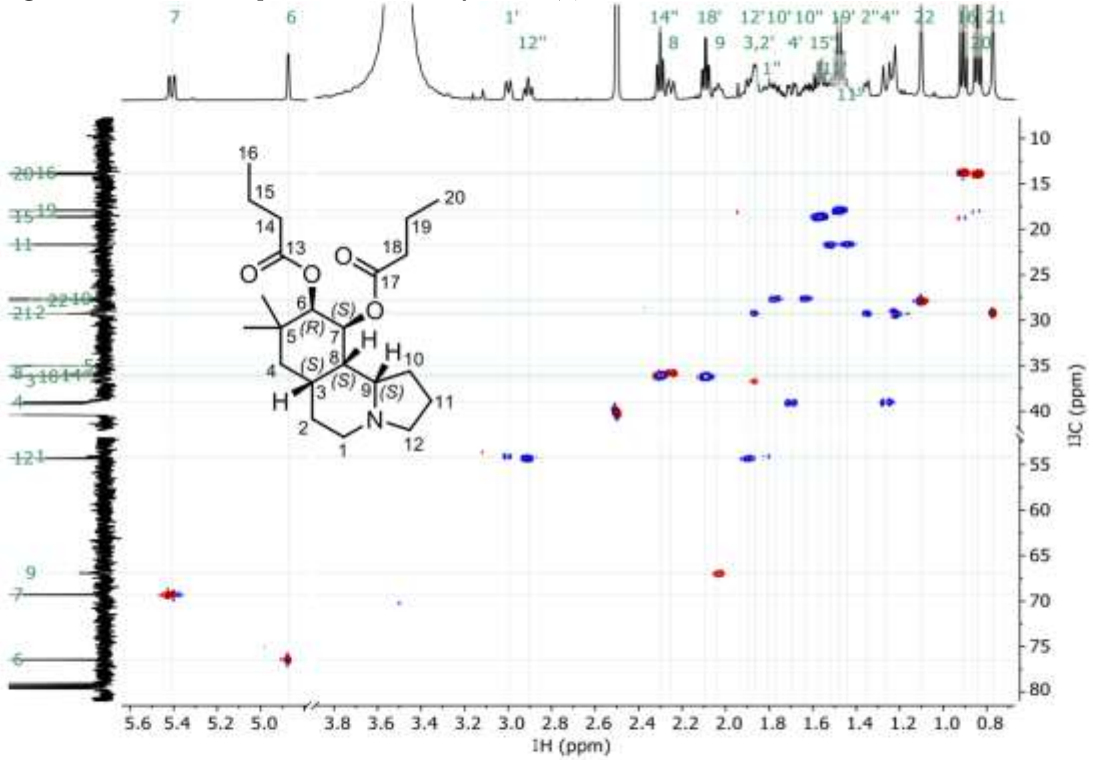

Figure S39. Multiplicity-edited ^{1}H-^{13}C HSQC NMR spectrum of ischnocybine C (**4**) [500 MHz, DMSO-d_6].

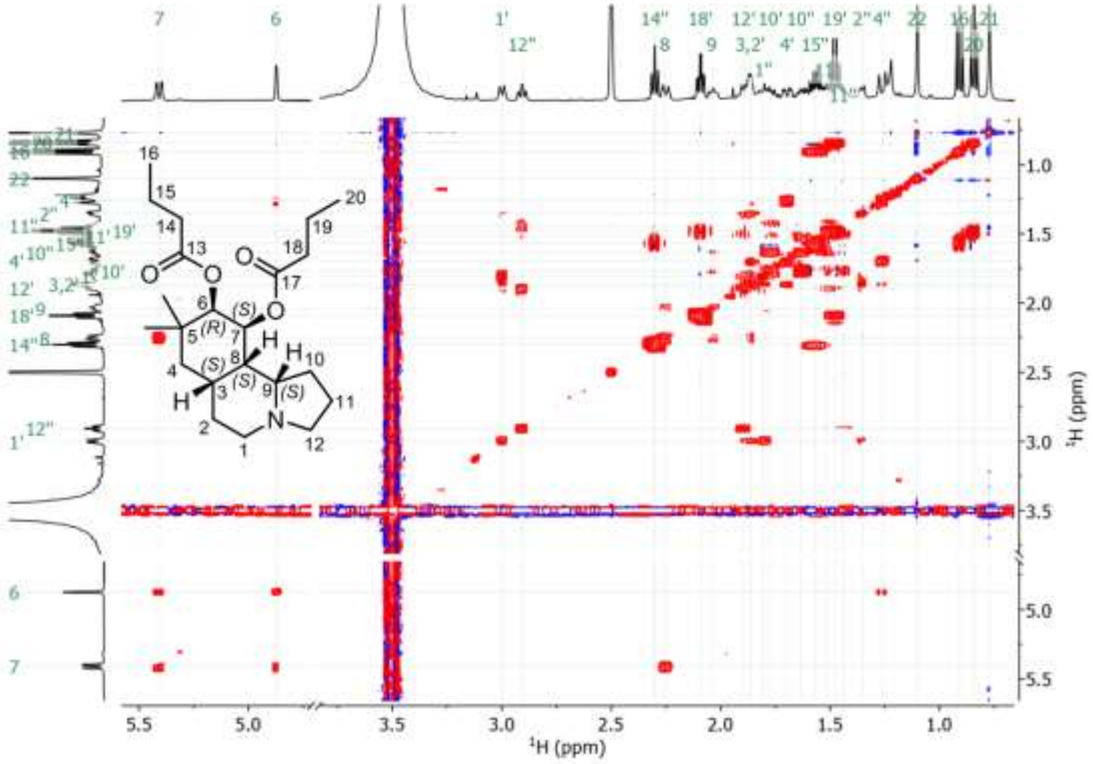

Figure S40. COSY NMR spectrum of ischnocybine C (**4**) [500 MHz, DMSO-d_6].

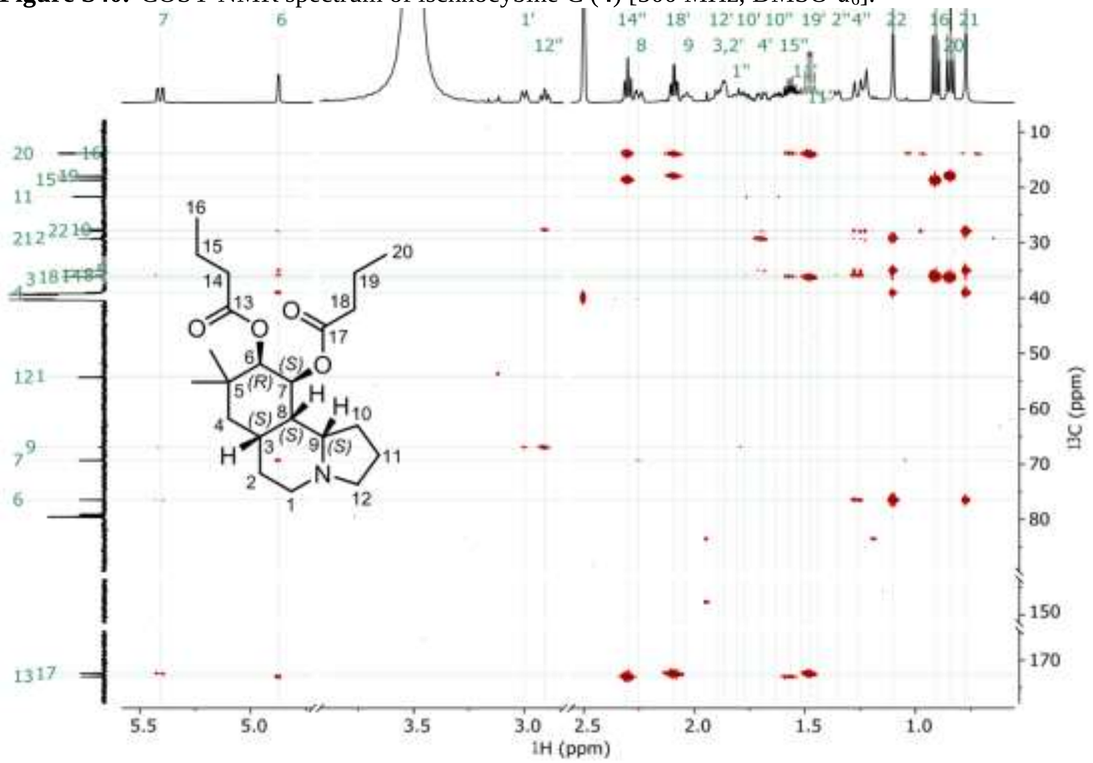

Figure S41. ^{1}H-^{13}C HMBC NMR spectrum of ischnocybine C (**4**) [500 MHz, DMSO-d_6].

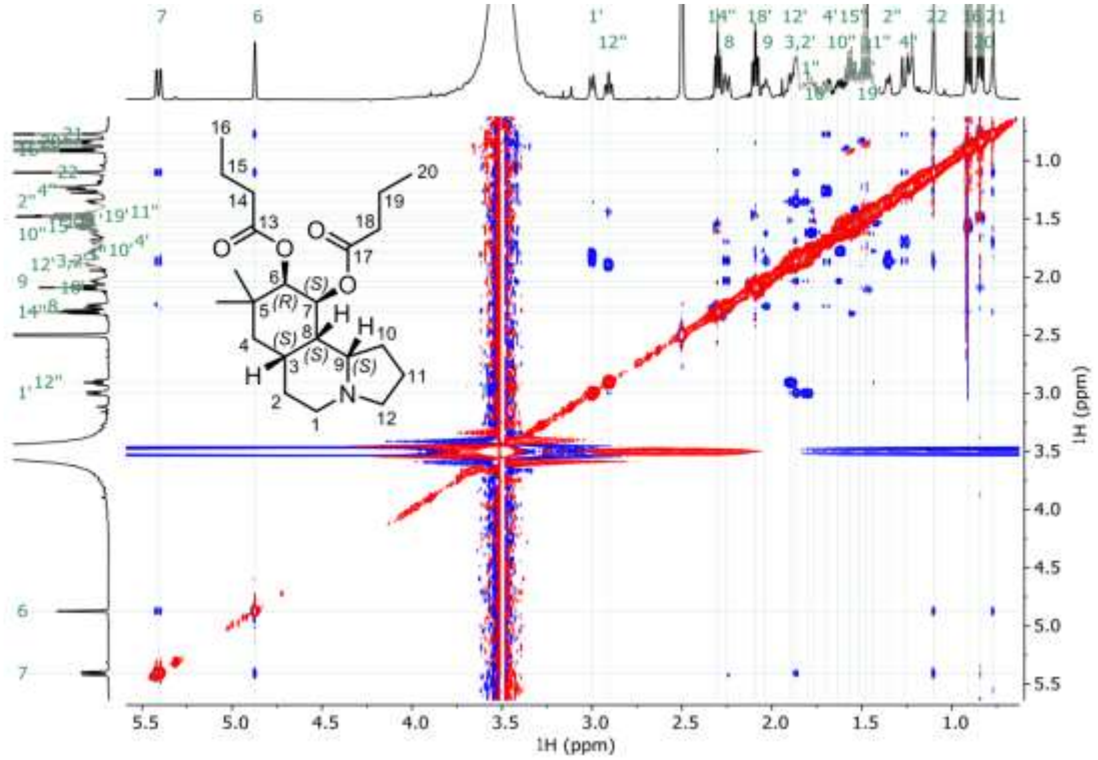

Figure S42. EASY ROESY NMR spectrum of ischnocybine C (**4**) [500 MHz, DMSO-d_6].

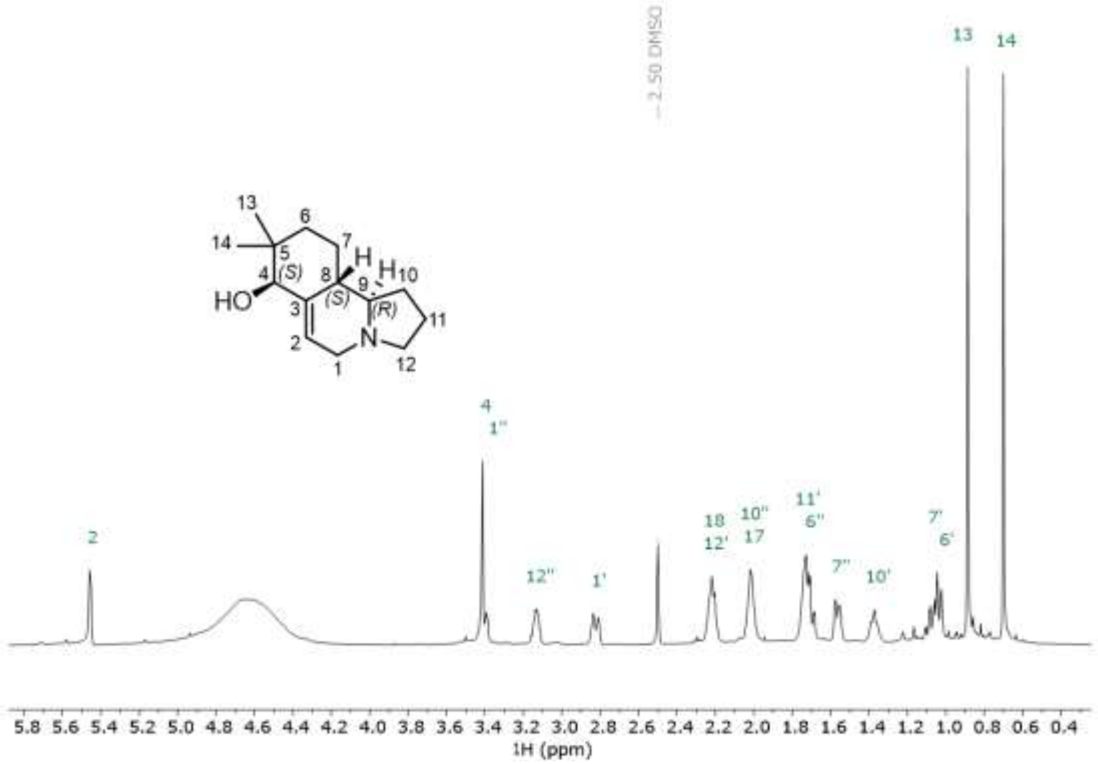

Figure S43. ^{1}H NMR spectrum of ischnocybine A alcohol (**6**) [600 MHz, DMSO-d_6].

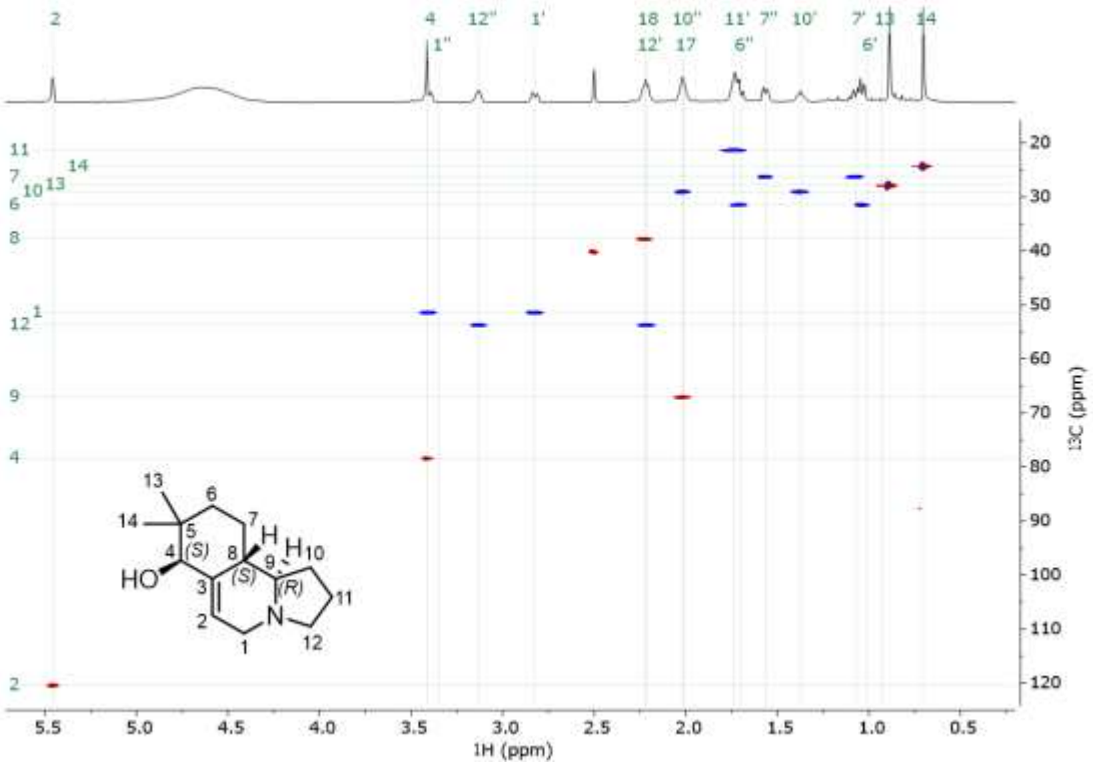

Figure S44. Multiplicity-edited ^{1}H-^{13}C HSQC NMR spectrum of ischnocybine A alcohol (**6**) [600 MHz, DMSO-d_6].

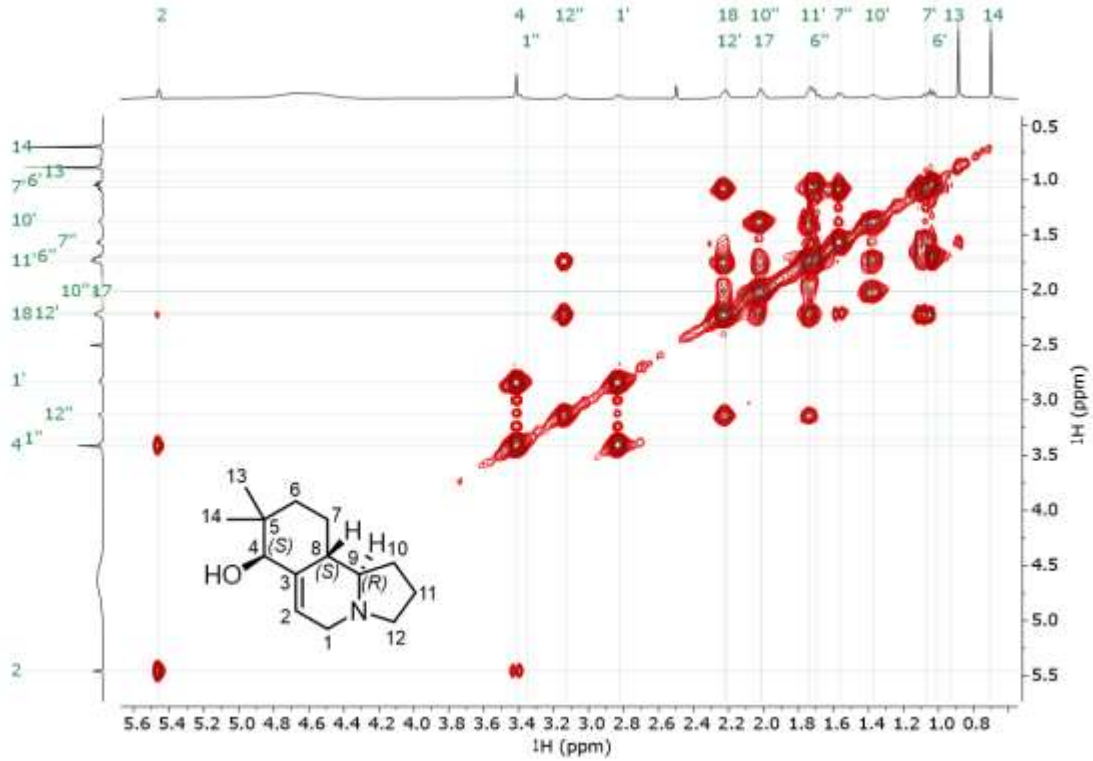

Figure S45. DQFCOSY NMR spectrum of ischnocybine A alcohol (**6**) [600 MHz, DMSO-d_6].

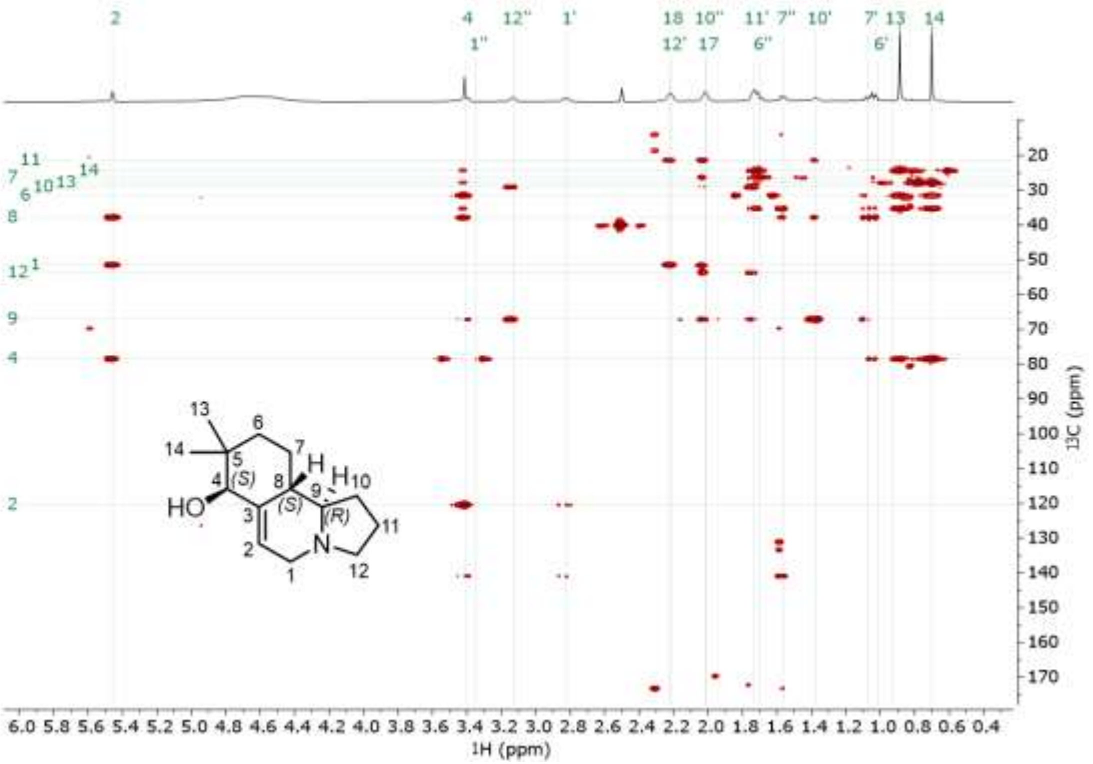

Figure S46. ^{1}H-^{13}C HMBC NMR spectrum of ischnocybine A alcohol (**6**) [600 MHz, DMSO-d_6].

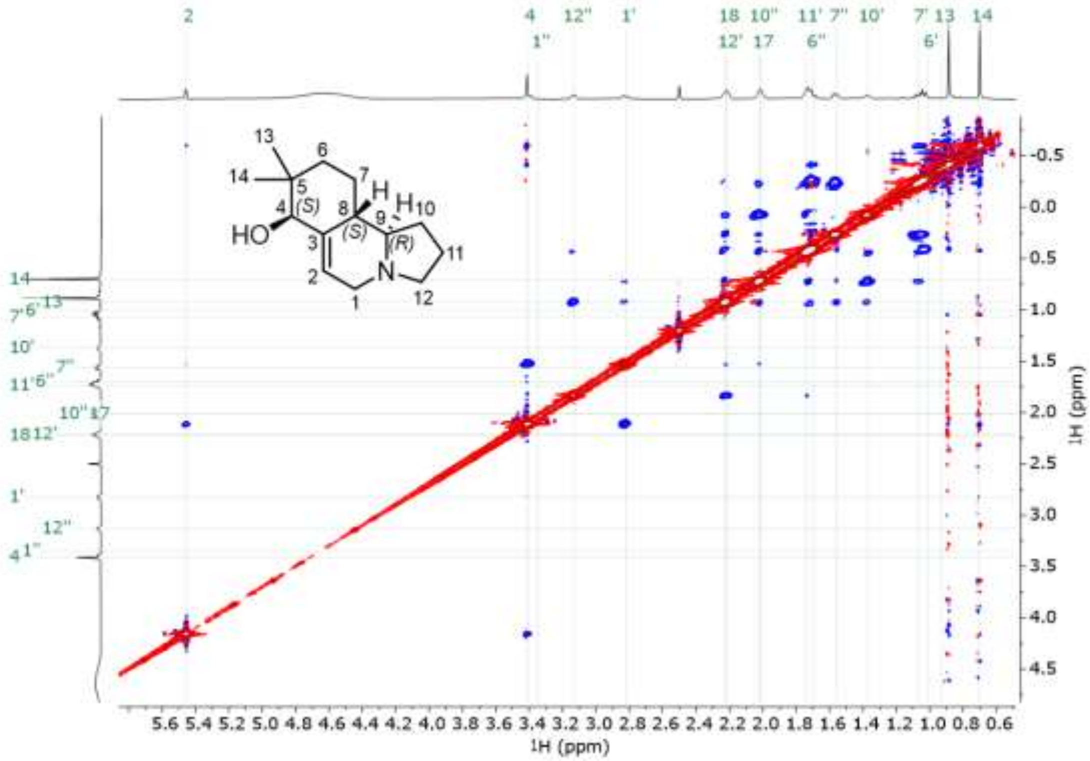

Figure S47. EASY ROESY NMR spectrum of ischnocybine A alcohol (**6**) [600 MHz, DMSO-d_6].

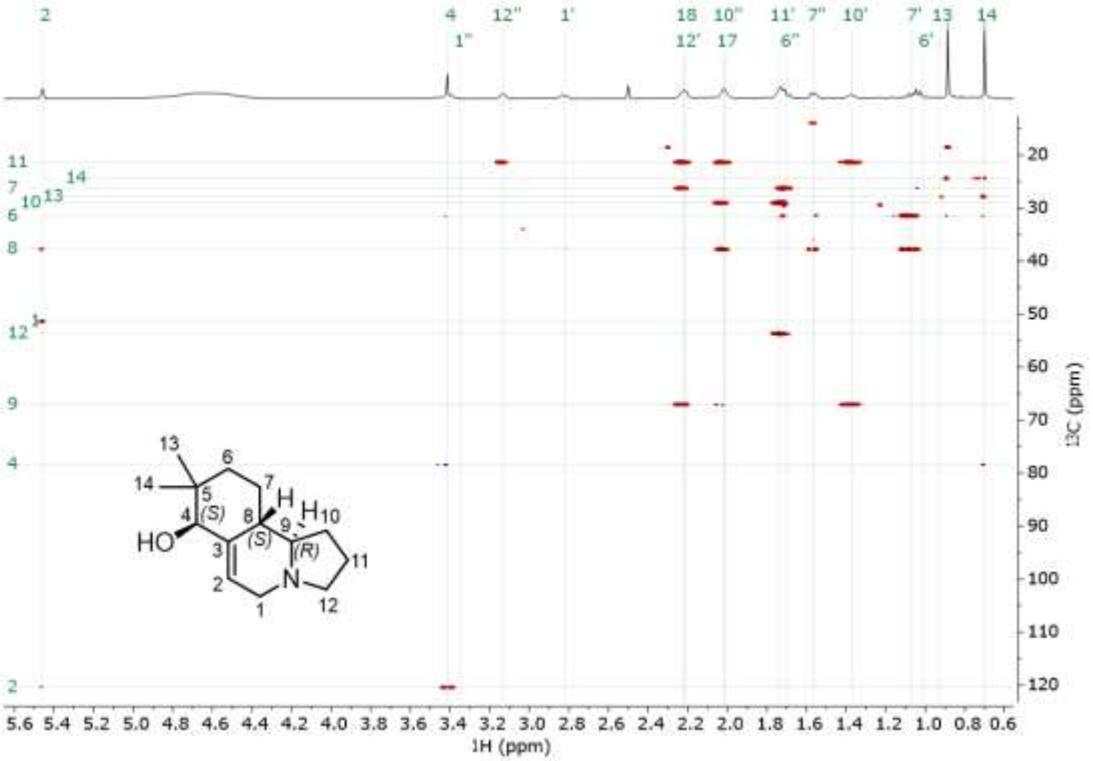

Figure S48. H2BC NMR spectrum of ischnocybine A alcohol (**6**) [600 MHz, DMSO-d_6].

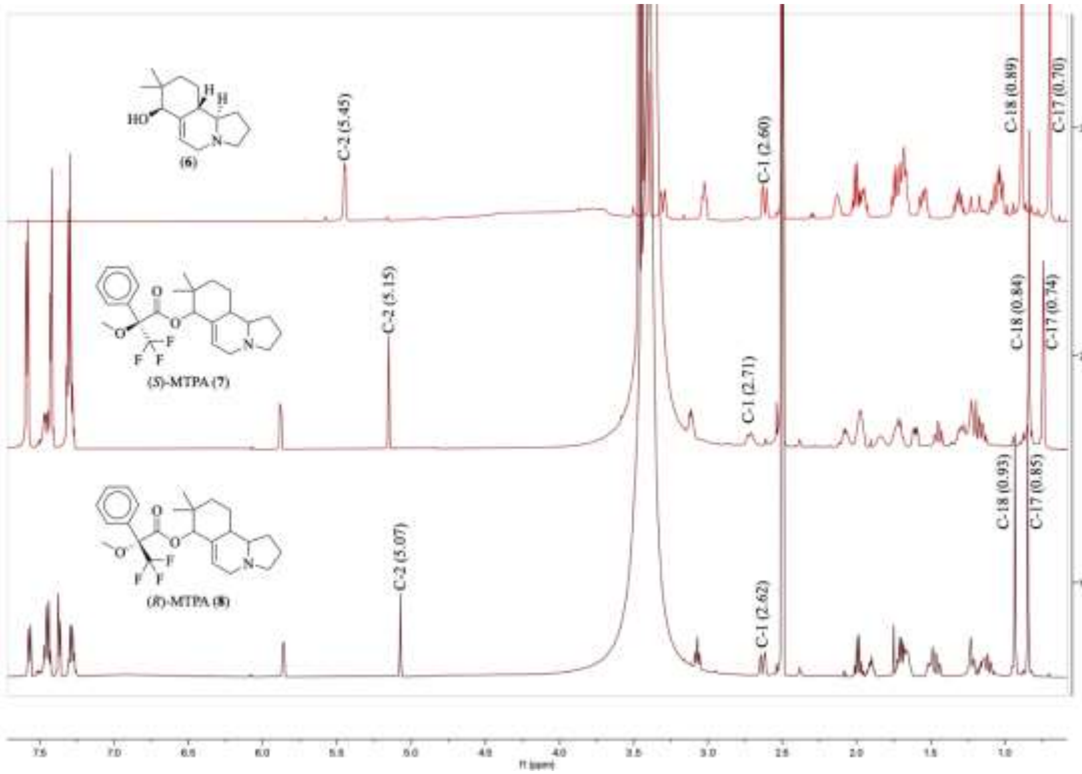

Figure S49. Mosher's analysis of ischnocybine A (**1**)

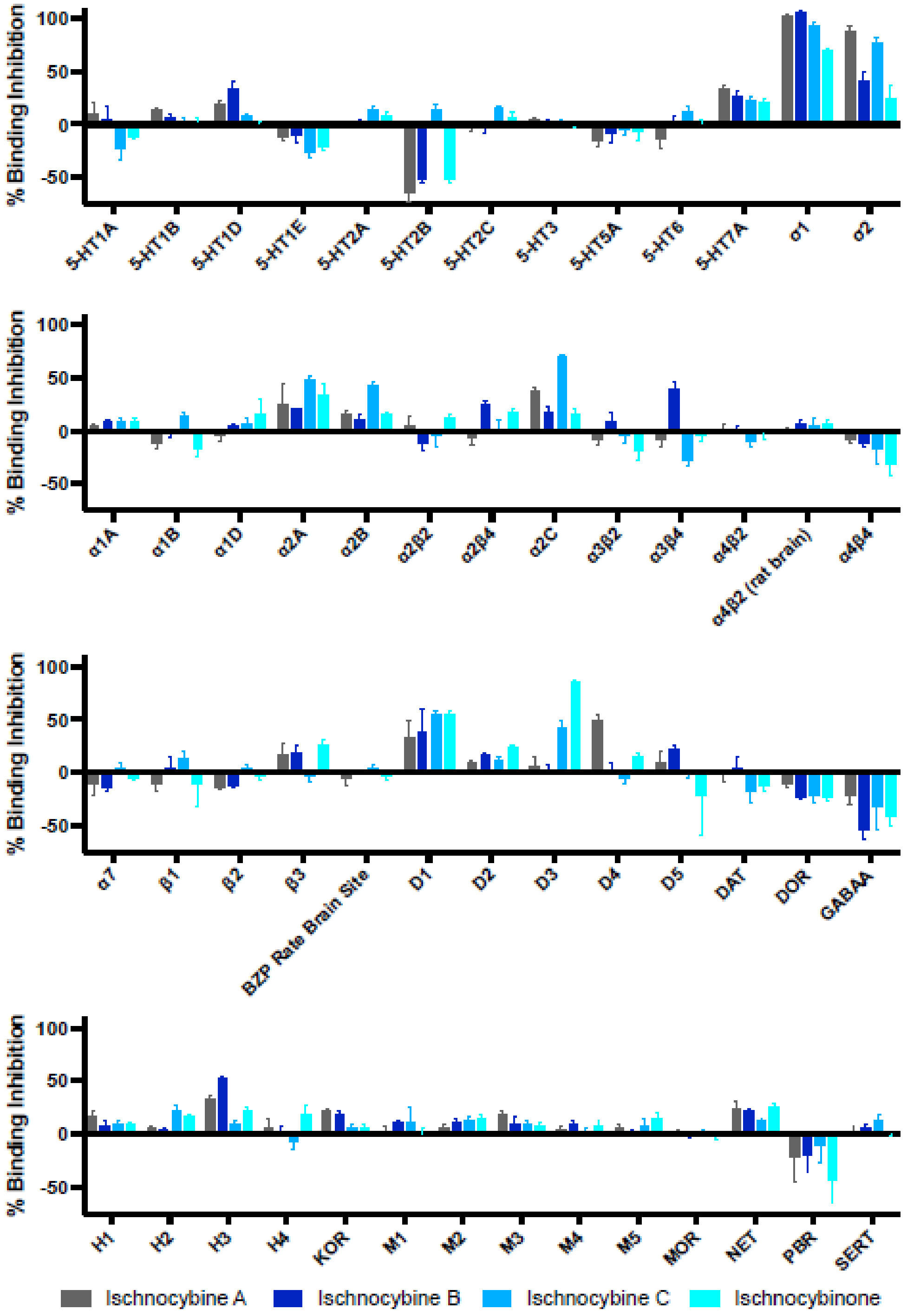

Figure S50. All preliminary PDSP data graphed. Bars represent the mean and error bars represent standard error.

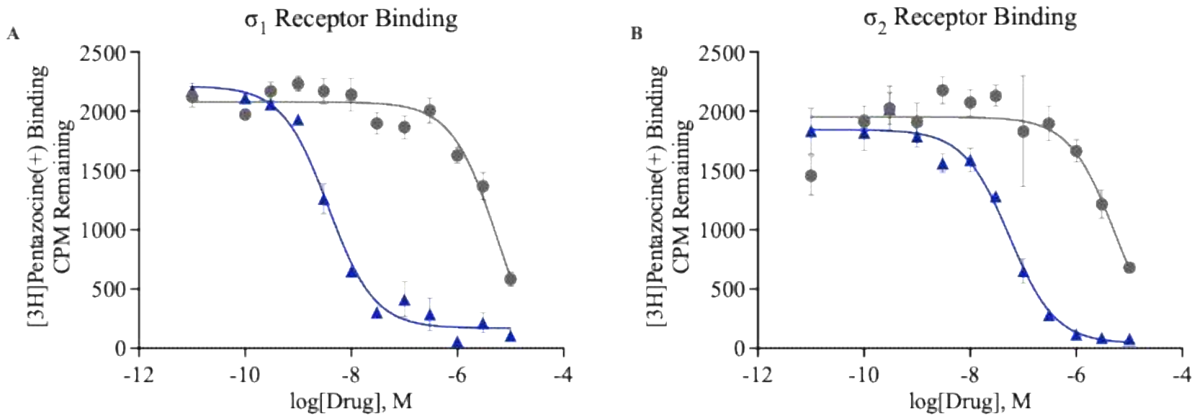

Figure S51. Sigma-1 and Sigma-2 Binding curves for Compound **6**.
Competitive binding curves for **6**, against the radioactive σ_1R ligand, [3H]-(+)-pentazocine. Competitive binding curves for **6**, against the radioactive σ_2R ligand, [3H]-(+)pentazocine. Positive control was haloperidol for both σ_1R and σ_2R (triangles).

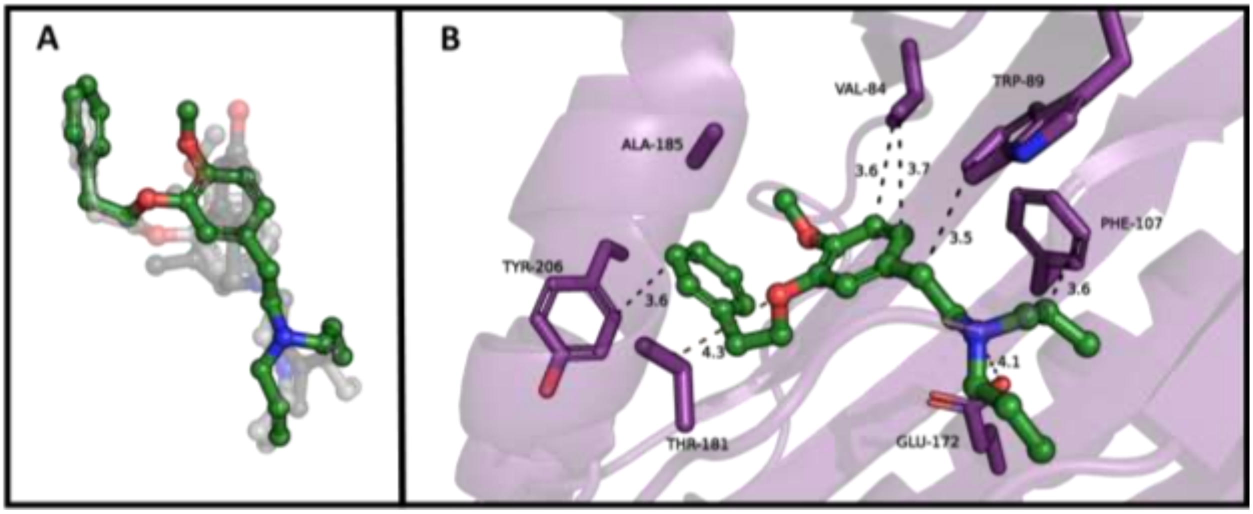

Figure S52. NE-100 docked into the agonist σ_1 receptor

NE-100 docked into the agonist σ_1 receptor (PDB ID: 6DK1). NE-100 binding, shown as green sticks (colored by element), is coordinated by Tyr-206 in the left quadrant of the binding pocket. (A) Overlay of the NE-100 docked pose (green) with the agonist (light grey) and antagonist (dark grey) binding positions from co-crystal structures. (B) Interactions between residues in the σ1 receptor (purple, colored by element) binding cavity and NE-100 (green, colored by element).

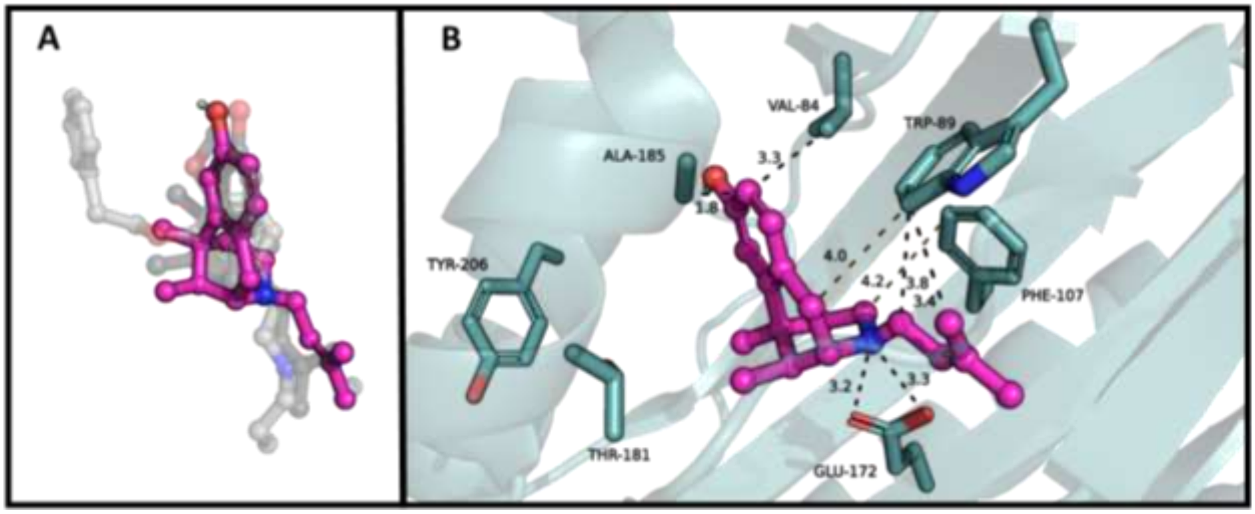

Figure S53. Pentazocine docked into the antagonist σ_1 receptor

Pentazocine docked into the antagonist σ_1 receptor (PDB ID: 6DK0). Pentazocine binding, shown as pink sticks (colored by element), is coordinated through hydrophobic interactions with Ala-185 and Val-84 in the top of the binding pocket. (A) Overlay of pentacozine (pink) with the agonist (light grey) and antagonist (dark grey) binding positions from co-crystal structures. (B) Interactions between residues in the σ1 receptor (teal, colored by element) binding cavity and pentazocine (pink, colored by element).

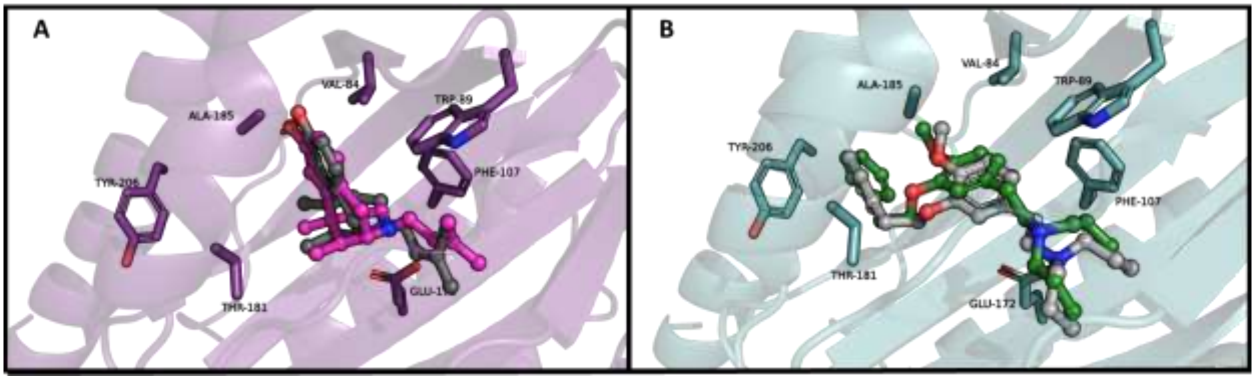

Figure S54. Redocking of pentazocine and NE-100 into agonist and antagonist σ_1 receptor structures.

Redocking of pentazocine and NE-100 into agonist (PDB ID: 6DK1) and antagonist (PDB ID: 6DK0) σ_1 receptor structures. (A) Pentazocine, shown as pink sticks and colored by element, re-docked into agonist structure, shown in purple cartoon, (PDB ID: 6DK1) with an RMSD value of 0.74 Å. (B) NE-100, shown as green sticks and colored by element, re-docked into antagonist structure, shown in teal cartoon, (PDB ID: 6DK0) with an RMSD value of 1.25 Å. Crystal structure ligand positioning is shown as grey sticks in both.

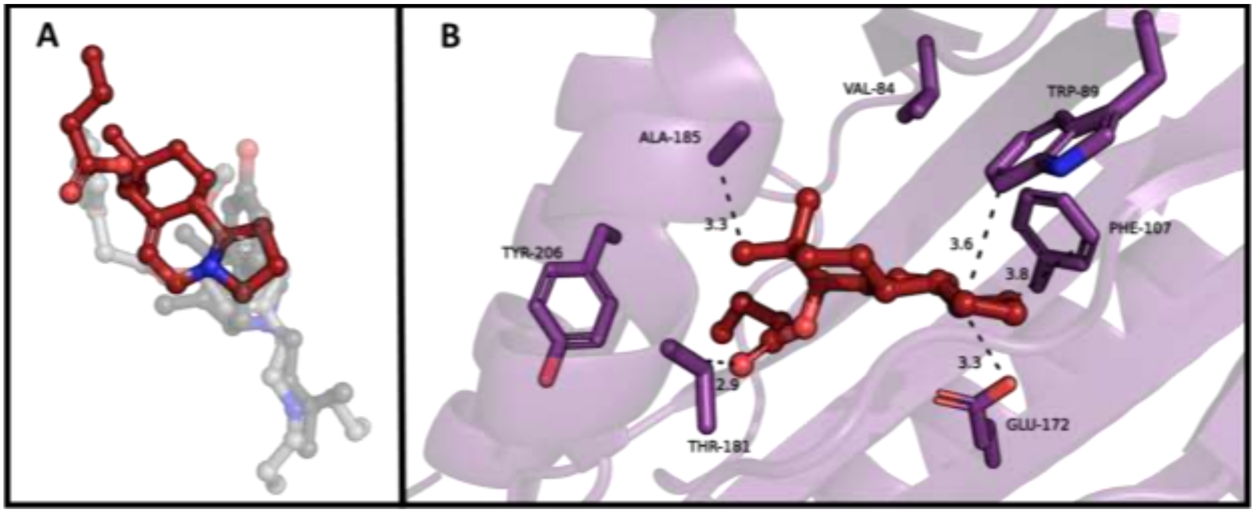

Figure S55. Ischnocybine A-SSR (**1**) docked into the agonist σ_1 receptor

Ischnocybine A-SSR (**1**) docked into the agonist σ_1 receptor (PDB ID: 6DK1). Ischnocybine A-SSR, shown as red sticks (colored by element), mimics binding coordination of A-RRS with Glu-172, Phe-107, Trp-89, and Ala-185 interactions. (A) Overlay of ischnocybine A-SSR (red) with the agonist (light grey) and antagonist binding positions (dark grey) binding positions from co-crystal structures. (B) Interactions between residues in the σ1 receptor (purple, colored by element) binding cavity and ischnocybine A-SSR (red, colored by element).

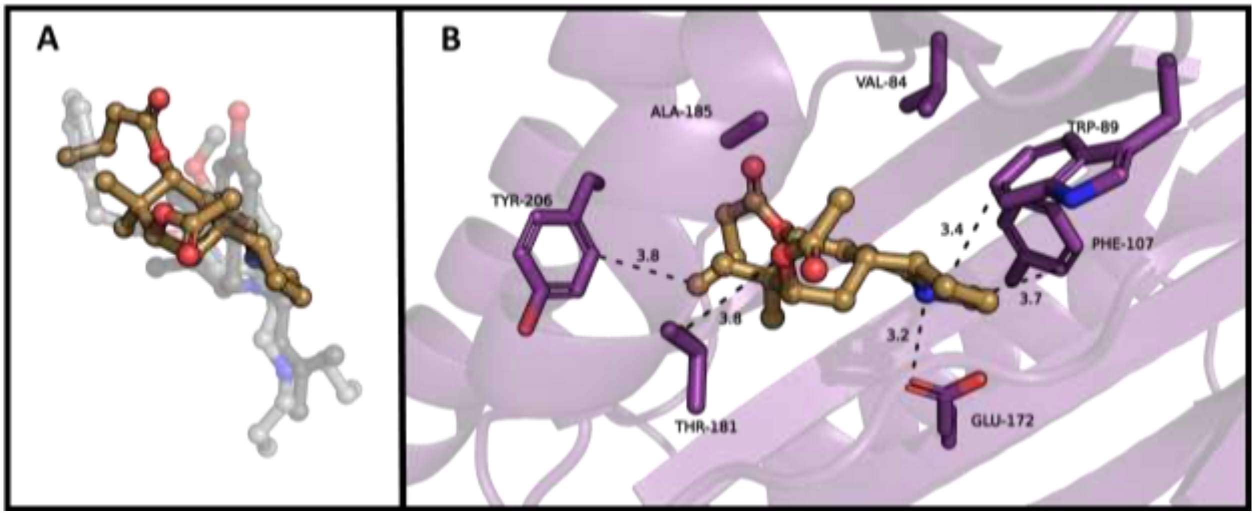

Figure S56. Ischnocybine B-RRSR (**3**) docked into the agonist σ_1 receptor

Ischnocybine B-RRSR (**3**) docked into the agonist σ_1 receptor (PDB ID: 6DK1). Ischnocybine B-RRSR, shown as tan sticks (colored by element), is coordinated by Tyr-206 in the left quadrant of the binding pocket. (A) Overlay of ischnocybine B-RRSR (tan) with the agonist (light grey) and antagonist (dark grey) binding positions from co-crystal structures. (B) Interactions between residues in the σ1 receptor (purple, colored by element) binding cavity and ischnocybine B-RRSR (tan, colored by element).

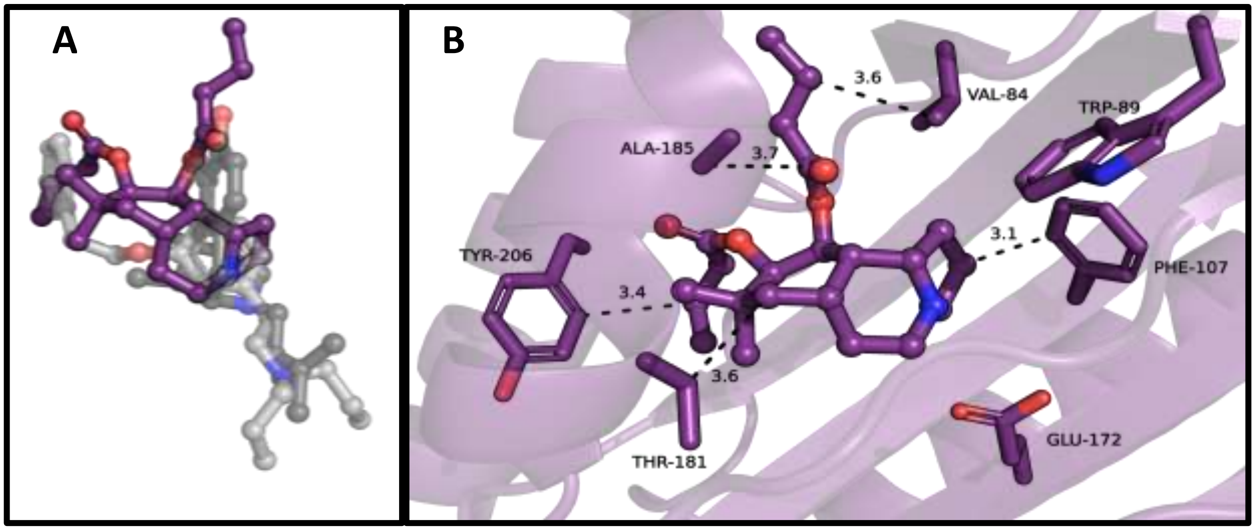

Figure S57. Ischnocybine C-SRSSS (**4**) docked into the agonist σ_1 receptor

Ischnocybine C-SRSSS (**4**) docked into the agonist σ_1 receptor (PDB ID: 6DK1). Ischnocybine C-SRRSS, shown as purple sticks (colored by elements), is coordinated distally by Tyr-206 in the left quadrant of the binding pocket. (A) Overlay of ischnocybine C-SRSSS (purple) with the agonist (light grey) and antagonist (dark grey) binding positions from co-crystal structures. (B) Interactions between residues in the σ1 receptor (purple, colored by element) binding cavity and ischnocybine C-SRSSS (purple, colored by element).

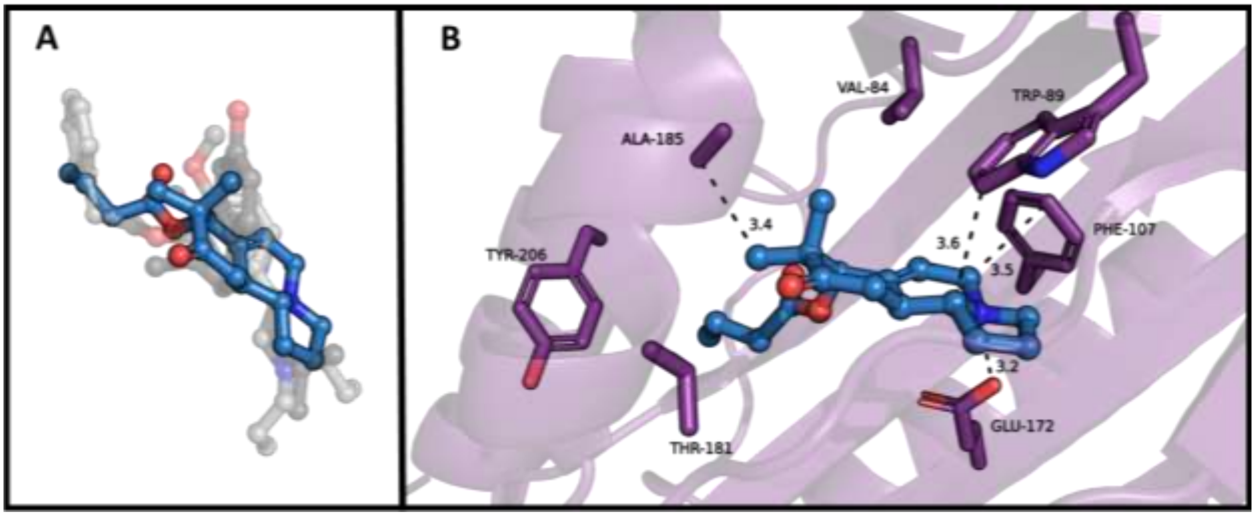

Figure S58. Ischnocybinone-RSR (**2**) docked into the agonist σ_1 receptor

Ischnocybinone-RSR (**2**) docked into the agonist σ_1 receptor (PDB ID: 6DK1). Ischnocybinone-RSR, shown as blue sticks (colored by element), does not interact with Tyr-206 A) Overlay of isc ischnocybinone-RSR (blue) with the agonist (light grey) and antagonist (dark grey) binding positions from co-crystal structures. (B) Interactions between residues in the σ1 receptor (purple, colored by element) binding cavity and ischnocybinone-RSR (blue, colored by element).

2. Supplementary Table

Table S1. NMR Spectroscopy Data (500 Mhz, d_6-DMSO) for Ischnocybine A (**1**)

Atom Number	δ_C, type	δ_H, (*J* in Hz)	HMBC Correlations
**1a**<br>**1b**	52.1, CH_2	3.30, ddd (16.3, 5.0, 2.3)<br>2.59, ddd (16.3, 4.4, 1.7)	C2, C3, C9, C12
**2**	126.3, CH	5.65, dd (4.4, 2.3)	C1, C4, C8
**3**	135.4, C		
**4**	80.1, CH	4.88, d (1.4)	C2, C3, C5, C6, C8, C13, C17, C18
**5**	34.5, C		
**6a**<br>**6b**	32.4, CH_2	1.65, m<br>1.19, m	C5, C7, C8, C17, C18
**7a**<br>**7b**	25.8, CH_2	1.65, m<br>1.14, m	C3, C5, C6, C8
**8**	39.4, CH	1.97, m	
**9**	67.0, CH	1.73, td (9.0, 6.8)	C1
**10a**<br>**10b**	29.4, CH_2	1.97, m<br>1.32, m	C8, C9, C11, C12
**11**	21.6, CH_2	1.65, m	C10
**12a**<br>**12b**	54.2, CH_2	3.00, m<br>1.97, m	C1, C9, C10, C11
**13**	172.2, C		
**14**	36.3, CH_2	2.24, td (7.3, 1.3)	C13, C15, C16
**15**	18.6, CH_2	1.53, qd (7.3, 2.8)	
**16**	13.9, CH_3	0.87, t (7.3)	C14, C15
**17 (ax)**	23.8, CH_3	0.81, s	C4, C5, C6, C18
**18 (eq)**	27.2, CH_3	0.84, s	C4, C5, C6, C17

Table S2. NMR Spectroscoby Data (500 MHz, d_6-DMSO) for Ischnocybinone (**2**)

Atom Number	δ_C, type	δ_H, (*J* in Hz)	HMBC Correlations
**1a**<br>**1b**	51.9, CH_2	3.41, ddd (16.8, 4.9, 2.1)<br>2.74, ddd (16.8, 4.0, 1.7)	C2, C3, C9, C12
**2**	128.1, CH	5.92, dd (4.9, 2.1)	C1, C4, C8
**3**	132.3, C		
**4**	83.6, CH	5.30, s	C2, C3, C5, C6, C8, C17, C18
**5**	48.7, C		
**6**	211.7, C		
**7a**<br>**7b**	40.1, CH_2	2.40, d (11.4)<br>2.31, m	C3, C5, C6, C8, C9
**8**	40.3, CH	2.47, m	
**9**	66.9, CH	1.98, m	C1
**10**	29.0, CH_2	1.93, m<br>1.33, m	C9, C11, C12
**11**	21.5, CH_2	1.69, m	C9, C10, C12
**12a**<br>**12b**	53.9, CH_2	3.03, ddd (8.8, 6.7, 3.6)<br>2.06, q (8.8)	C1, C9, C10, C11
**13**	171.9, C		
**14**	36.0, CH_2	2.21, t (7.4)	C13, C15, C16
**15**	18.4, CH_2	1.48, dtd (14.8, 7.4, 5.9)	C13, C14, C16
**16**	13.8, CH_3	0.84, t (7.4)	C14, C15
**17 (ax)**	24.2, CH_3	1.08, s	C4, C5, C6, C18
**18 (eq)**	20.0, CH_3	0.92, s	C4, C5, C6, C17

Table S3. NMR Spectroscopy Data (500 MHz, d_6-DMSO) for Ischnocybine B (**3**)

**Atom Number**	**δ_C, type**	**δ_H, (*J* in Hz)**	**HMBC Correlations**
**1a**	52.0, CH_2	3.31, m	C2, C3, C9
**1b**		2.63, m	
**2**	127.6, CH	5.74, m	C4, C8
**3**	133.3, C		
**4**	81.4, CH	5.04, s	C2, C3, C5, C6, C8, C13, C20
**5**	38.5, C		
**6**	73.6, CH	5.01, dd (12.1, 4.9)	C5, C17, C20
**7a**	30.7, CH_2	1.81, m	C3, C5, C6, C8, C9
**7b**		1.20, m	
**8**	38.9, CH	2.19, m	
**9**	66.5, CH	1.81, m	C1
**10a**	29.2, CH_2	1.94, m	
**10b**		1.33, m	
**11**	21.6, CH_2	1.67, m	
**12a**	54.1, CH_2	3.00, m	C1, C11
**12b**		2.12, m	
**13**	172.0, C		
**14**	36.2, CH_2	2.28, td (7.4, 2.1)	C13, C15, C16
**15**	18.6, CH_2	1.53, m	C13, C14, C16
**16**	13.9, CH_3	0.87, t (7.4)	C14, C15
**17**	170.6, C		
**18**	21.3, CH_3	2.02, s	C17
**19 (ax)**	18.6, CH_3	0.84, s	C4, C5, C6, C20
**20 (eq)**	23.0, CH_3	0.84, s	C4, C5, C6, C19

Table S4. NMR Spectroscopy Data (500 MHz, d_6-DMSO) for Ischnocybine C (**4**)

Atom Number	δ_C, type	δ_H, (*J* in Hz)	HMBC Correlations
**1a**	54.1, CH_2	3.00, m	C9
**1b**		1.81, m	
**2a**	29.3, CH_2	1.86, m	C8
**2b**		1.35, m	
**3**	36.7, CH	1.86, m	
**4a**	39.0, CH_2	1.70, dd (14.2, 4.9)	C5, C6, C8, C21, C22
**4b**		1.26, d (14.2)	
**5**	35.0, C		
**6**	76.5, CH	4.87, d (2.7)	C4, C5, C7, C8, C13, C21
**7**	69.3, CH	5.41, dd (11.6, 2.7)	C6, C8, C9, C17
**8**	35.8, CH	2.25, m	C7
**9**	66.9, CH	2.04, m	
**10a**	27.7, CH_2	1.77, m	C9, C11
**10b**		1.63, ddd (13.7, 9.2, 5.3)	
**11a**	21.7, CH_2	1.52, m	
**11b**		1.44, m	
**12a**	54.3, CH_2	2.91, td (8.5, 2.5)	C9, C10
**12b**		1.90, d (8.5)	
**13**	172.9, C		
**14**	36.1, CH_2	2.30, td (7.3, 1.5)	C13, C15, C16
**15**	18.6, CH_2	1.57, qd (7.3, 3.6)	C13, C14, C16
**16**	13.8, CH_3	0.91, t (7.3)	C14, C15
**17**	172.4, C		
**18**	36.2, CH_2	2.09, td (7.4, 2.6)	C17, C19, C20
**19**	17.9, CH_2	1.48, q (7.4)	C17, C18, C20
**20**	13.9, CH_3	0.84, t (7.4)	C18, C19
**21 (ax)**	29.3, CH_3	0.77, s	C4, C5, C6, C22
**22 (eq)**	27.9, CH_3	1.10, s	C4, C5, C6, C21

Table S5. ^{13}C NMR Chemical Shifts Predictions for Ischnocybine A (**1**)

**Gaussian Numbering**[a]	**Experimental**	**srr**	**srs**	**ssr**	**sss**
1	34.0	38.0	36.6	35.1	36.8
2	80.1	81.8	79.9	81.7	83.2
3	135.4	139.5	137.7	137.1	139.1
4	39.4	41.4	43.8	40.4	37.0
5	25.8	24.0	26.2	25.8	23.8
6	32.4	38.1	37.0	32.8	34.4
7	27.2	29.1	28.9	28.0	27.9
8	23.8	20.3	20.2	24.4	25.1
9	126.3	117.4	118.5	128.4	125.1
10	52.1	51.2	51.0	50.9	51.3
12	67.0	62.4	66.3	66.1	62.5
13	54.2	54.5	54.3	54.4	54.4
14	21.6	23.6	22.9	22.8	23.7
15	29.4	24.7	29.7	29.6	24.5
17	172.2	169.4	169.5	169.1	169.3
**RMSD**		3.9	3.1	1.3	2.7
**MAE**		3.3	2.3	1.1	2.3

[a]*Note the Gaussian atom numberings do not match the experimental literature numberings. Experimental chemical shifts have been reordered accordingly. Configurations are presented in the form "rrs" corresponding to numerical order of the literature numbering for stereopositions. Example, ischnocybine A's "ssr" corresponds to the literature assignment "4S,8S,9R."*

Table S6. ^{1}H NMR Chemical Shift Predictions for Ischnocybine A (**1**)

**Gaussian Numbering**[a]	**Experimental**	**srr**	**srs**	**ssr**	**sss**
20	1.97	1.99	1.96	2.04	2.21
21	1.73	2.45	2.02	1.99	2.40
22	4.88	4.96	5.18	4.94	4.82
23 (24)	1.65	1.52	1.47	1.52	1.56
24 (23)	1.14	1.36	1.12	1.15	1.40
25 (26)	1.65	1.46	1.43	1.69	1.68
26 (25)	1.19	1.37	1.40	1.21	1.28
27,28,29	0.84	0.89	0.86	0.86	0.90
30,31,32	0.81	0.79	0.80	0.76	0.78
33	5.65	5.33	5.37	5.73	5.63
34 (35)	3.30	3.19	3.15	3.18	3.18
35 (34)	2.59	2.76	2.79	2.80	2.79
36 (37)	3.00	2.80	2.81	2.82	2.79
37 (36)	1.97	2.14	2.14	2.14	2.13
38 (39)	1.65	1.60	1.66	1.65	1.59
39 (38)	1.65	1.57	1.60	1.60	1.57
40 (41)	1.97	1.65	1.83	1.83	1.66
41 (40)	1.32	1.58	1.35	1.34	1.56
**RMSD**		0.24	0.17	0.12	0.22
**MAE**		0.18	0.14	0.09	0.16

[a]Note the Gaussian atom numberings do not match the experimental literature numberings. Experimental chemical shifts have been reordered accordingly. Configurations are presented in the form "rrs" corresponding to numerical order of the literature numbering for stereopositions. Example, ischnocybine A's "ssr" corresponds to the literature assignment "4S,8S,9R."

Table S7. ^{13}C NMR Chemical Shifts Predictions for Ischnocybinone (**2**)

**Gaussian Numbering**[a]	**Experimental**	**rrr**	**rrs**	**rsr**	**srr**
1	48.7	53.0	51.3	49.6	51.2
2	83.6	78.8	77.3	84.9	84.0
3	132.3	136.7	134.5	133.5	136.1
4	40.3	36.9	40.6	41.5	36.9
5	40.1	39.9	40.7	40.1	39.5
6	211.7	215.3	214.7	216.0	217.0
7	24.2	22.3	22.3	24.7	25.5
8	20.0	21.2	21.5	21.8	21.8
9	128.1	121.1	123.0	130.8	128.6
10	51.9	50.8	50.8	50.6	51.0
12	66.9	61.6	66.2	66.0	62.0
13	53.9	53.9	54.2	54.1	53.9
14	21.5	23.9	22.8	22.7	23.9
15	29.0	24.7	29.3	29.2	24.5
17	171.9	169.2	169.1	168.6	168.8
**RMSD**		3.7	2.6	1.8	2.9
**MAE**		3.1	2.0	1.4	2.4

[a]*Note the Gaussian atom numberings do not match the experimental literature numberings. Experimental chemical shifts have been reordered accordingly. Configurations are presented in the form "rrs" corresponding to numerical order of the literature numbering for stereopositions. Example, ischnocybine A's "ssr" corresponds to the literature assignment "4S,8S,9R."*

Table S8. ^{1}H NMR Chemical Shift Predictions for Ischnocybinone (**2**)

Gaussian Numbering[a]	Experimental	rrr	rrs	rsr	srr
20	2.47	2.21	2.24	2.50	2.62
21	1.98	2.65	2.19	2.23	2.56
23	5.30	5.11	5.35	5.31	5.15
24 (25)	2.40	2.72	2.36	2.34	2.69
25 (24)	2.31	2.33	2.26	2.26	2.34
26,27,28	1.08	1.05	1.09	1.08	1.04
29,30,31	0.92	1.04	1.00	1.00	1.03
32	5.92	5.68	5.75	5.97	5.95
33 (34)	3.41	3.32	3.28	3.29	3.34
34 (33)	2.74	2.93	2.93	2.96	2.94
35 (36)	3.03	2.84	2.85	2.85	2.85
36 (35)	2.06	2.25	2.22	2.24	2.23
37 (38)	1.69	1.61	1.69	1.67	1.62
38 (37)	1.69	1.58	1.63	1.62	1.60
39 (40)	1.93	1.64	1.84	1.84	1.61
40 (39)	1.33	1.55	1.36	1.34	1.55
**RMSD**		0.25	0.13	0.12	0.22
**MAE**		0.20	0.11	0.09	0.17

[a]*Note the Gaussian atom numberings do not match the experimental literature numberings. Experimental chemical shifts have been reordered accordingly. Configurations are presented in the form "rrs" corresponding to numerical order of the literature numbering for stereopositions. Example, ischnocybine A's "ssr" corresponds to the literature assignment "4S,8S,9R."*

Table S9. ^{13}C NMR Chemical Shifts Predictions for Ischnocybine B (**3**)

**Gaussian Numbering**[a]	**Experimental**	**rrrr**	**rrrs**	**rrsr**	**rsrr**	**rsrs**	**rssr**	**srrr**	**ssrr**
1	38.5	41.8	40.3	39.4	42.4	41.1	38.2	40.3	40.7
2	81.4	78.2	76.1	82.6	79.4	78.3	79.8	81.8	83.0
3	133.3	138.6	136.6	134.8	137.5	134.1	135.9	138.1	137.0
4	38.9	36.4	39.6	39.6	37.0	30.2	36.7	32.9	35.5
5	30.7	28.2	29.7	30.4	28.1	31.3	30.6	29.2	27.9
6	73.6	79.5	78.8	74.4	78.2	77.4	76.1	76.9	77.1
7	18.6	24.3	24.2	19.6	25.6	25.2	25.3	23.4	24.5
8	23.0	20.2	20.6	24.2	15.9	16.1	23.7	25.6	20.4
9	127.6	118.6	119.4	129.9	119.2	118.1	130.1	126.6	127.0
10	52.0	51.1	50.8	50.7	51.0	45.0	50.8	51.1	51.1
12	66.5	61.9	65.7	65.6	61.8	62.0	65.5	61.9	62.0
13	54.1	54.3	54.2	54.2	54.3	48.7	54.3	54.2	54.2
14	21.6	23.7	22.9	22.7	23.6	22.5	22.8	23.9	23.7
15	29.2	24.6	29.5	29.4	24.7	30.5	29.5	24.4	24.5
17	172.0	169.4	169.5	168.9	169.3	169.3	169.2	169.4	169.1
23	170.6	169.2	169.4	169.8	169.7	169.7	169.6	169.5	169.8
24	21.3	21.0	21.0	21.0	20.9	20.9	21.3	21.3	21.0
**RMSD**		4.0	3.3	1.3	4.2	4.8	2.2	3.1	3.0
**MAE**		3.4	2.4	1.0	3.4	3.9	1.6	2.5	2.5

[a]*Note the Gaussian atom numberings do not match the experimental literature numberings. Experimental chemical shifts have been reordered accordingly. Configurations are presented in the form "rrs" corresponding to numerical order of the literature numbering for stereopositions. Example, ischnocybine A's "ssr" corresponds to the literature assignment "4S,8S,9R."*

Table S10. ^{1}H NMR Chemical Shift Predictions for Ischnocybine B (**3**)

**Gaussian Numbering**[a]	**Experimental**	**rrrr**	**rrrs**	**rrsr**	**rsrr**	**rsrs**	**rssr**	**srrr**	**ssrr**
20	2.19	2.31	2.19	2.25	2.05	1.91	2.29	2.52	2.36
21	1.81	2.50	2.04	2.07	2.51	2.72	2.00	2.48	2.46
26	5.04	5.40	5.67	5.06	4.96	5.03	5.09	4.93	4.91
27 (28)	1.81	1.91	1.89	1.79	1.99	1.90	1.81	1.83	2.01
28 (27)	1.20	1.61	1.33	1.24	1.38	1.06	1.35	1.69	1.40
29	5.01	4.77	4.70	5.26	4.63	4.62	4.79	4.95	5.00
30,31,32	0.84	0.99	0.94	0.85	0.97	0.96	0.85	0.94	0.98
33,34,35	0.84	0.87	0.86	0.91	0.91	0.93	0.94	0.87	0.88
36	5.74	5.41	5.44	5.81	5.46	5.42	5.83	5.74	5.76
37 (38)	3.31	3.19	3.17	3.21	3.21	3.40	3.21	3.21	3.20
38 (37)	2.63	2.77	2.81	2.86	2.80	3.00	2.85	2.84	2.81
39 (40)	3.00	2.79	2.82	2.81	2.80	2.64	2.83	2.80	2.82
40 (39)	2.12	2.14	2.16	2.18	2.16	2.62	2.17	2.16	2.14
41 (42)	1.67	1.58	1.66	1.66	1.60	1.77	1.65	1.59	1.59
42 (41)	1.67	1.55	1.60	1.60	1.56	1.72	1.59	1.57	1.55
43 (44)	1.94	1.55	1.79	1.81	1.61	1.75	1.81	1.58	1.61
44 (43)	1.33	1.53	1.28	1.35	1.59	1.58	1.30	1.57	1.60
48,49,50	2.02	2.06	2.07	2.03	2.03	2.04	2.09	2.09	2.03
**RMSD**		0.27	0.21	0.12	0.25	0.32	0.12	0.25	0.22
**MAE**		0.21	0.15	0.09	0.19	0.24	0.10	0.18	0.16

[a]*Note the Gaussian atom numberings do not match the experimental literature numberings. Experimental chemical shifts have been reordered accordingly. Configurations are presented in the form "rrs" corresponding to numerical order of the literature numbering for stereopositions. Example, ischnocybine A's "ssr" corresponds to the literature assignment "4S,8S,9R."*

Table S11. ^{13}C NMR Chemical Shifts Predictions for Ischnocybine C (**4**)

**Gaussian Numbering**[a]	**Experimental**	**rrrrr**	**rrrrs**	**rrrss**	**rrsss**	**rsrss**	**rssrs**	**rsssr**	**rssss**
1	76.5	80.7	74.6	79.7	83.7	78.8	74.5	80.7	81.0
2	69.3	75.8	72.0	74.7	78.2	71.1	74.6	78.7	77.4
3	35.8	43.2	44.9	39.4	40.4	43.2	43.5	49.7	45.5
4	36.7	37.1	27.0	26.3	29.1	26.4	29.5	34.7	28.8
5	39.0	41.6	34.8	41.2	39.4	45.4	37.7	43.7	43.8
6	35.0	36.7	34.3	34.9	36.8	35.3	36.0	36.5	36.7
7	66.9	66.2	59.2	62.7	59.4	62.7	58.0	66.4	59.8
9	54.1	51.7	47.4	46.6	46.9	46.6	46.5	50.6	46.7
10	29.3	27.9	30.9	33.5	32.6	33.0	30.3	32.3	32.5
11	27.7	26.5	29.4	24.9	22.4	24.4	32.2	31.1	22.7
12	21.7	24.8	21.9	22.6	22.3	22.5	21.8	22.3	22.3
13	54.3	54.1	54.6	54.1	54.7	53.9	53.2	53.5	54.7
14	27.9	31.9	25.5	25.7	24.6	22.8	21.2	21.2	21.1
15	29.3	25.7	28.8	29.1	28.2	30.8	29.7	29.6	29.5
18	172.9	170.6	169.2	170.4	169.9	170.3	169.4	169.9	170.1
20	172.4	169.6	169.0	169.6	168.8	170.2	170.1	169.2	169.2
**RMSD**		3.4	4.7	4.2	5.0	4.7	4.8	5.2	5.4
**MAE**		2.8	3.5	3.3	4.1	3.7	3.8	3.8	4.6

[a]*Note the Gaussian atom numberings do not match the experimental literature numberings. Experimental chemical shifts have been reordered accordingly. Configurations are presented in the form "rrs" corresponding to numerical order of the literature numbering for stereopositions. Example, ischnocybine A's "ssr" corresponds to the literature assignment "4S,8S,9R."*

Table S12. ^{13}C NMR Chemical Shifts Predictions for Ischnocybine C (**4**) (cont.)

Gaussian Numbering[a]	Experimental	srrss	srsrs	srssr	srsss	ssrrs	ssrsr	ssrss	sssrs
1	76.5	78.3	84.0	80.3	76.9	81.6	76.3	79.3	81.4
2	69.3	74.0	77.2	71.7	71.3	75.4	76.7	68.0	76.7
3	35.8	34.7	49.2	46.9	37.0	44.6	45.3	38.2	44.9
4	36.7	34.8	31.1	26.6	36.9	34.9	30.2	34.7	31.0
5	39.0	38.9	44.6	38.2	39.1	39.6	34.5	42.3	40.7
6	35.0	33.6	36.7	36.4	35.3	36.4	35.3	34.5	35.8
7	66.9	65.0	63.2	58.7	65.9	66.4	59.6	64.9	62.9
9	54.1	52.4	50.7	46.7	51.7	50.8	47.3	52.2	50.7
10	29.3	29.7	32.4	30.1	28.7	32.7	31.0	29.8	32.9
11	27.7	26.4	28.8	29.8	26.8	31.6	32.4	26.1	28.8
12	21.7	21.7	22.2	21.9	24.6	22.2	21.9	21.8	22.2
13	54.3	54.3	54.0	54.3	53.9	53.5	53.6	54.4	54.0
14	27.9	28.2	30.3	30.7	28.7	28.1	28.2	24.6	28.5
15	29.3	30.7	22.5	22.0	29.9	24.7	25.3	32.3	25.4
18	172.9	169.0	169.1	169.9	169.9	170.1	168.5	170.5	168.0
20	172.4	170.5	169.8	169.0	170.6	169.1	169.4	170.2	168.3
**RMSD**		2.0	5.4	5.3	1.5	3.7	4.9	2.1	4.3
**MAE**		1.5	4.3	4.0	1.2	2.9	3.8	1.8	3.5

[a]*Note the Gaussian atom numberings do not match the experimental literature numberings. Experimental chemical shifts have been reordered accordingly. Configurations are presented in the form "rrs" corresponding to numerical order of the literature numbering for stereopositions. Example, ischnocybine A's "ssr" corresponds to the literature assignment "4S,8S,9R."*

Table S13. ^{1}H NMR Chemical Shift Predictions for Ischnocybine C (**4**)

Gaussian Numbering[a]	Experimental	rrrrr	rrrrs	rrrss	rrsss	rsrss	rssrs	rsssr	rssss
24	2.25	1.91	1.64	2.07	2.26	1.83	2.05	1.31	1.88
25	2.04	2.30	2.46	2.78	2.91	2.76	2.30	2.00	2.97
26	1.86	1.73	2.03	1.76	1.53	1.76	1.97	1.36	1.55
27	4.87	5.01	4.81	4.20	4.28	4.56	5.17	4.86	4.89
28	5.41	5.08	4.76	4.73	4.47	5.34	4.91	4.41	4.38
29 (30)	1.70	1.53	1.76	1.25	1.36	1.47	1.69	1.36	1.37
30 (29)	1.26	1.39	0.91	1.25	1.16	1.06	1.04	1.17	1.16
31 (32)	3.00	2.84	2.57	2.42	2.40	2.38	2.52	2.77	2.39
32 (31)	1.81	2.06	2.23	2.30	2.37	2.30	2.22	2.09	2.37
33 (34)	1.86	1.89	1.68	1.47	1.39	1.44	1.65	1.44	1.38
34 (33)	1.35	1.26	1.35	1.18	1.22	1.15	1.33	1.24	1.17
35 (36)	1.77	2.01	1.61	1.83	1.69	1.80	1.87	1.48	1.71
36 (35)	1.63	1.58	1.33	1.32	1.32	1.16	1.04	1.44	1.36
37 (38)	1.52	1.46	1.61	1.87	1.93	1.84	1.56	1.59	1.93
38 (37)	1.44	1.35	1.53	1.49	1.53	1.46	1.50	1.47	1.54
39 (40)	2.91	2.73	2.75	2.60	2.66	2.58	2.73	2.73	2.67
40 (39)	1.90	2.16	2.20	2.60	2.66	2.58	2.17	2.12	2.66
41,42,43	1.10	1.06	1.03	1.00	0.99	1.07	0.96	0.94	0.93
44,45,46	0.77	0.85	0.81	0.85	0.93	0.86	0.86	0.81	0.82
**RMSD**		0.18	0.31	0.42	0.46	0.37	0.28	0.39	0.47
**MAE**		0.16	0.24	0.34	0.37	0.30	0.22	0.27	0.37

[a]*Note the Gaussian atom numberings do not match the experimental literature numberings. Experimental chemical shifts have been reordered accordingly. Configurations are presented in the form "rrs" corresponding to numerical order of the literature numbering for stereopositions. Example, ischnocybine A's "ssr" corresponds to the literature assignment "4S,8S,9R."*

Table S14. ^{1}H NMR Chemical Shift Predictions for Ischnocybine C (**4**) (cont.)

Gaussian Numbering[a]	Experimental	srrss	srsrs	srssr	srsss	ssrrs	ssrsr	ssrss	sssrs
24	2.25	1.88	1.18	1.76	2.18	1.61	1.89	1.78	1.43
25	2.04	2.23	2.04	2.13	2.26	1.88	2.69	2.17	1.99
26	1.86	1.66	1.66	2.08	1.76	1.35	1.91	1.62	1.61
27	4.87	4.54	4.16	4.18	5.10	4.47	5.07	4.58	4.21
28	5.41	4.64	4.60	5.19	5.54	4.83	4.62	5.59	4.43
29 (30)	1.70	1.73	1.48	1.66	1.66	1.34	1.77	1.57	1.27
30 (29)	1.26	1.36	1.12	1.14	1.20	1.12	0.88	1.54	1.19
31 (32)	3.00	2.79	2.74	2.55	2.80	2.76	2.56	2.78	2.75
32 (31)	1.81	1.90	2.07	2.24	2.06	2.09	2.24	1.93	2.08
33 (34)	1.86	2.18	1.46	1.66	1.77	1.40	1.71	2.18	1.45
34 (33)	1.35	1.22	1.18	1.35	1.21	1.28	1.38	1.25	1.21
35 (36)	1.77	1.55	1.69	1.80	2.12	1.61	1.83	1.44	1.61
36 (35)	1.63	1.43	1.37	1.43	1.55	1.48	1.08	1.42	1.32
37 (38)	1.52	1.57	1.66	1.65	1.45	1.62	1.56	1.48	1.66
38 (37)	1.44	1.53	1.54	1.56	1.40	1.49	1.50	1.48	1.54
39 (40)	2.91	2.68	2.75	2.76	2.66	2.72	2.73	2.69	2.75
40 (39)	1.90	1.99	2.17	2.22	2.14	2.12	2.17	1.96	2.16
41,42,43	1.10	1.13	1.04	1.09	1.04	0.99	0.98	1.16	1.05
44,45,46	0.77	0.87	0.91	0.91	0.83	0.89	0.86	0.86	0.84
**RMSD**		0.26	0.39	0.28	0.17	0.31	0.34	0.22	0.39
**MAE**		0.20	0.29	0.21	0.14	0.26	0.26	0.18	0.29

[a]*Note the Gaussian atom numberings do not match the experimental literature numberings. Experimental chemical shifts have been reordered accordingly. Configurations are presented in the form "rrs" corresponding to numerical order of the literature numbering for stereopositions. Example, ischnocybine A's "ssr" corresponds to the literature assignment "4S,8S,9R."*

Table S15. NMR Spectroscopy Data (600 MHz, d_6-DMSO) for Ischnocybine A alcohol (**6**)

Atom Number	δ_C, type	δ_H, (J in Hz)	HMBC Correlations	H2BC
**1a**	50.8, CH_2	3.39, m		
**1b**		2.82, m		
**2**	119.8, CH	5.46, m	C1, C4, C8	
**3**	140.5, C			
**4**	77.8, CH	3.41, m	C2, C3, C5, C6, C8, C9, C13, C14	
**5**	34.7, C			
**6a**	30.9, CH_2	1.03, m	C4, C5, C8, C14	C7
**6b**		1.71, m		
**7a**	25.7, CH_2	1.07, m	C3, C5, C8. C9	C6, C8
**7b**		1.56, m		
**8**	37.2, CH	2.22, m		C7, C9
**9**	66.4, CH	2.01, m		C8
**10a**	28.4, CH_2	1.37, m	C9	C9, C11
**10b**		2.01, m		
**11**	20.8, CH_2	1.73, m	C10	C10, C12
**12a**	53.1, CH_2	2.21, m	C1, C9, C11	C11
**12b**		3.13, m		
**13 (ax)**	27.2, CH_3	0.89, s	C5, C6, C14	
**14 (eq)**	23.8, CH_3	0.70, s	C5, C6, C13	

Table S16. Isolated quantity of ischnocybines from 300 individual *I. plicata*

Compound	Isolated yields (mg)
Ischnocybine A (**1**)	3.5
Ischnocybinone (**2**)	4.6
Ischnocybine B (**3**)	2.5
Ischnocybine C (**4**)	4.1

Table S17. *I. plicata* length versus alkaloid study

Length (cm)	Ischnocybinone Peak Area	Ischnocybine C Peak Area	Ischnocybine A Peak Area	Ischnocybine C Peak Area	Average Peak Area
0.3	17643	1845	65666	18470	25906
0.2	25562	3669	335116	91752	114025
0.1	35251	6050	317308	107547	116539
0.5	97040	28981	183358	211402	130195
0.3	48442	14202	293137	166698	130620
0.4	42886	13925	397477	209577	165966
0.3	64807	17198	554713	278114	228708
0.3	106927	27558	893830	476669	376246
0.3	150911	52818	921607	563535	422218
0.4	262687	81577	1291386	841654	619326
0.3	640630	172967	1339290	1131484	821093
1.1	1793878	681272	1837761	2252718	1641407
1.0	1417019	487994	2047647	2789888	1685637
0.6	2175818	896569	2523770	2627643	2055950
0.6	1617217	537863	3563506	3231879	2237616
0.9	2636720	917146	4109711	4106126	2942425
0.9	3048933	1494081	3009570	4573806	3031597
0.9	1439746	2197928	3362976	5285957	3071652
1.3	3174492	1249279	3551800	4365751	3085330
0.9	3183205	1161250	4758721	4831573	3483687
1.2	3366300	1114064	4380496	5272009	3533217
1.1	4607372	2022401	3392624	4891568	3728491
1.4	4082932	1557559	4574199	5084344	3824759
1.1	4195985	1295262	5165187	5917442	4143469
0.4	4177802	1440438	5654428	5870414	4285770
1.4	4150454	2105331	5024151	6879529	4539866
1.8	3754321	1775874	5290289	7684069	4626138
1.5	4412749	1629031	6477966	6706466	4806553
1.4	4416033	2020855	5862204	6980630	4819930
2.0	4057982	1894127	7815310	9282077	5762374

Table S18. Primary binding assay (Inhibition %). The Psychoactive Drug Screening Program performed each assay in quadruplet.

Compound	Receptor	Inhibition 1	Inhibition 2	Inhibition 3	Inhibition 4	Mean %
**1**	5-HT1A	-0.79	21.15	33.02	-14.41	9.74
**1**	5-HT1B	14.19	19.75	7.77	10.19	12.98
**1**	5-HT1D	3.74	19.49	27.99	21.21	18.11
**1**	5-HT1E	-13.61	-6.19	-17.44	-12.97	-12.55
**1**	5-HT2A	-4.54	5.00	2.41	0.90	0.94
**1**	5-HT2B	-56.67	-91.23	-58.64	-51.07	-64.4
**1**	5-HT2C	0	6.15	-7.56	-11.09	-3.13
**1**	5-HT3	-3.26	8.08	9.27	1.89	4.00
**1**	5-HT5A	-22.00	-26.13	-8.94	-2.22	-14.82
**1**	5-HT6	-5.81	-0.82	-37.55	-12.38	-14.14
**1**	5-HT7A	41.79	36.97	32.37	21.48	33.15
**1**	α1A	3.87	3.28	8.36	7.01	5.63
**1**	α1B	-7.47	-23.74	-16.46	1.23	-11.61
**1**	α1D	2.81	2.27	0.82	-22.05	-4.04
**1**	α2A	45.80	-37.46	54.57	33.91	24.21
**1**	α2B	11.21	19.59	24.06	5.23	15.02
**1**	α2C	33.39	46.41	37.19	33.96	37.74
**1**	β1	-30.05	-10.62	-4.52	5.01	-10.05
**1**	β2	-9.99	-15.99	-10.60	-17.07	-13.41
**1**	β3	41.93	21.51	6.77	-1.67	17.14
**1**	BZP Rat Brain Site	11.76	-3.85	-14.08	-16.43	-5.65
**1**	D1	8.16	54.70	65.67	0.96	32.37
**1**	D2	12.76	5.78	10.68	7.33	9.14
**1**	D3	31.74	-9.72	-0.56	-1.80	4.92
**1**	D4	51.73	55.57	53.96	33.86	48.78
**1**	D5	25.66	10.43	21.25	-22.77	8.64
**1**	DAT	-15.40	-9.45	3.13	12.57	-2.29
**1**	DOR	-4.33	-9.30	-18.07	-13.82	-11.38
**1**	GABAA	-23.57	-46.03	-12.82	-2.08	-21.13
**1**	H1	31.86	11.24	10.06	14.32	16.87
**1**	H2	0.68	5.75	9.50	6.96	5.72
**1**	H3	40.32	34.54	23.35	33.41	32.91
**1**	H4	-12.13	18.96	24.98	-9.59	5.56
**1**	KOR	24.34	20.24	21.32	23.01	22.23
**1**	M1	-3.84	4.98	14.78	-11.19	1.18
**1**	M2	0.66	11.53	8.81	1.96	5.74
**1**	M3	26.63	25.48	12.28	5.59	17.5
**1**	M4	-5.73	1.85	13.16	4.09	3.34
**1**	M5	10.72	11.33	-5.58	3.67	5.04
**1**	MOR	8.64	-2.63	-3.42	2.93	1.38
**1**	NET	38.64	36.85	9.56	6.78	22.96
**1**	PBR	16.61	15.65	-80.41	-40.49	-22.16
**1**	SERT	13.85	-1.68	5.40	-14.96	0.65
**1**	Sigma 1	104.29	99.60	100.33	103.90	102.03
**1**	Sigma 2	101.77	84.34	82.53	85.36	88.5
**1**	α2β2	23.09	11.22	-0.8	-11.75	5.44
**1**	α2β4	1.48	-20.02	-14.34	7.16	-6.43
**1**	α3β2	-13.33	-6.17	-16.91	3.38	-8.26
**1**	α3β4	8.89	-14.09	-22.18	-2.92	-7.58
**1**	α4β2	-11.89	-5.71	3.37	15.88	0.41
**1**	α4β2 (Rat Brain)	-4.81	1.80	6.89	-0.54	0.84

**1**	α4β4	5.70	-8.21	-10.13	-17.09	-7.43
**1**	α7	-41.00	10.22	1.46	-9.88	-9.8
**2**	5-HT1A	-12.73	-9.24	-17.62	-6.79	-11.60
**2**	5-HT1B	2.92	6.46	-7.75	7.44	2.27
**2**	5-HT1D	4.05	-2.57	1.87	2.96	1.58
**2**	5-HT1E	-22.07	-16.33	-19.68	-27.42	-21.38
**2**	5-HT2A	20.64	5.49	-0.50	6.35	8.00
**2**	5-HT2B	-40.95	-50.82	-52.30	-61.93	-51.50
**2**	5-HT2C	6.25	-0.30	0.81	19.16	6.48
**2**	5-HT3	-2.29	-2.67	-0.55	-1.69	-1.80
**2**	5-HT5A	-24.59	-21.42	12.57	8.15	-6.32
**2**	5-HT6	-1.73	7.79	3.26	-4.68	1.16
**2**	5-HT7A	24.06	24.96	23.62	13.29	21.48
**2**	α1A	2.90	7.88	13.29	12.10	9.04
**2**	α1B	-25.03	0.95	-32.59	-11.18	-16.96
**2**	α1D	-4.02	8.26	54.15	6.34	16.18
**2**	α2A	32.56	7.20	59.04	37.66	34.12
**2**	α2B	13.83	14.76	8.71	22.84	15.04
**2**	α2C	5.75	25.63	20.22	11.00	15.65
**2**	β1	-73.12	21.78	-4.14	13.58	-10.48
**2**	β2	0.29	-7.54	-10.08	3.16	-3.54
**2**	β3	36.25	17.98	15.52	32.11	25.47
**2**	BZP Rat Brain Site	6.13	-4.86	0.35	-14.25	-3.16
**2**	D1	58.42	45.62	60.36	54.99	54.85
**2**	D2	23.29	28.48	24.31	16.56	23.16
**2**	D3	87.25	89.02	79.33	86.45	85.51
**2**	D4	19.61	18.73	9.93	12.47	15.19
**2**	D5	16.60	9.90	-135.47	19.80	-22.29
**2**	DAT	-7.61	-12.64	-25.53	-4.35	-12.53
**2**	DOR	-26.85	-12.40	-20.73	-30.40	-22.60
**2**	GABAA	-25.52	-32.36	-68.5	-37.24	-40.91
**2**	H1	10.90	16.23	4.24	4.13	8.88
**2**	H2	10.83	21.86	20.76	9.72	15.79
**2**	H3	22.89	21.00	15.35	29.34	22.15
**2**	H4	26.12	26.89	-6.06	28.81	18.94
**2**	KOR	5.76	11.79	-4.73	9.50	5.58
**2**	M1	-13.15	0.08	13.31	1.55	0.45
**2**	M2	9.28	24.4	11.53	12.12	14.33
**2**	M3	15.54	4.83	12.28	-3.20	7.36
**2**	M4	13.16	0.61	19.49	-2.75	7.63
**2**	M5	26.79	3.18	19.61	6.10	13.92
**2**	MOR	1.89	3.40	-6.04	-9.37	-2.53
**2**	NET	29.85	17.85	35.57	18.71	25.50
**2**	PBR	-14.73	-28.77	-18.82	-107.66	-42.50
**2**	SERT	-0.80	3.43	0.58	-4.04	-0.21
**2**	Sigma 1	75.42	65.38	69.61	66.83	69.31
**2**	Sigma 2	50.50	32.06	-5.06	20.74	24.56
**2**	α2β2	19.89	6.81	4.53	15.63	11.72
**2**	α2β4	27.91	18.20	11.95	12.18	17.56
**2**	α3β2	-8.75	-34.41	-32.62	0.60	-18.8
**2**	α3β4	-0.48	-3.69	-16.78	2.98	-4.49
**2**	α4β2	-4.47	-13.41	-4.61	9.83	-3.17
**2**	α4β2 (Rat Brain)	14.73	0.75	12.22	0.28	7.00
**2**	α4β4	-7.73	-42.99	-52.58	-21.40	-31.18

**2**	α7	-9.88	-3.37	1.91	-8.20	-4.89
**3**	5-HT1A	37.35	12.90	-18.46	-13.64	4.54
**3**	5-HT1B	5.87	-5.20	10.26	11.96	5.72
**3**	5-HT1D	50.99	35.86	21.28	27.68	33.95
**3**	5-HT1E	-10.34	-27.58	-2.36	-1.64	-10.48
**3**	5-HT2A	1.12	4.68	-0.55	4.95	2.55
**3**	5-HT2B	-40.54	-47.04	-61.69	-56.17	-51.36
**3**	5-HT2C	6.05	-1.51	3.73	-18.15	-2.47
**3**	5-HT3	2.49	8.57	0.59	-5.11	1.64
**3**	5-HT5A	-20.08	-15.09	16.50	-13.74	-8.10
**3**	5-HT6	10.51	7.79	2.81	-11.70	2.35
**3**	5-HT7A	22.72	38.65	32.14	14.30	26.95
**3**	α1A	5.50	4.02	11.18	12.21	8.27
**3**	α1B	-11.72	-3.61	5.47	3.20	-1.67
**3**	α1D	1.35	-1.18	6.26	11.25	4.42
**3**	α2A	18.12	19.24	23.71	19.64	20.18
**3**	α2B	0.63	16.16	9.18	17.73	10.93
**3**	α2C	14.48	25.96	24.42	5.59	17.61
**3**	β1	-6.81	-25.67	21.59	24.06	3.29
**3**	β2	-10.45	-14.49	-10.31	-14.63	-12.47
**3**	β3	29.03	30.42	-0.75	11.53	17.56
**3**	BZP Rat Brain Site	4.96	2.78	-1.67	-2.00	1.02
**3**	D1	-22.04	63.55	54.99	59.42	38.98
**3**	D2	16.27	19.17	11.33	16.68	15.86
**3**	D3	-0.20	3.00	17.24	-10.7	2.34
**3**	D4	4.90	-20.02	17.90	5.99	2.19
**3**	D5	8.68	29.01	22.92	24.82	21.36
**3**	DAT	-15.63	-16.09	24.00	22.77	3.76
**3**	DOR	-24.63	-23.13	-20.64	-25.08	-23.37
**3**	GABAA	-31.38	-70.45	-63.61	-51.89	-54.33
**3**	H1	21.66	4.52	-2.77	4.13	6.89
**3**	H2	2.00	2.55	8.51	1.78	3.71
**3**	H3	56.22	52.53	49.83	48.50	51.77
**3**	H4	16.74	4.97	-7.20	-6.11	2.10
**3**	KOR	27.35	14.57	17.34	10.59	17.46
**3**	M1	9.88	4.49	11.84	16.74	10.74
**3**	M2	3.14	13.30	19.79	6.92	10.79
**3**	M3	5.02	24.14	16.87	-8.56	9.37
**3**	M4	0.48	15.77	17.88	3.09	9.31
**3**	M5	8.78	-6.19	0.38	0.50	0.87
**3**	MOR	-0.49	-6.04	-4.29	2.93	-1.97
**3**	NET	25.57	22.92	16.42	20.85	21.44
**3**	PBR	0	2.57	-20.32	-62.15	-19.98
**3**	SERT	11.20	10.02	8.94	-4.93	6.31
**3**	Sigma 1	108.66	105.22	106.87	100.53	105.32
**3**	Sigma 2	67.71	32.85	22.55	38.40	40.38
**3**	α2β2	-30.47	-5.82	-8.56	-0.04	-11.22
**3**	α2β4	34.54	26.34	20.85	14.03	23.94
**3**	α3β2	9.15	-1.39	-5.37	32.22	8.65
**3**	α3β4	34.18	47.91	51.64	21.73	38.87
**3**	α4β2	5.57	-5.57	-3.51	7.91	1.10
**3**	α4β2 (Rat Brain)	5.25	12.8	-2.30	13.45	7.30
**3**	α4β4	-11.57	-6.29	-23.08	-8.21	-12.29
**3**	α7	-11.91	-16.29	-22.80	-7.64	-14.66

**4**	5-HT1A	-39.06	-12.45	-43.25	0.54	-23.56
**4**	5-HT1B	-7.16	-7.29	12.88	4.23	0.67
**4**	5-HT1D	9.43	6.70	6.31	11.62	8.52
**4**	5-HT1E	-20.56	-15.69	-35.65	-34.37	-26.57
**4**	5-HT2A	22.05	12.23	5.81	16.55	14.16
**4**	5-HT2B	25.54	19.86	8.42	-3.18	12.66
**4**	5-HT2C	13.01	20.27	13.01	13.01	14.83
**4**	5-HT3	8.19	-5.92	7.10	-6.57	0.70
**4**	5-HT5A	0	-19.22	-2.51	6.9	-3.71
**4**	5-HT6	3.94	24.57	3.71	16.18	12.10
**4**	5-HT7A	22.16	27.09	16.10	28.44	23.45
**4**	α1A	11.50	15.40	3.66	5.71	9.07
**4**	α1B	10.79	22.92	14.36	4.37	13.11
**4**	α1D	-12.23	7.88	10.18	18.93	6.19
**4**	α2A	40.69	60.31	48.43	39.34	47.19
**4**	α2B	49.30	39.82	37.03	47.21	43.34
**4**	α2C	74.78	70.42	70.98	66.05	70.56
**4**	β1	22.92	25.02	-2.99	7.49	13.11
**4**	β2	0.34	-7.59	4.00	15.92	3.17
**4**	β3	15.36	-5.76	-7.09	-13.04	-2.63
**4**	BZP Rat Brain Site	5.46	10.66	-5.44	7.98	4.67
**4**	D1	51.99	49.45	47.21	67.91	54.14
**4**	D2	19.09	15.21	5.90	3.09	10.82
**4**	D3	27.29	49.53	53.00	39.74	42.39
**4**	D4	2.83	-12.35	-15.04	2.47	-5.52
**4**	D5	-2.82	6.32	-2.06	-10.13	-2.17
**4**	DAT	-23.45	-47.85	3.98	-3.24	-17.64
**4**	DOR	-13.2	-23.48	-8.68	-41.04	-21.6
**4**	GABAA	-32.36	-55.80	-69.47	29.18	-32.11
**4**	H1	3.25	4.77	18.75	7.99	8.69
**4**	H2	33.33	31.79	9.61	11.27	21.50
**4**	H3	20.05	11.00	5.14	2.44	9.66
**4**	H4	3.01	-5.65	-28.45	1.76	-7.33
**4**	KOR	11.91	2.26	3.11	7.81	6.27
**4**	M1	-18.54	-4.82	20.67	47.13	11.11
**4**	M2	11.41	22.15	6.33	14.60	13.62
**4**	M3	18.40	0.05	10.56	5.59	8.65
**4**	M4	0.48	7.07	-5.36	9.06	2.81
**4**	M5	12.43	-5.83	-2.42	27.03	7.80
**4**	MOR	11.34	-3.50	-5.48	1.34	0.93
**4**	NET	18.21	5.78	16.92	9.49	12.60
**4**	PBR	22.06	-3.29	-6.29	-55.88	-10.85
**4**	SERT	20.93	1.66	25.85	3.92	13.09
**4**	Sigma 1	99.93	95.64	94.71	83.48	93.44
**4**	Sigma 2	95.09	70.42	70.87	68.95	76.33
**4**	α2β2	19.44	2.09	-16.62	-24.23	-4.83
**4**	α2β4	-12.35	7.40	-15.52	23.93	0.87
**4**	α3β2	-14.92	-0.80	17.11	-17.11	-3.93
**4**	α3β4	-17.55	-17.68	-38.22	-36.04	-27.37
**4**	α4β2	-23.99	-6.94	-11.07	3.37	-9.66
**4**	α4β2 (Rat Brain)	-12.54	4.90	7.65	18.48	4.62
**4**	α4β4	-37.71	-14.21	23.20	-37.23	-16.49
**4**	α7	14.94	8.09	0.79	-6.96	4.22
**6**	Sigma 1	63.11	59.44	63.47	61.92	61.99

**6**	Sigma 2	72.63	41.84	48.89	51.8	53.8

Table S19. Inhibitor Constant (K_i) for Compounds **1**–**4**

Compound	Receptor	Ki (nM)
**1**	5-HT2B	9306.79
**1**	σ2	1292.41
**1**	σ1	13.59
**2**	5-HT2B	> 10,000
**2**	D3	5390.14
**2**	D1	> 10,000
**2**	σ1	1519.5
**3**	5-HT2B	5440.01
**3**	H3	1053.9
**3**	σ1	383.09
**3**	GABAA	1655.77
**3**	α3β4	> 10,000
**4**	5-HT1E	> 10,000
**4**	α2C	2882.04
**4**	σ2	2944.42
**4**	D1	> 10,000
**4**	σ1	376.79
**6**	σ1	2199.38
**6**	σ2	2123.24

Table S20. MM/GBSA calculated predicted free energy of binding for poses bound into the agonist receptor binding pocket. Ischnocybine A (**1**) consistently had more poses dock into the binding pocket, better predicted free binding energies, and ligand efficiencies than ischnocybine B (**3**).

Structure	Ligand	Pose	Predicted Free Energy of Binding (kcal/mol)	Ligand Efficiency (kcal/mol/heavy atom)
Agonist (PDB ID: 6DK1)	**1**	1	-52.2	-2.48
	**1**	2	-34.2	-1.63
	**1**	3	-29.6	-1.41
	**1**	4	-30.1	-1.43
	**1**	5	-24.6	-1.17
	**1**	9	-13.6	-0.65
	**2**	1	-43.9	-1.99
	**3**	5	-11.8	-0.47
	**4**	5	-31.4	-1.16
